# Survival outcomes among periviable infants: a systematic review and meta-analysis comparing different income countries and time periods

**DOI:** 10.3389/fpubh.2024.1454433

**Published:** 2024-12-30

**Authors:** Ying Xin Li, Yan Ling Hu, Xi Huang, Jie Li, Xia Li, Ze Yao Shi, Ru Yang, Xiujuan Zhang, Yuan Li, Qiong Chen

**Affiliations:** ^1^Department of Neonatology Nursing, West China Second University Hospital, Sichuan University, Chengdu, China; ^2^Key Laboratory of Birth Defects and Related Diseases of Women and Children (Sichuan University), Ministry of Education, Chengdu, China; ^3^West China School of Nursing, Sichuan University, Chengdu, China

**Keywords:** infant, extremely premature, survival, meta-analysis, systematic review

## Abstract

**Background:**

Periviable infants are a highly vulnerable neonatal group, and their survival rates are considerably affected by patient-, caregiver-, and institution-level factors, exhibiting wide variability across different income countries and time periods. This study aims to systematically review the literature on the survival rates of periviable infants and compare rates among countries with varied income levels and across different time periods.

**Methods:**

Comprehensive searches were conducted across MEDLINE, Embase, CENTRAL, and Web of Science. Cohort studies reporting survival outcomes by gestational age (GA) for periviable infants born between 22 + 0 and 25 + 6 weeks of gestation were considered. Paired reviewers independently extracted data and assessed the risk of bias and quality of evidence. Data pooling was achieved using random-effects meta-analyses.

**Results:**

Sixty-nine studies from 25 countries were included, covering 56,526 live births and 59,104 neonatal intensive care unit (NICU) admissions. Survival rates for infants born between 22 and 25 weeks of GA ranged from 7% (95% CI 5–10; 22 studies, *n* = 5,658; low certainty) to 68% (95% CI 63–72; 35 studies, *n* = 21,897; low certainty) when calculated using live births as the denominator, and from 30% (95% CI 25–36; 31 studies, *n* = 3,991; very low certainty) to 74% (95% CI 70–77; 48 studies, *n* = 17,664, very low certainty) for those admitted to NICUs. The survival rates improved over the two decades studied; however, stark contrasts were evident across countries with varying income levels.

**Conclusion:**

Although the survival rates for periviable infants have improved over the past two decades, substantial disparities persist across different economic settings, highlighting global inequalities in perinatal health. Continued research and collaborative efforts are imperative to further improve the global survival and long-term outcomes of periviable infants, especially those in Low- and Middle-Income Countries.

**Systematic review registration:**

PROSPERO, CRD42022376367, available from: https://www.crd.york.ac.uk/prospero/display_record.php?ID=CRD42022376367.

## 1 Introduction

Periviable infants, those born between 22 + 0 and 25 + 6 weeks of gestational age (GA), constitute 0.05% of all births (1, 2). Survival rates for these infants have improved significantly due to advancements in medical technology, neonatal care, and perinatal management. However, survival rates vary widely across countries, from 0 to 40% at 22 weeks GA and 4 to 82% at 25 weeks GA (3–7), reflecting disparities in medical care, treatment attitudes, and economic conditions.

The decision to administer life-saving treatment or palliative care for periviable infants is ethically challenging (8, 9). A notable lack of consensus exists among international guidelines regarding the appropriate course of action for these infants (10–16). A systematic review revealed that 68% of the guidelines recommended palliative care for infants at 22 weeks GA, while 65% endorsed active treatment for infants at 25 weeks GA (17). The most significant disparity in recommendations occurs at 23 and 24 weeks GA, with some guidelines suggesting a case-by-case approach considering individual factors such as birth weight and parental preferences (17). This highlights the need for up-to-date data to guide shared decision-making among policymakers, healthcare providers, and families.

Previous studies on periviable infants have predominantly focused on High-Income Countries (HICs), often overlooking the situation in Low- and Middle-Income Countries (LMICs) (18). Although preterm survival rates have increased in HICs, preterm newborns still die in many LMICs (19). This bias risks not only overestimating the global survival rates of periviable infants but also leaves LMICs without a reliable data basis for shared decision-making. For instance, Myrhaug et al.’s and Salihu et al.’s reviews solely incorporated data from HICs, neglecting survival outcomes in less affluent regions (18, 20). Additionally, medical practices have undergone substantial transformations, such as the introduction of pulmonary surfactants and prenatal steroids between 1990 and 2000 (21, 22), as well as improvements in ventilation and nutrition support from 2000 to 2010, both of which potentially exerted positive effects on periviable infants’ survival rates (23, 24). Nevertheless, the scarcity of aggregated data on survival outcomes in LMICs and the lack of inclusion of the most recent studies in prior literature hinder our ability to grasp the full scope of global disparities and temporal trends in survival rates for this vulnerable population.

Therefore, this study aims to conduct a systematic review and meta-analysis of global survival rates among periviable infants, comparing outcomes across countries of varied income levels and different time periods. By incorporating the most updated data from diverse nations, we seek to shed light on the status of periviable infants in LMICs and track how this status evolves over time, providing essential data to aid healthcare providers and families in making informed decisions.

## 2 Methods

We registered this systematic review with PROSPERO (https://www.crd.york.ac.uk/prospero/display_record.php?RecordID=376367) and followed the Meta-analysis of Observational Studies in Epidemiology (MOOSE) checklist and the Preferred Reporting Items for Systematic Reviews and Meta-Analyses (PRISMA) statement for reporting (25, 26).

### 2.1 Search strategy and selection criteria

We searched MEDLINE, Embase, CENTRAL, and Web of Science initially in September 2022, with a subsequent update on August 16, 2023, and manually checked the reference list of all identified articles. Our search strategy was restricted to include articles published in English since January 1, 2000, to offer a comprehensive overview of the most relevant and recent findings in the field. The detailed search strategy can be found in Supplementary Table 3.

We specifically focused on cohort studies, both with and without controls, that included periviable infants born between 22 + 0/7 and 25 + 6/7 weeks of GA, with an emphasis on reporting survival outcomes according to GA. The GA had to be determined using ultrasound, the last menstrual period, or a combination of both. We excluded studies with duplicate data, cohorts with births prior to the year 2000, and studies that did not report results for each GA group separately. The primary outcome of our study was GA-related survival, assessed using two distinct metrics: survival as a proportion of live births and survival among infants admitted to NICUs. We defined survival as infants alive at NICU discharge or at any point between 1 and 3 years of age. When multiple survival endpoints were reported within a study, we prioritized data from the latest follow-up assessment for analysis.

The literature screening process began with two independent reviewers scrutinizing titles and abstracts for potential literature and was followed by a thorough examination of the full-text articles against the predefined inclusion and exclusion criteria to determine their eligibility. Discrepancies were resolved by involving a third reviewer to reach a consensus.

### 2.2 Data extraction and quality assessment

We devised a structured literature extraction form to methodically gather essential information from selected studies, including the study’s country of origin, author(s), title, publication year, data source, cohort’s birth year, sample size, GA, study design, outcome measures, timing of outcome assessment, number of live births, NICU admissions, survivals, and deaths. We employed a double-data entry process to ensure data accuracy. In instances where duplicate publications utilized identical infant cohorts or shared overlapping datasets, the most recent and comprehensive article was selected as the primary reference for our study. The Newcastle-Ottawa Scale (NOS) was utilized to assess the quality of the included studies, assigning up to 9 stars to signify the quality of a study, with more stars indicating higher quality (27). Based on this scale, studies were categorized into three levels of bias risk: high (0–3 stars), moderate (4–6 stars), and low (7–9 stars).

### 2.3 Quality of evidence

The evaluation of the evidence quality was performed using the GRADE approach, facilitated by the GRADEpro Guideline Development Tool (GDT). According to the GRADE system, the quality of evidence can be classified into four levels: high, moderate, low, and very low. This classification is determined on the design of the study, taking into account five factors that may degrade evidence quality (risk of bias, inconsistency, indirectness, imprecision, and publication bias) and three upgrading factors (large effect size, dose–response gradient, and plausible residual confounding) (28, 29).

### 2.4 Data analysis

Survival rates were calculated across different GA, utilizing the number of live births and NICU admissions as separate denominators for each calculation. The DerSimonian-Laird random-effects model with logit transformations was employed for the computation of pooled estimates to account for the inherent heterogeneity across included studies (30). In studies reporting extreme event rates of 0% or 100%, an adjustment was applied by adding 0.5 to the numerator and 1 to the denominator prior to executing the logit transformation. The effect size for each study was described through individual study proportions, with confidence intervals (CIs) derived using the score method. We assessed heterogeneity among these study proportions using the Higgins *I*^2^ statistic (31), complemented by visual inspection of forest plots.

For sensitivity analysis, a leave-one-out meta-analysis was conducted to evaluate the robustness of our results. This method involves removing one study at a time and recalculating the overall effect size to check the stability of the findings (32). Furthermore, to explore potential sources of heterogeneity, meta-regression was performed to assess the effects of the publication year, sample size, countries, and bias risk of studies on survival rates. Publication bias was assessed using the contour-enhanced funnel plot and execution of Egger test. We carried out subgroup analyses to examine the disparities in periviable infants’ survival between HICs and LMICs. The income classification was based on the 2022 gross national income (GNI) per capita, as defined by the World Bank Atlas method. According to this classification, LMICs include: (1) low income (GNI of $1,135 or less); (2) lower middle income (GNI between $1,136 and $4,465); (3) upper middle income (GNI between $4,466 and $13,845), while HICs are defined as having a GNI of $13,846 or more (33). Additionally, to assess the influence of temporal changes on survival rates, we divided the analysis into three Epochs: before 2010 (Epoch 1), from 2011 to 2019 (Epoch 2), and from 2020 onwards (Epoch 3). These Epochs were chosen to capture potential changes in survival rates over time due to advancements in medical technology and neonatal care practices. All calculations were performed using R, version 4.3.2.

## 3 Results

### 3.1 Study selection and characteristics

**Figure 1:**
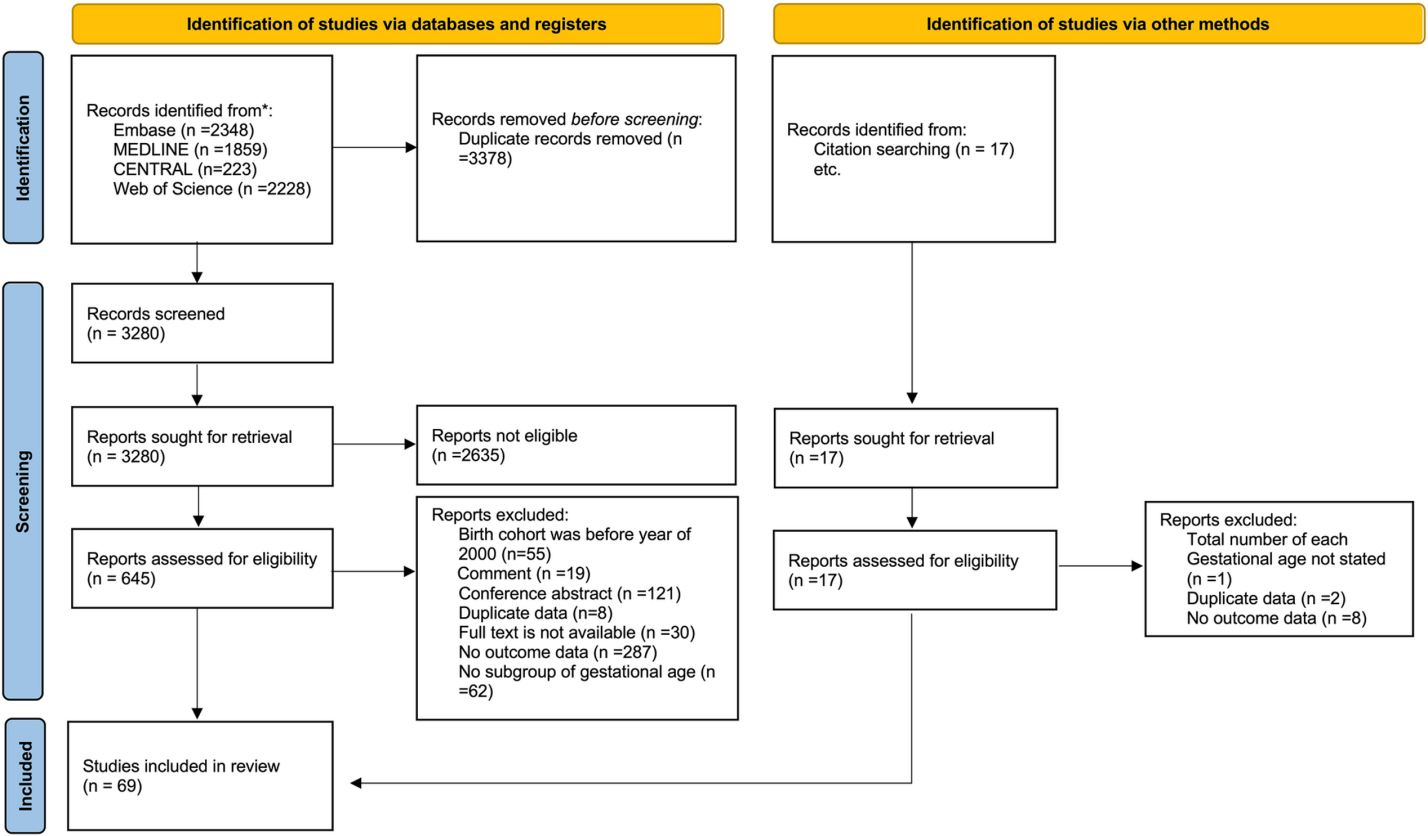
The PRISMA 2020 flow diagram.

The search strategy yielded 6,658 database records, and 17 records were identified using other methods (Figure 1). After the primary screening and eligibility assessment, 69 studies met the inclusion criteria, including a total of 56,526 live periviable births and 59,104 admitted to NICU (3–6, 34–98). Among them, 39 studies reported survival outcomes using live births as the denominator, and 59 studies reported survival outcomes using NICU admissions as the denominator. The included studies were conducted in 25 countries: the United States with 16 studies (4, 34–48), Australia with 9 (49–57), United Kingdom with 5 (58–62), China and Germany each with 4 (63–70), and the remaining countries contributing 1 to 3 studies each. The cohort’s birth year spanned from 2000 to 2020, while the year of publication ranged from 2008 to 2023. The studies fell into three epochs: 7 studies from Epoch 1, 46 studies from Epoch 2, and 16 studies from Epoch 3. The detailed characteristics of the included studies are shown in Table 1. Regarding the results of the quality assessment (Supplementary Table 4), 7 studies had a low risk of bias, 56 studies had a moderate risk of bias, and 6 studies had a high risk of bias. The GRADE results are provided in a summary of findings table, presented in Supplementary Table 5.

**Table 1:** Characteristics of included studies (*N* = 69).

Author year (country)	Birth year	Infants’ GA	Study design	Population-based studies	Outcomes	ROB
Lavilla 2022 (USA) (34)	2012–2020	22–28	retrospective cohort study	N	neonatal survival	moderate
Bell 2022 (USA) (35)	2013–2018	22–28	prospective cohort study	Y	survival to discharge/36 GA	low
Czarny 2021 (USA) (36)	2007–2013	22–23	retrospective cohort study	Y	neonatal survival, survival at 1 year	moderate
Watkins 2020 (USA) (37)	2006–2015	22–25	retrospective cohort study	N	survival to discharge	moderate
Puia-Dumitrescu 2020 (USA) (38)	2006–2016	22–24	retrospective cohort study	Y	survival to discharge	moderate
Younge 2017 (USA) (39)	2000–2003; 2004–2007; 2008–2011	22–24	prospective cohort study	Y	survival to 18-22 m	low
Younge 2016 (USA) (40)	2005–2011	22–24	retrospective cohort study	N	death (17-25 m)	moderate
Manuck 2016 (USA) (41)	2008–2011	23–36	cohort study	Y	neonatal death	moderate
Anderson 2016 (USA) (42)	2007–2011	22–28	retrospective cohort study	Y	survival at 1 year	moderate
Stoll 2015 (USA) (43)	2003–2007; 2008–2012	22–28	prospective cohort study	Y	survival to discharge,	low
Malloy 2015 (USA) (44)	2000; 2010	22–28	prospective cohort study	Y	survival to discharge	low
Nguyen 2012 (USA) (45)	2001–2010	23	retrospective cohort study	Y	survival to discharge/at 1 year	moderate
Kyser 2012 (USA) (46)	2000–2009	22–25	retrospective cohort study	N	survival to discharge	moderate
Zayek 2011 (USA) (4)	2003–2008	22–26	retrospective cohort study	N	survival to discharge, neonatal survival	moderate
Lee 2010 (USA) (47)	2005–2008	22–25	prospective cohort study	Y	death before discharge	moderate
Bode 2008 (USA) (48)	2005–2006	≤30	prospective cohort study	N	mortality at discharge	moderate
Sinclair 2019 (Australia) (49)	2000–2011	22–26	prospective cohort study	Y	survival to discharge	moderate
Ireland 2019 (Australia) (50)	2010–2016	22–27	retrospective cohort study	N	survival to discharge	moderate
Bolisetty 2018 (Australia) (51)	2007–2012	23–28	retrospective cohort study	Y	survival to discharge	moderate
Sharp 2018 (Australia) (52)	2004–2010	22–24	retrospective cohort study	N	survival to discharge	moderate
Atwell 2018 (Australia) (53)	2010–2013	23–25	cohort study	Y	survival to discharge	moderate
Boland 2016 (Australia) (54)	2010–2011	22–27	prospective cohort study	Y	survival to discharge, survival at 1 year	low
Thompson 2016 (Australia) (55)	2001–2011	23–25	retrospective cohort study	N	survival to discharge	moderate
Gunn 2012 (Australia) (56)	2004–2009	23–25	retrospective cohort study	N	survival to discharge	moderate
Doyle 2010 (Australia) (57)	2005	22–27	cohort study	Y	survival at 2 years	moderate
Morgan 2021 (UK) (58)	2011–2013	22–26	retrospective cohort study	N	neonatal death; survival to discharge	high
Seaton 2013 (UK) (59)	2001–2010	22–25	retrospective cohort study	Y	survival to discharge	moderate
Costeloe 2012 (UK) (60)	2006	22–26	prospective cohort study	Y	survival to discharge, neonatal survival	moderate
Rattihalli 2010 (UK) (61)	2001–2003	20–25	prospective cohort study	Y	survival to discharge; survival at 2 years	moderate
Field 2008 (UK) (62)	2000	22–25	prospective cohort study	Y	survival to discharge	moderate
Zhang 2022 (China) (63)	2010–2019	24–27	prospective cohort study	Y	death before discharge/40 GA	moderate
Zhu 2021 (China) (64)	2010–2019	21–27	retrospective cohort study	Y	survival to discharge	moderate
Wu 2019 (China) (65)	2008–2017	22–27	prospective cohort study	Y	survival to discharge	moderate
Chang 2018 (China, Taiwan) (66)	2007–2011	22–26	prospective cohort study	Y	survival to discharge	moderate
Humberg 2020 (Germany) (67)	2011–2016	22–28	prospective cohort study	Y	survival to discharge	moderate
Mehler 2016 (Germany) (68)	2010–2014	22–23	retrospective cohort study	N	survival to discharge	moderate
Stichtenoth 2012 (Germany) (69)	2010	VLBWIs	prospective cohort study	Y	survival to discharge	moderate
Kutz 2009 (Germany) (70)	2000–2004	22–25	cohort study	N	survival to discharge	high
Beek 2022 (The Netherlands) (71)	2018–2020	24–26	cohort study	Y	survival to discharge, survival at 2 years	low
Beek 2021 (The Netherlands) (72)	2011–2017; 2007–2009	24–26	cohort study	Y	survival to discharge	moderate
Zegers 2016 (The Netherlands) (73)	2000–2011	24–29	prospective cohort study	Y	neonatal mortality	moderate
Thomas 2020 (Canada) (74)	2005–2014	23–26	retrospective cohort study	N	death (before discharge, 44 GA), neonatal mortality	moderate
Shah 2020 (Canada) (75)	2010–2017	22–25	retrospective cohort study	Y	survival to discharge	moderate
Crane 2015 (Canada) (76)	2005–2011	23	prospective cohort study	Y	Neonatal mortality, survival to discharge	high
Chen 2016 (Switzerland) (77)	2000–2012	23–31	prospective cohort study	Y	survival to discharge	moderate
Morgillo 2014 (Switzerland) (78)	2000–2009	23–27	retrospective cohort study	N	neonatal survival	moderate
Schlapbach 2012 (Switzerland) (79)	2000–2008	24–27	prospective cohort study	Y	survival at 2 years	moderate
Aronsson 2023 (Sweden) (80)	2012–2016	22–25	retrospective cohort study	N	neonatal mortality, survival at 1 year	moderate
Express group 2009 (Sweden) (81)	2004–2007	22–26	prospective cohort study	Y	survival to discharge, survival to 1 year	low
Kim 2018 (Korea) (82)	2001–2016	21–23	retrospective cohort study	N	neonatal mortality	moderate
Shim 2015 (Korea) (83)	2013–2014	VLBWIs	cohort study	Y	survival to discharge	moderate
Goya 2015 (Spain) (84)	2005–2011	23–25	retrospective cohort study	N	neonatal death	moderate
Rodrigo 2015 (Spain) (85)	2002–2006; 2007–2011	22–26	prospective cohort study	Y	survival to discharge	moderate
Wang 2011 (Norway) (86)	2004–2007	24–27	retrospective cohort study	N	survival to discharge, neonatal survival	moderate
Stensvold 2017 (Norway) (87)	2013–2014	22–26	prospective cohort study	Y	survival to 1 year	moderate
Kiechl-Kohlendorfer 2019 (Austria) (88)	2011–2016	23–31	prospective cohort study	Y	death before discharge	moderate
Ancel 2015 (France) (3)	2011	22–34	prospective cohort study	Y	survival to discharge	low
Uccella 2015 (Italy) (89)	2003–2010	23–25	retrospective cohort study	N	survival to discharge	high
Ishii 2013 (Japan) (90)	2003–2005	22–25	cohort study	N	survival to discharge; survival at 3 years	moderate
Berry 2017 (New Zealand) (91)	2003–2012	23–24	cohort study	N	Survival to discharge, survival at 2 years	moderate
Fajolu 2019 (Nigeria) (5)	2010–2017	24–27	retrospective cohort study	N	survival to discharge	moderate
Rahman 2015 (Oman) (92)	2006–2013	23–26	retrospective cohort study	N	survival to discharge	high
Suciu 2017 (Romania) (93)	2007–2010; 2011–2014	25–28	cohort study	N	death (death, 48 h, 3–6d,7-36d)	high
Agarwal 2014 (Singapore) (6)	2000–2009	23–28	prospective cohort study	N	survival to discharge	moderate
Musiime 2021 (South Africa) (94)	2016	23–34	retrospective cohort study	N	survival to discharge	moderate
Park 2019 (South Korea) (95)	2014–2016	23–24	retrospective cohort study	Y	mortality (7d, 8-28d, after 28d)	moderate
Piriyapokin 2020 (Thailand) (96)	2005–2015	23–25	retrospective cohort study	N	survival to discharge	high
Kulali 2019 (Turkey) (97)	2011–2015	22–25	retrospective cohort study	N	survival to discharge	moderate
Smith 2017 (Belgium, France, Italy, Portugal, UK) (98)	2011–2012	22–25	prospective cohort study	Y	survival to discharge	moderate

GA, gestational age; ROB, risk of bias; VLBWIs, very low birth weight infants. N, single-center study; Y, population-based cohort study.

### 3.2 Survival rates of periviable infants at different GA

**Figure 2:**
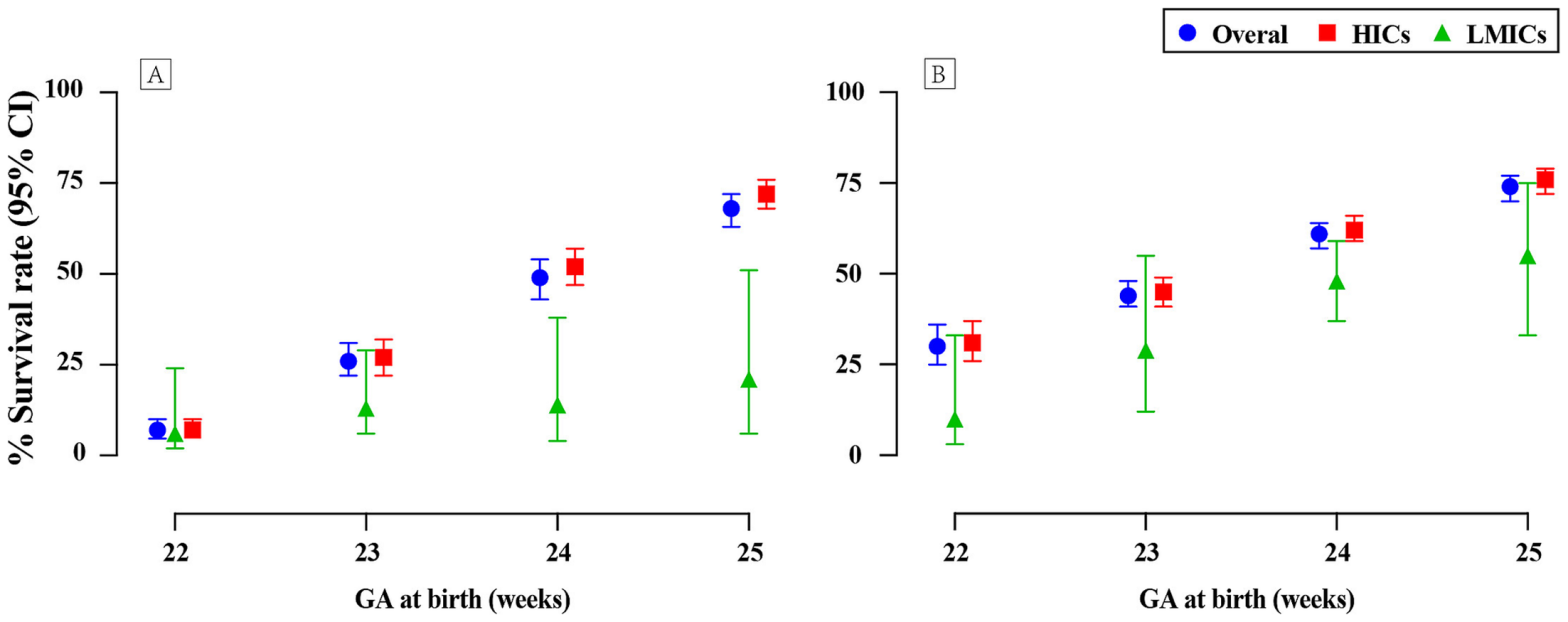
Survival rates of periviable infants at different GA across countries with varied income levels: **(A)** Live births; **(B)** NICU admissions.

The survival outcomes among periviable births have been observed to be positively correlated with GA, as depicted in Figure 2 and Supplementary Table 6. Substantial disparities emerged when analyzing survival rates using both live births and NICU admissions as denominators. Specifically, survival rates calculated based on infants admitted to NICUs were higher compared to those on live births, with the difference being particularly notable for infants born at 22 and 23 weeks of GA.

**Figure 3:**
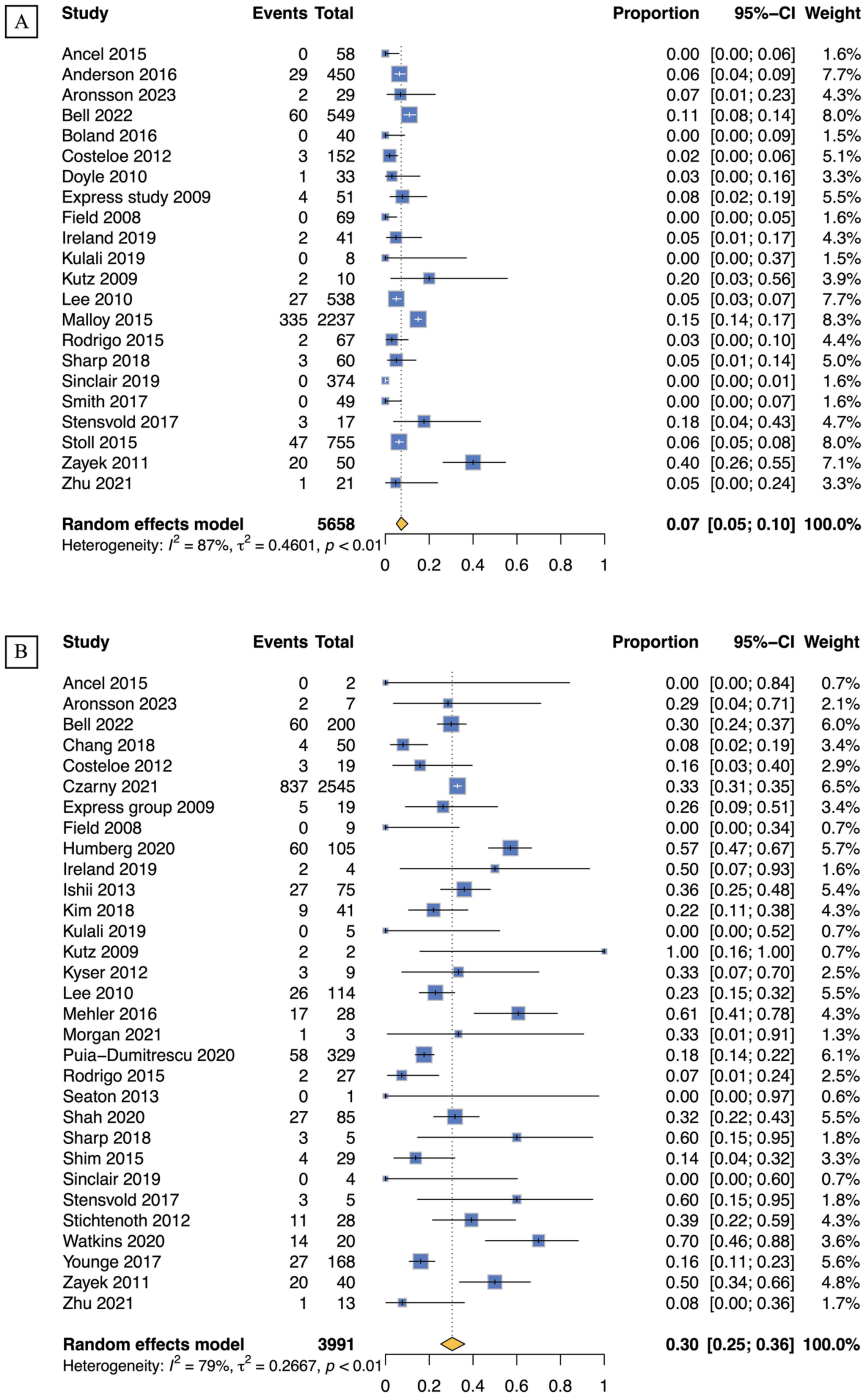
Survival rates of periviable infants born at 22 weeks of GA: **(A)** Live births; **(B)** NICU admissions.

For infants at GA of 22, 23, 24, and 25 weeks, survival rates calculated with live births as the denominator were 7% (95% CI 5–10; 22 studies, *n* = 5,658; low certainty), 26% (95% CI 22–31; 32 studies, *n* = 10,767; low certainty), 49% (95% CI 43–54; 37 studies, *n* = 18,204; low certainty), and 68% (95% CI 63–72; 35 studies, *n* = 21,897; low certainty), respectively. In contrast, when evaluating the same GA groups with NICU admissions as the denominator, survival rates were found to be 30% (95% CI 25–36; 31 studies, *n* = 3,991; very low certainty), 44% (95% CI 41–48; 50 studies, *n* = 17,379; very low certainty), 61% (95% CI 57–64; 52 studies, *n* = 20,070; very low certainty), and 74% (95% CI 70–77; 48 studies, *n* = 17,664; very low certainty). Forest plots for GA of 22 weeks are presented in Figure 3, with remaining plots in Supplementary Figures 5–10.

### 3.3 Survival rates of periviable infants across countries with varied income levels

A pronounced disparity in the survival rates of periviable infants was observed between LMICs and HICs, with the latter consistently exhibiting more favorable outcomes (Figure 2; Supplementary Table 6). At 22 weeks of gestation, the survival rates in LMICs were notably low, with a mere 6% for live births and 10% for NICU admissions, in contrast to the comparatively higher rates of 7 and 31%, respectively, in HICs. This gap widened further at 23 weeks, with LMICs experiencing survival rates of 13% for live births and 29% for NICU admissions, which were substantially lower than the rates of 27 and 45% observed in HICs. Despite a modest improvement in survival rates in LMICs at 24 weeks, with 14% for live births and 48% for NICU admissions, these rates remained substantially lower than those in HICs, which were 52 and 62%, respectively. The disparity persisted even at 25 weeks of gestation, with LMICs exhibiting markedly lower rates compared to HICs: 21% vs. 72% for live births and 55% vs. 76% for NICU admissions.

### 3.4 Survival rates of periviable infants across different epochs

**Figure 4:**
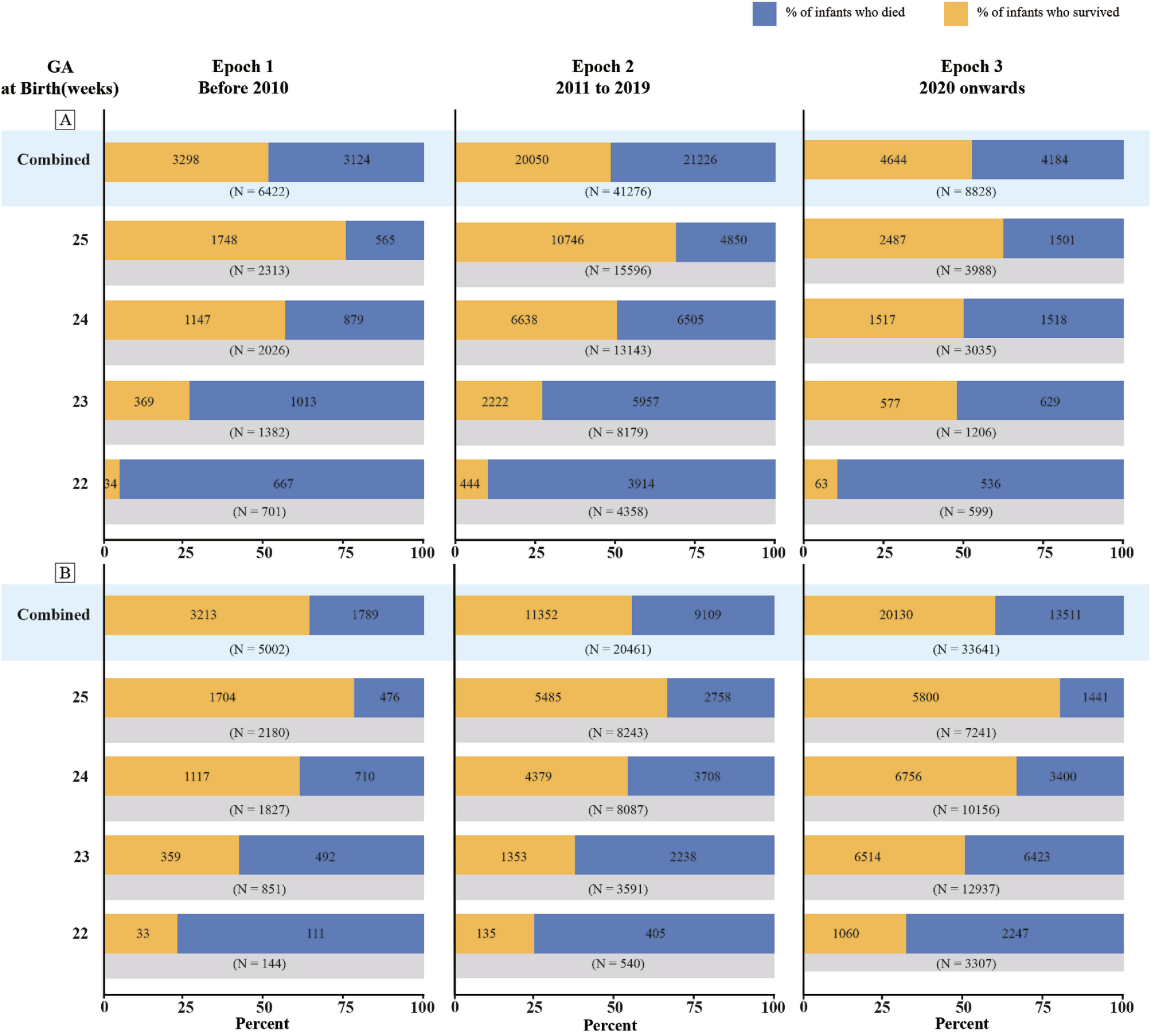
Survival rates of periviable infants at different GA across different epochs: **(A)** Live births; **(B)** NICU admissions.

Marked disparities in survival rates were observed across different epochs, with the most pronounced improvements evident for NICU admissions (Figure 4; Supplementary Table 7). For infants admitted to NICUs at 22 weeks of gestation, the survival rate increased from 25% in Epoch 1 to 34% in Epoch 3. This improvement was even more substantial for those at 23 weeks, with the survival rate climbing from 36% in Epoch 1 to 53% in Epoch 3. Infants at 24 weeks of gestation also experienced improved survival rates, increasing from 56 to 67%, while those at 25 weeks saw a more modest increase from 74 to 80%. When considering live births, the most striking disparity was observed in infants at 23 weeks of gestation, with the survival rate in Epoch 3 nearly doubling that of Epoch 1 (40% vs. 21%). However, this trend of improvement was less pronounced for infants at 24 and 25 weeks of gestation. Forest plots for the subgroup analyses stratified by different epochs are shown in Supplementary Figures 19–26.

In addition, sensitivity analyses using a leave-one-out cross-validation approach did not reveal any significant deviations from the primary analyses (Supplementary Figures 27–34). The results of the meta-regression analysis indicated an association between publication year and survival rates, as well as significant variation in survival rates across different countries (*p* < 0.05), as shown in Supplementary Tables 8–15. A low likelihood of publication bias was observed (*p* > 0.05), as shown in Supplementary Table 16 and Supplementary Figures 35 –42.

## 4 Discussion

This systematic review and meta-analysis incorporated 69 studies from 25 countries, encompassing a wide range of income levels and temporal scopes, and synthesized data from 56,526 live births and 59,104 NICU admissions. The study findings corroborate previous research, confirming that survival rates for periviable infants incrementally increase with each additional week of gestation (43, 99, 100). Notably, a significant disparity in survival rates was observed depending on whether live births or NICU admissions were used as the denominator. Specifically, for infants at 22 and 23 weeks’ GA, using NICU admissions as the denominator revealed a marked improvement in survival rates. This finding underscores the crucial role of intensive care in enhancing the survival prospects of these vulnerable infants while simultaneously highlighting the disparities in the proactive provision of NICU treatments across different GAs.

### 4.1 Global disparities in neonatal intensive care

Importantly, our findings reveal profound geographical disparities in the survival outcomes of periviable infants, with higher survival rates in HICs compared to LMICs. This gap is largely due to unequal access to advanced medical technologies, skilled healthcare workforce, and neonatal care facilities, as well as differences in national management strategies. For instance, in the United States, the American College of Obstetricians and Gynecologists (ACOG) and the Society of Maternal-Fetal Medicine (SMFM) advocate for the consideration of resuscitation efforts for infants at 22 and 23 weeks of gestation, and typically recommend such measures at 24 and 25 weeks (101). Similarly, the Canadian Pediatric Society recommends that resuscitation should be provided at 25 weeks, and following thorough discussions with parents, could be considered for neonates at 23 and 24 weeks (102). Conversely, in Nigeria, the official age of viability is 28 weeks, leading to completely divergent practices (5). In India, decisions on active care depend on parental preferences, reflecting the complexities of the healthcare system (103). Additionally, the gap may also be influenced by societal attitudes on extreme preterm birth and the availability of follow-up services. In several HICs, decisions have been made not to resuscitate extremely preterm infants, particularly those born at 22 weeks, due to concerns over long-term quality of life and high associated costs. This, combined with differences in follow-up care availability, contributes to the survival rate disparity. The stark contrast in survival rates highlight the need for international efforts to elevate neonatal care infrastructure and standards in LMICs, aiming to bridge the survival gap and ensure equitable opportunities for all infants.

Furthermore, when using live births as the denominator, our subgroup analysis for HICs aligns with previous findings by Myrhaug et al. on the survival rates of infants born between 22 and 25 weeks of GA (18) and closely resembles the ACOG and SMFM consensus on periviable birth (15). However, when employing NICU admissions as the denominator, our findings for infants at 22, 23, and 24 weeks GA were modestly higher than those of earlier studies (18, 104). These differences likely reflect the inclusion of more recent cohorts in our analysis and ongoing advancements in neonatal care (18, 104). Moreover, our finding for 22 weeks GA mirrors that of a previous systematic review focusing on HICs where proactive care was provided (105). In contrast to that review, our study is novel in encompassing a broader range of GAs and more recent cohorts, providing a comprehensive and up-to-date assessment of the survival outcomes for periviable infants.

### 4.2 Temporal trends, neonatal care advances, and impact of COVID-19

Our findings suggest substantial disparities in survival rates across different epochs, with a more pronounced improvement for infants admitted to NICUs, highlighting advancements in neonatal care. The progress can likely be attributed to the refinement of neonatal care technologies and practices. The similar upward trends in survival rates for live births at 22 and 23 weeks GA may reflect recent improvements in perinatal healthcare. However, this positive trend in survival rates was not observed for live births at 24 and 25 weeks GA, which may be attributed to the limited number of studies for these GAs in Epoch 3, as well as a larger proportion of studies from LMICs.

The COVID-19 pandemic may have influenced survival rates during Epoch 3. Public health measures like lockdowns and curfews have been linked to reductions in extreme preterm birth rates (9). Changes in medical resource allocation and NICU practices during the pandemic may have also affected survival. Further research is needed to explore the pandemic’s effects and monitor post-pandemic survival trends globally.

### 4.3 Strengths and limitations

The strengths of this study lie in delivering an up-to-date, comprehensive global analysis of survival outcomes for periviable infants, employing rigorous methodology, and uncovering survival disparities in LMICs. Despite these strengths, several limitations should warrant acknowledgment. First, the risk of bias was low in only 10.1% of studies, moderate in 81.2%, and high in 8.7%, and the evidence quality was assessed as very low to low based on the GRADE criteria, largely due to the observational design of the included studies (28). Additionally, differences in study design, such as population-based cohort studies versus single-center studies, may impact generalizability. While population-based studies (58%) provide broader applicability, single-center studies (42%) are more prone to selection bias. Second, the significant heterogeneity across the included studies should be taken into account when interpreting and applying the findings. This heterogeneity may stem from differences in the countries and regions where the studies were conducted, the time periods covered by the cohorts, and variations in follow-up durations. Third, the division of the studies into three Epochs for temporal analysis also introduces challenges, particularly for Epoch 3 (post-2020), which included fewer studies, potentially compromising its representativeness. Finally, there was a scarcity of studies from LMICs; the limited data from these regions may lead to unreliability and instability in the pooled results for the LMIC subgroup. Future research should endeavor to continuously monitor and update data beyond 2023, with a particular emphasis on obtaining more robust data from LMICs, to more accurately capture the evolving survival outcomes of periviable infants.

## 5 Conclusion

The study underscores the intricate dependence of periviable infants’ survival on GA, the quality of healthcare they receive, and the evolution of medical practices over time. The findings reveal stark inequalities in perinatal outcomes across various economic settings, highlighting the profound impact of socioeconomic disparities on the survival of the most vulnerable infants. While the results also showcase the significant progress made in neonatal care over the past two decades, particularly for infants born at 22 and 23 weeks of gestation in HICs, it is evident that these improvements have not been universally applied, leading to persistent survival disparities between HICs and LMICs.

Our research has profound implications for both clinical practice and policy formulation. For healthcare providers, the insights gained can inform the development of focused care strategies and support the shared decision-making process with families regarding the pursuit of intensive treatments for preterm infants. At the policy level, our results underscore the urgent need for targeted interventions and resource allocation to reduce the survival disparities between HICs and LMICs, promoting equity in maternal and perinatal health. Policymakers in LMICs might consider adjusting the lower threshold for resuscitation based on these findings, while also investing in the improvement of neonatal care infrastructure and practices to ensure timely, high-quality, and respectful care for all mothers and their preterm infants. Continued research and collaborative efforts, with a particular focus on addressing the root causes of perinatal health disparities and enhancing neonatal care in LMICs, are imperative to further improve the survival and long-term outcomes of periviable infants on a global scale.

## Data Availability

The raw data supporting the conclusions of this article will be made available by the authors, without undue reservation.
