# Supplementary material for: Survival outcomes among periviable infants: a systematic review and meta-analysis comparing different income countries and time periods
Source: Front Public Health. 2024 Dec 30;12:1454433. doi: 10.3389/fpubh.2024.1454433 (PMC11726316; doi:10.3389/fpubh.2024.1454433)
Supplement: Supplementary file 1 [file Data_Sheet_1.pdf]

Supplementary Material

1.1	Supplementary Table 2 PRISMA Checklist	1
1.2	Supplementary Table 3 Search strategy	4
1.3	Supplementary Table 4 Risk bias of included studies	8
1.4	Supplementary Table 5 Summary of findings table	12
1.5	Supplementary Table 6	16
1.6	Supplementary Table 7	17
1.7	Supplementary Table 8	18
1.8	Supplementary Table 9	19
1.9	Supplementary Table 10	20
1.10	Supplementary Table 11	21
1.11	Supplementary Table 12	22
1.12	Supplementary Table 13	23
1.13	Supplementary Table 14	25
1.14	Supplementary Table 15	27
1.15	Supplementary Table 16	29
1.16	Supplementary Figure 5	30
1.17	Supplementary Figure 6	31
1.18	Supplementary Figure 7	32
1.19	Supplementary Figure 8	33
1.20	Supplementary Figure 9	34
1.21	Supplementary Figure 10	35
1.22	Supplementary Figure 11	36
1.23	Supplementary Figure 12	37

1.24	Supplementary Figure 13	38
1.25	Supplementary Figure 14	39
1.26	Supplementary Figure 15	40
1.27	Supplementary Figure 16	41
1.28	Supplementary Figure 17	42
1.29	Supplementary Figure 18	43
1.30	Supplementary Figure 19	44
1.31	Supplementary Figure 20	45
1.32	Supplementary Figure 21	46
1.33	Supplementary Figure 22	47
1.34	Supplementary Figure 23	48
1.35	Supplementary Figure 24	49
1.36	Supplementary Figure 25	50
1.37	Supplementary Figure 26	51
1.38	Supplementary Figure 27	52
1.39	Supplementary Figure 28	53
1.40	Supplementary Figure 29	54
1.41	Supplementary Figure 30	55
1.42	Supplementary Figure 31	56
1.43	Supplementary Figure 32	57
1.44	Supplementary Figure 33	58
1.45	Supplementary Figure 34	59
1.46	Supplementary Figure 35	60
1.47	Supplementary Figure 36	60
1.48	Supplementary Figure 37	61

1.49 Supplementary Figure 38 61

1.50 Supplementary Figure 39 62

1.51 Supplementary Figure 40 62

1.52 Supplementary Figure 41 63

1.53 Supplementary Figure 42 63

1 Supplementary Tables

1.1 Supplementary Table 2 PRISMA Checklist

Section and Topic	Item #	Checklist item	Location where item is reported
**TITLE**			
Title	1	Identify the report as a systematic review.	Title
**ABSTRACT**			
Abstract	2	See the PRISMA 2020 for Abstracts checklist.	Abstract
**INTRODUCTION**			
Rationale	3	Describe the rationale for the review in the context of existing knowledge.	Introduction
Objectives	4	Provide an explicit statement of the objective(s) or question(s) the review addresses.	Introduction
**METHODS**			
Eligibility criteria	5	Specify the inclusion and exclusion criteria for the review and how studies were grouped for the syntheses.	2.1 Search strategy and selection criteria
Information sources	6	Specify all databases, registers, websites, organisations, reference lists and other sources searched or consulted to identify studies. Specify the date when each source was last searched or consulted.	2.1 Search strategy and selection criteria
Search strategy	7	Present the full search strategies for all databases, registers and websites, including any filters and limits used.	Supplementary Table 3
Selection process	8	Specify the methods used to decide whether a study met the inclusion criteria of the review, including how many reviewers screened each record and each report retrieved, whether they worked independently, and if applicable, details of automation tools used in the process.	2.1 Search strategy and selection criteria
Data collection process	9	Specify the methods used to collect data from reports, including how many reviewers collected data from each report, whether they worked independently, any processes for obtaining or confirming data from study investigators, and if applicable, details of automation tools used in the process.	2.2 Data extraction and quality assessment
Data items	10a	List and define all outcomes for which data were sought. Specify whether all results that were compatible with each outcome domain in each study were sought (e.g. for all measures, time points, analyses), and if not, the methods used to decide which results to collect.	2.1 Search strategy and selection criteria
	10b	List and define all other variables for which data were sought (e.g. participant and intervention characteristics, funding sources). Describe any assumptions made about any missing or unclear information.	2.2 Data extraction and quality

Section and Topic	Item #	Checklist item	Location where item is reported
Study risk of bias assessment	11	Specify the methods used to assess risk of bias in the included studies, including details of the tool(s) used, how many reviewers assessed each study and whether they worked independently, and if applicable, details of automation tools used in the process.	2.2 Data extraction and quality 2.3 Quality of evidence
Effect measures	12	Specify for each outcome the effect measure(s) (e.g. risk ratio, mean difference) used in the synthesis or presentation of results.	2.4 Data analysis
Synthesis methods	13a	Describe the processes used to decide which studies were eligible for each synthesis (e.g. tabulating the study intervention characteristics and comparing against the planned groups for each synthesis (item #5)).	2.4 Data analysis Table 1
	13b	Describe any methods required to prepare the data for presentation or synthesis, such as handling of missing summary statistics, or data conversions.	2.4 Data analysis
	13c	Describe any methods used to tabulate or visually display results of individual studies and syntheses.	2.3 Quality of evidence 2.4 Data analysis
	13d	Describe any methods used to synthesize results and provide a rationale for the choice(s). If meta-analysis was performed, describe the model(s), method(s) to identify the presence and extent of statistical heterogeneity, and software package(s) used.	2.4 Data analysis
	13e	Describe any methods used to explore possible causes of heterogeneity among study results (e.g. subgroup analysis, meta-regression).	2.4 Data analysis
	13f	Describe any sensitivity analyses conducted to assess robustness of the synthesized results.	2.4 Data analysis
Reporting bias assessment	14	Describe any methods used to assess risk of bias due to missing results in a synthesis (arising from reporting biases).	2.4 Data analysis
Certainty assessment	15	Describe any methods used to assess certainty (or confidence) in the body of evidence for an outcome.	2.3 Quality of evidence
**RESULTS**			
Study selection	16a	Describe the results of the search and selection process, from the number of records identified in the search to the number of studies included in the review, ideally using a flow diagram.	3.1 Study selection and characteristics; Figure 1
	16b	Cite studies that might appear to meet the inclusion criteria, but which were excluded, and explain why they were excluded.	/
Study characteristics	17	Cite each included study and present its characteristics.	3.1 Study selection and characteristics; Table 1
Risk of bias in studies	18	Present assessments of risk of bias for each included study.	3.1 Study selection and characteristics; Supplementary table 4
Results of individual studies	19	For all outcomes, present, for each study: (a) summary statistics for each group (where appropriate) and (b) an effect estimate and its precision (e.g. confidence/credible interval), ideally using structured tables or plots.	Table 1
Results of	20a	For each synthesis, briefly summarise the characteristics and risk of bias among contributing studies.	3.1 Study selection and

Section and Topic	Item #	Checklist item	Location where item is reported
syntheses			characteristics; Supplementary table 4
	20b	Present results of all statistical syntheses conducted. If meta-analysis was done, present for each the summary estimate and its precision (e.g. confidence/credible interval) and measures of statistical heterogeneity. If comparing groups, describe the direction of the effect.	3. Results
	20c	Present results of all investigations of possible causes of heterogeneity among study results.	Figure 3, Supplementary Figure 5~26
	20d	Present results of all sensitivity analyses conducted to assess the robustness of the synthesized results.	Supplementary Figures 27–34.
Reporting biases	21	Present assessments of risk of bias due to missing results (arising from reporting biases) for each synthesis assessed.	Supplementary Figure 35-42
Certainty of evidence	22	Present assessments of certainty (or confidence) in the body of evidence for each outcome assessed.	3.1 Study selection and characteristics Supplementary table 5
**DISCUSSION**			
Discussion	23a	Provide a general interpretation of the results in the context of other evidence.	4. Discussion
	23b	Discuss any limitations of the evidence included in the review.	4.3 Strengths and limitations
	23c	Discuss any limitations of the review processes used.	4.3 Strengths and limitations
	23d	Discuss implications of the results for practice, policy, and future research.	5 Conclusion
**OTHER INFORMATION**			
Registration and protocol	24a	Provide registration information for the review, including register name and registration number, or state that the review was not registered.	2 Methods
	24b	Indicate where the review protocol can be accessed, or state that a protocol was not prepared.	2 Methods
	24c	Describe and explain any amendments to information provided at registration or in the protocol.	2 Methods
Support	25	Describe sources of financial or non-financial support for the review, and the role of the funders or sponsors in the review.	Funding
Competing interests	26	Declare any competing interests of review authors.	Conflict of interest
Availability of data, code and other materials	27	Report which of the following are publicly available and where they can be found: template data collection forms; data extracted from included studies; data used for all analyses; analytic code; any other materials used in the review.	Data availability statement, Supplementary material

1.2 Supplementary Table 3 Search strategy

Ovid MEDLINE(R) ALL <1946 to August 16, 2023>	
1	Infant, Extremely Premature/
2	(extreme* adj (premature* or preterm*)).ab,ti.
3	(periviable adj (infant* or birth* or deliver* or gestation* or period)).ab,ti.
4	((week* or wk) adj3 (22 or 23 or 24 or 25) adj5 (born or preterm or premature or deliver* or birth)).ab,ti.
5	(gestation* adj3 (22 or 23 or 24 or 25)).ab,ti.
6	Premature Birth/ or Infant, Premature/
7	((preterm or premature or prematurity) adj (infant* or bab* or child* or newborn* or neonat* or new* born*)).ab,ti.
8	(4 or 5) and (6 or 7)
9	1 or 2 or 3 or 8
10	Mortality/ or Survival/ or Death/
11	(mortalit* or surviv* or death* or dying or died or dead or fatalit* or outcome*).ab,ti.
12	10 or 11
13	Cohort Studies/
14	((cohort* or longitudin* or prospective* or retrospective* or follow-up or observational or epidemiol* or long-term) adj3 (stud* or analys* or data)).ab,ti.
15	13 or 14
16	9 and 12 and 15
17	limit 16 to (english language and yr="2000 -Current")

**Embase** <1974 to 2023 August 16>	
1	(extreme* adj (premature* or preterm*)).ab,ti.
2	(periviable adj (infant* or birth* or deliver* or gestation* or period)).ab,ti.
3	((week* or wk) adj3 (22 or 23 or 24 or 25) adj5 (born or preterm or premature or deliver* or birth)).ab,ti.
4	(gestation* adj3 (22 or 23 or 24 or 25)).ab,ti.
5	((preterm or premature or prematurity) adj (infant* or bab* or child* or newborn* or neonat* or new* born*)).ab,ti.
6	Prematurity/
7	(3 or 4) and (5 or 6)
8	**1 or 2 or 7**
9	Mortality/ or Survival/ or Death/
10	(mortalit* or surviv* or death* or dying or died or dead or fatalit*).ab,ti.
11	**9 or 10**
12	Cohort Analysis/
13	((cohort* or longitudin* or prospective* or retrospective* or follow-up or observational or epidemiol* or long-term) adj3 (stud* or analys* or data)).ab,ti.
14	**12 or 13**
15	**8 and 11 and 14**
16	limit 15 to (english language and yr="2000 -Current")
**CENTRAL** - Cochrane Central Register of Controlled Trials <July 2023>	
1	Infant, Extremely Premature/

2	(extreme* adj (premature* or preterm*)).mp.
3	(periviable adj (infant* or birth* or deliver* or gestation* or period)).mp.
4	((week* or wk) adj3 (22 or 23 or 24 or 25) adj5 (born or preterm or premature or deliver* or birth)).mp.
5	(gestation* adj3 (22 or 23 or 24 or 25)).mp.
6	Premature Birth/ or Infant, Premature/
7	((preterm or premature or prematurity) adj (infant* or bab* or child* or newborn* or neonat* or new* born*)).mp.
8	(4 or 5) and (6 or 7)
9	1 or 2 or 3 or 8
10	Mortality/ or Survival/ or Death/
11	(mortalit* or surviv* or death* or dying or died or dead or fatalit*).mp.
12	10 or 11
13	Cohort Studies/
14	((cohort* or longitudin* or prospective* or retrospective* or follow-up or observational or epidemiol* or long-term) adj3 (stud* or analys* or data)).mp.
15	13 or 14
16	9 and 12 and 15
17	limit 16 to (English language and yr="2000 -Current")
**Web of Science**	
1	TS= (extreme* adj (premature* or preterm*))
2	TS= (periviable adj (infant* or birth* or deliver* or gestation* or period))

3	TS= ((week* or wk) near/3 (22 or 23 or 24 or 25) near/5 (born or preterm or premature or deliver* or birth))
4	TS= (gestation* near/3 (22 or 23 or 24 or 25))
5	TS= ((preterm or premature or prematurity) near/1 (infant* or bab* or child* or newborn* or neonat* or newborn*))
6	**#3 or #4**
7	**#5 and #6**
8	**#1 or #2 or #7**
9	TS= (mortalit* or surviv* or death* or dying or died or dead or fatalit* or outcome*)
10	TS= ((cohort* or longitudin* or prospective* or retrospective* or follow-up or observational or epidemiol* or long-term) near/3 (stud* or analys* or data))
11	#8 and #9 and #10
12	limit time 11 2000-01-01 to 2023-08-16
13	**#12 and LA=(English)**

1.3 Supplementary Table 4 Risk bias of included studies

No	Author year	A	B	C	D	E	F	G	H	I	J	Total	Risk of bias
1	Agarwal 2014		★		★	★			★			★★★★	moderate
2	Ancel 2015	★			★	★	★		★	★	★	★★★★★★★	low
3	Anderson 2016	★			★	★			★	★		★★★★★	moderate
4	Aronsson 2023	★	★		★	★			★			★★★★★	moderate
5	Atwell 2018		★		★	★	★		★			★★★★★	moderate
6	Beek 2021		★		★	★			★			★★★★	moderate
7	Beek 2022	★			★	★	★		★	★	★	★★★★★★★	low
8	Bell 2022	★			★	★	★		★	★	★	★★★★★★★	low
9	Berry 2017		★		★	★			★	★		★★★★★	moderate
10	Bode 2008	★			★	★			★	★	★	★★★★★★	moderate
11	Boland 2016	★			★	★			★	★		★★★★★	moderate
12	Bolisetty 2018	★			★	★			★	★	★	★★★★★★	moderate
13	Chang 2018	★				★			★	★	★	★★★★★	moderate
14	Chen 2016	★			★	★			★	★	★	★★★★★★	moderate
15	Costeloe 2012	★			★	★	★		★		★	★★★★★★	moderate
16	Crane 2015				★	★			★			★★★	high
17	Czarny 2021		★		★	★			★	★		★★★★★	moderate
18	Doyle2010	★	★		★	★			★	★		★★★★★★	moderate
19	express group 2009	★		★	★	★	★		★	★	★	★★★★★★★★	low
20	Fajolu 2019		★		★	★			★			★★★★	moderate

No	Author year	A	B	C	D	E	F	G	H	I	J	Total	Risk of bias
21	Field 2008		★		★	★	★		★			★★★★★	moderate
22	Goya 2014				★	★			★	★	★	★★★★★	moderate
23	Gunn 2012		★		★	★			★	★		★★★★★	moderate
24	Humberg 2020	★			★	★	★		★			★★★★★	moderate
25	Ireland 2019		★		★	★			★			★★★★	moderate
26	Ishii 2013		★		★	★			★	★	★	★★★★★★	moderate
27	Kiechl-Kohlendorfer 2019		★		★	★			★			★★★★	moderate
28	Kim2018		★	★	★	★			★	★		★★★★★★	moderate
29	Kulali 2019		★		★	★			★	★		★★★★★	moderate
30	Kutz 2009				★	★			★			★★★	high
31	Kyser 2012		★		★	★			★	★	★	★★★★★★	moderate
32	Lavilla 2022		★		★	★	★		★			★★★★★	moderate
33	Lee 2010	★			★	★			★	★		★★★★★	moderate
34	Malloy 2015	★	★		★	★			★	★	★	★★★★★★★	low
35	Manuck 2016	★			★	★			★		★	★★★★★	moderate
36	Mehler 2016		★		★	★	★		★			★★★★★	moderate
37	Morgan 2021				★	★			★			★★★	high
38	Morgillo 2014	★	★		★	★						★★★★	moderate
39	Musiime 2021		★		★	★			★	★		★★★★★	moderate
40	Nguyen 2012				★	★			★	★		★★★★	moderate
41	Park 2019	★			★	★			★			★★★★	moderate

No	Author year	A	B	C	D	E	F	G	H	I	J	Total	Risk of bias
42	Piriyapokin 2020				★	★			★			★★★	high
43	Puia-Dumitrescu 2020		★		★	★			★			★★★★	moderate
44	Rahman 2015				★	★			★			★★★	high
45	Rattihalli 2010	★			★	★			★	★	★	★★★★★★	moderate
46	Rodrigo 2015	★			★	★			★			★★★★	moderate
47	Schlapbach2012	★			★	★			★	★		★★★★★	moderate
48	Seaton 2013		★		★	★			★			★★★★	moderate
49	Shah 2020	★			★	★	★		★		★	★★★★★★	moderate
50	Sharp 2018				★	★			★	★	★	★★★★★	moderate
51	Shim 2015	★			★	★			★	★		★★★★★	moderate
52	Sinclair 2019		★		★	★			★			★★★★	moderate
53	Smith 2017	★			★	★			★			★★★★	moderate
54	Stensvold 2017		★		★	★			★	★		★★★★★	moderate
55	Stichtenoth 2012	★			★	★			★			★★★★	moderate
56	Stoll 2015	★			★	★	★		★	★	★	★★★★★★★	low
57	Suciu 2017				★	★			★			★★★	high
58	Thomas 2020		★		★	★			★			★★★★	moderate
59	Thompson 2016		★	★	★	★			★			★★★★★	moderate
60	Uccella 2015		★						★	★	★	★★★★	moderate
61	Wang 2011		★		★	★			★			★★★★	moderate
62	Watkins 2020		★		★	★	★		★	★		★★★★★★	moderate

No	Author year	A	B	C	D	E	F	G	H	I	J	Total	Risk of bias
63	Wu 2019		★		★	★			★			★★★★	moderate
64	younge 2016				★	★			★	★	★	★★★★★	moderate
65	younge 2017	★			★	★	★		★	★	★	★★★★★★★	low
66	Zayek 2011		★		★	★			★	★		★★★★★	moderate
67	Zegers 2015	★			★	★			★		★	★★★★★	moderate
68	Zhang 2022		★		★	★			★	★	★	★★★★★★	moderate
69	Zhu 2021		★		★	★	★		★			★★★★★	moderate

Note. **A:** truly representative of the average **B:** somewhat representative of the average **C:** drawn from the same community as the exposed cohort **D:** secure record/structured interview **E:** Demonstration that outcome of interest was not present at start of study **F:** study controls for (select the most important factor) **G:** study controls for any additional factor H: independent blind assessment /record linkage **I:** follow-up long enough **J:** complete follow up - all subjects accounted for /small lost.

1.4 Supplementary Table 5 Summary of findings table

Certainty assessment							№ of patients		Effect		Certainty	Importance
№ of studies	Study design	Risk of bias	Inconsistency	Indirectness	Imprecision	Other considerations	[intervention]	[comparison]	Relative (95% CI)	Absolute (95% CI)		
**22 GA live births**												
22	observational studies	not serious	not serious	not serious	not serious	none			**ES 0.07** (0.05 to 0.10)	**-- per 1,000** (from -- to --)	⨁⨁◯◯ Low	CRITICAL
**23 GA live births**												
32	observational studies	not serious	not serious	not serious	not serious	none			**ES 0.26** (0.22 to 0.31)	**-- per 1,000** (from -- to --)	⨁⨁◯◯ Low	CRITICAL
**24 GA live births**												

Certainty assessment							№ of patients		Effect		Certainty	Importance
№ of studies	Study design	Risk of bias	Inconsistency	Indirectness	Imprecision	Other considerations	[intervention]	[comparison]	Relative (95% CI)	Absolute (95% CI)		
37	observational studies	not serious	not serious	not serious	not serious	none			**ES 0.49** (0.43 to 0.54)	**-- per 1,000** (from -- to --)	⊕⊕◯◯ Low	CRITICAL
**25 GA live births**												
35	observational studies	not serious	not serious	not serious	not serious	none			**ES 0.68** (0.63 to 0.72)	**-- per 1,000** (from -- to --)	⊕⊕◯◯ Low	CRITICAL
**22 GA NICU admissions**												
31	observational studies	not serious	serious[a]	not serious	not serious	none			**ES 0.30** (0.25 to 0.36)	**-- per 1,000** (from -- to --)	⊕◯◯◯ Very low	CRITICAL

Certainty assessment							№ of patients		Effect		Certainty	Importance
№ of studies	Study design	Risk of bias	Inconsistency	Indirectness	Imprecision	Other considerations	[intervention]	[comparison]	Relative (95% CI)	Absolute (95% CI)		
**23 GA NICU admissions**												
50	observational studies	serious[b]	not serious	not serious	not serious	none			**ES 0.44** (0.41 to 0.48)	**-- per 1,000** (from -- to --)	⊕◯◯◯ Very low	CRITICAL
**24 GA NICU admissions**												
52	observational studies	serious[c]	not serious	not serious	not serious	none			**ES 0.61** (0.57 to 0.64)	**-- per 1,000** (from -- to --)	⊕◯◯◯ Very low	CRITICAL
**25 GA NICU admissions**												

Certainty assessment							№ of patients		Effect		Certainty	Importance
№ of studies	Study design	Risk of bias	Inconsistency	Indirectness	Imprecision	Other considerations	[intervention]	[comparison]	Relative (95% CI)	Absolute (95% CI)		
48	observational studies	serious[b]	not serious	not serious	not serious	none			**ES 0.74** (0.70 to 0.77)	**-- per 1,000** (from -- to --)	⊕◯◯◯ Very low	CRITICAL

Note. CI: confidence interval Explanation: a. we rated down because of heterogeneity between studies b. There are 5 studies which are high risk of bias c. There are 4 studies which are high risk of bias

1.5 Supplementary Table 6

Supplementary Table 6 Survival among periviable birth across countries with varied income levels

GA	Of live births			Of infants NICU admissions		
	**overall**	**LMICs**	**HICs**	**overall**	**LMICs**	**HICs**
**22**	7 (5-10)	6 (2-22)	7 (5-10)	30 (25-36)	10 (3-33)	31 (26-37)
**23**	26 (22–31)	13 (6-29)	27 (22-32)	44 (41–48)	29 (12-55)	45 (41-49)
**24**	49 (43–54)	14 (4-38)	52 (47-47)	61 (57–64)	48 (37-59)	62 (59-66)
**25**	68 (63–72)	21 (6-51)	72 (68-75)	74 (70–77)	55 (33-75)	76 (72-79)

1.6 **Supplementary Table** 7

Supplementary Table 7 Survival among periviable birth across different epochs

**GA**	**Of live births**				**Of infants NICU admissions**			
	**overall**	**Epoch 1**	**Epoch 2**	**Epoch 3**	**Overall**	**Epoch 1**	**Epoch 2**	**Epoch 3**
**22**	7 (5-10)	7 (3-13)	7 (4-11)	11 (8-13)	30 (25-36)	25 (14-42)	28 (20-38)	34 (26-44)
**23**	26 (22–31)	21 (12-35)	25 (21-30)	40 (23-61)	44 (41–48)	36 (20-54)	40 (34-47)	53 (48-58)
**24**	49 (43–54)	54 (42-65)	49 (43-56)	39 (22-58)	61 (57–64)	56 (43-69)	59 (54-63)	67 (62-71)
**25**	68 (63–72)	72 (62-81)	69 (64-74)	59 (41-75)	74 (70–77)	74 (63-83)	70 (65-75)	80 (77-83)

1.7 Supplementary Table 8

Supplementary Table 8 the results of meta-regression of infants born at 22 weeks of GA (Live births)

Model	estimate	se	z	P	95%CI
Year	-0.0197	0.0483	-0.4076	0.6835	-0.1144, 0.0750
Sample size	0.0003	0.0003	0.8810	0.3783	-0.0004, 0.0010
Income Middle/low income group	-0.1355	0.9219	-0.1470	0.8831	-1.9425, 1.6715
Country Belgium, France, Italy, Portugal and the UK	-1.2364	1.6263	-0.7603	0.4471	-4.4239, 1.9511
Country China	0.7437	1.1577	0.6425	0.5206	-1.5252, 3.0127
Country France	-1.4035	1.6254	-0.8635	0.3879	-4.5891, 1.7822
Country Germany	2.1349	1.0688	1.9975	0.0458	0.0401, 4.2298
Country Norway	1.9373	0.9896	1.9576	0.0503	-0.0023, 3.8770
Country Spain	0.0929	1.0198	0.0911	0.9274	-1.9058, 2.0917
Country Sweden	0.9841	0.7680	1.2814	0.2001	-0.5212, 2.4894
Country Turkey	0.5255	1.6560	0.3173	0.7510	-2.7202, 3.7712
Country UK	-0.6581	0.8740	-0.7530	0.4514	-2.3712, 1.0549
Country USA	1.2496	0.5471	2.2838	0.0224	0.1772, 2.3220
Rob-low	-1.2093	1.0545	-1.1469	0.2514	-3.2761, 0.8574
Rob-moderate	-1.4815	1.0266	-1.4431	0.1490	-3.4936, 0.5306

1.8 Supplementary Table 9

Supplementary Table 9 the results of meta-regression of infants born at 23 weeks of GA (Live births)

Model	estimate	se	z	P	95%CI
Year	0.0260	0.0293	0.8861	0.3756	-0.0315, 0.0834
Sample size	0.0001	0.0002	0.5776	0.5635	-0.0003, 0.0005
Income Middle/low income group	-0.8799	0.5305	-1.6586	0.0972	-1.9197, 0.1599
Country Belgium, France, Italy, Portugal and the UK	-0.1850	0.5821	-0.3179	0.7506	-1.3259, 0.9559
Country China	-0.1564	0.6145	-0.2546	0.7990	-1.3609, 1.0480
Country France	-3.1383	1.1379	-2.7579	0.0058	-5.3686, -0.9080
Country Germany	0.6459	0.7304	0.8843	0.3765	-0.7857, 2.0775
Country New Zealand	1.6575	0.6256	2.6493	0.0081	0.4313, 2.8837
Country Norway	0.4228	0.6326	0.6683	0.5039	-0.8171, 1.6626
Country Spain	-0.4108	0.4612	-0.8906	0.3731	-1.3147, 0.4932
Country Sweden	1.5001	0.4430	3.3861	0.0007	0.6318, 2.3684
Country Switzerland	-1.0586	0.5070	-2.0879	0.0368	-2.0524, -0.0649
Country Turkey	-1.1733	0.8024	-1.4622	0.1437	-2.7459, 0.3994
Country UK	-0.0354	0.3613	-0.0979	0.9221	-0.7434, 0.6727
Country USA	0.8442	0.2879	2.9328	0.0034	0.2800, 1.4084
Rob-low	0.0453	0.8150	0.0555	0.9557	-1.5521, 1.6426
Rob-moderate	-0.4486	0.7746	-0.5792	0.5625	-1.9668, 1.0696

1.9 **Supplementary Table 10**

Supplementary Table 10 the results of meta-regression of infants born at 24 weeks of GA (Live births)

Model	estimate	se	z	P	95%CI
Year	-0.0296	0.0268	-1.1044	0.2694	-0.0820, 0.0229
Sample size	0.0001	0.0001	0.5486	0.5833	-0.0002, 0.0003
Income Middle/low income group	-1.6779	0.3847	-4.3620	<.0001	-2.4318 -0.9240
Country Austria	0.8281	0.3989	2.0762	0.0379	0.0463, 1.6098
Country Belgium, France, Italy, Portugal and the UK	-0.3151	0.3948	-0.7980	0.4248	-1.0889, 0.4587
Country China	-1.6946	0.3352	-5.0551	<.0001	-2.3516, -1.0376
Country France	-0.9698	0.4084	-2.3746	0.0176	-1.7703, -0.1694
Country Germany	0.3279	0.5425	0.6044	0.5456	-0.7354, 1.3912
Country New Zealand	0.2157	0.4584	0.4705	0.6380	-0.6828,1.1142
Country Nigeria	-3.4788	1.4886	-2.3369	0.0194	-6.3964, -0.5611
Country Norway	0.0724	0.4544	0.1593	0.8734	-0.8182, 0.9630
Country Spain	-0.6479	0.3116	-2.0793	0.0376	-1.2586, -0.0372
Country Sweden	0.7102	0.3373	2.1055	0.0352	0.0491, 1.3713
Country Switzerland	-0.7178	0.2758	-2.6028	0.0092	-1.2583, -0.1773
Country the Netherlands	-1.0435	0.2599	-4.0144	<.0001	-1.5530, -0.5340
Country Turkey	-1.0559	0.4773	-2.2120	0.0270	-1.9914, -0.1203
Country UK	-0.7192	0.2685	-2.6790	0.0074	-1.2454, -0.1930

Country USA	0.4470	0.2087	2.1423	0.0322	0.0380, 0.8560
Rob-low	-0.3208	0.7257	-0.4421	0.6584	-1.7431, 1.1015
Rob-moderate	-0.6360	0.6956	-0.9143	0.3606	-1.9994, 0.7274

1.10 Supplementary Table 11

Supplementary Table 11 the results of meta-regression of infants born at 25 weeks of GA (Live births)

Model	estimate	se	z	P	95%CI
Year	-0.0424	0.0250	-1.6987	0.0894	-0.0913, 0.0065
Sample size	0.0001	0.0001	0.4953	0.6204	-0.0002, 0.0003
Income Middle/low income group	-2.0029	0.3243	-6.1765	<.0001	-2.6384, -1.3673
Country Austria	0.8593	0.3593	2.3914	0.0168	0.1550, 1.5636
Country Belgium, France, Italy, Portugal and the UK	-0.3186	0.3454	-0.9225	0.3563	-0.9956, 0.3583
Country China	-1.8679	0.2765	-6.7563	<.0001	-2.4098, -1.3261
Country China(Taiwan)	0.4323	0.5636	0.7669	0.4431	-0.6724, 1.5370
Country France	-0.4993	0.3450	-1.4473	0.1478	-1.1754, 0.1768
Country Nigeria	-4.1628	1.0689	-3.8944	<.0001	-6.2579, -2.0678
Country Norway	0.8126	0.4620	1.7590	0.0786	-0.0928, 1.7181
Country Spain	-0.4978	0.2736	-1.8193	0.0689	-1.0341, 0.0385
Country Sweden	0.8784	0.3184	2.7586	0.0058	0.2543, 1.5025
Country Switzerland	-0.5036	0.2471	-2.0379	0.0416	-0.9880, -0.0193

Country the Netherlands	-0.4560	0.2331	-1.9563	0.0504	-0.9128, 0.0009
Country Turkey	-1.1265	0.4137	-2.7228	0.0065	-1.9374, -0.3156
Country UK	-0.4472	0.2402	-1.8621	0.0626	-0.9179,0.0235
Country USA	0.5143	0.1959	2.6247	0.0087	0.1302,0.8983

1.11 Supplementary Table 12

Supplementary Table 12 the results of meta-regression of infants born at 22 weeks of GA (NICU admissions)

Model	estimate	se	z	P	95%CI
Year	0.0083	0.0340	0.2425	0.8084	-0.0585, 0.0750
Sample size	0.0000	0.0003	0.0199	0.9841	-0.0005, 0.0006
Income levels	-1.4106	0.8553	-1.6492	0.0991	-3.0870, 0.2658
Country China	-1.9122	1.1593	-1.6495	0.0990	-4.1844, 0.3599
Country China(Taiwan)	-2.1273	0.9170	-2.3198	0.0204	-3.9247, -0.3300
Country France	-1.4014	1.7312	-0.8095	0.4182	-4.7946, 1.9918
Country Germany	0.3828	0.7011	0.5460	0.5851	-0.9914, 1.7570
Country Japan	-0.3594	0.8088	-0.4443	0.6568	-1.9446, 1.2259
Country Korea	-1.2348	0.7683	-1.6072	0.1080	-2.7407, 0.2710
Country Norway	0.5445	1.1327	0.4807	0.6307	-1.6755, 2.7645
Country Spain	-2.1144	1.0181	-2.0769	0.0378	-4.1097, -0.1190
Country Sweden	-0.6951	0.8258	-0.8418	0.3999	-2.3135, 0.9233

Country Turkey	-2.1899	1.6670	-1.3136	0.1890	-5.4572, 1.0775
Country UK	-1.2327	0.8303	-1.4846	0.1376	-2.8600, 0.3947
Country USA	-0.6386	0.6549	-0.9752	0.3295	-1.9222, 0.6449
Rob-low	-1.3889	1.0120	-1.3724	0.1699	-3.3723, 0.5946
Rob-moderate	-0.9850	0.9626	-1.0233	0.3062	-2.8717, 0.9016

1.12 **Supplementary Table 13**

Supplementary Table 13 the results of meta-regression of infants born at 23 weeks of GA (NICU admissions)

Model	estimate	se	z	P	95%CI
Year	0.0356	0.0201	1.7728	0.0763	-0.0038, 0.0749
Sample size	0.0000	0.0001	0.5184	0.6041	-0.0001, 0.0002
Income Middle/low income group	-0.6929	0.3859	-1.7956	0.0726	-1.4493, 0.0634
Country Canada	0.0229	0.3069	0.0747	0.9404	-0.5787, 0.6245
Country China	-0.4832	0.5434	-0.8892	0.3739	-1.5482, 0.5819
Country China (Taiwan)	-0.5660	0.4145	-1.3657	0.1720	-1.3784, 0.2463
Country France	-1.2751	1.0020	-1.2725	0.2032	-3.2389, 0.6888
Country Germany	0.6225	0.3117	1.9974	0.0458	0.0117, 1.2334
Country Italy	-1.4182	0.6338	-2.2377	0.0252	-2.6603, -0.1760
Country Japan	0.7151	0.4482	1.5956	0.1106	-0.1633, 1.5936
Country Korea	0.3022	0.3592	0.8414	0.4001	-0.4017, 1.0062

Country New Zealand	0.7791	0.5549	1.4040	0.1603	-0.3085, 1.8666
Country Norway	-0.3965	0.5549	-0.7146	0.4749	-1.4841, 0.6911
Country Oman	-1.4182	0.9917	-1.4300	0.1527	-3.3619, 0.5255
Country Singapore	0.2746	0.5920	0.4640	0.6427	-0.8856, 1.4349
Country South Africa	-1.4182	1.6073	-0.8823	0.3776	-4.5685, 1.7321
Country South Korea	-0.2967	0.4514	-0.6572	0.5111	-1.1814, 0.5881
Country Spain	-1.1419	0.3907	-2.9229	0.0035	-1.9075, -0.3762
Country Sweden	0.7989	0.3800	2.1024	0.0355	0.0541, 1.5436
Country Switzerland	-0.2951	0.4773	-0.6183	0.5364	-1.2306, 0.6403
Country Thailand	0.6433	0.8068	0.7972	0.4253	-0.9381, 2.2246
Country Turkey	-1.9737	0.7085	-2.7857	0.0053	-3.3623-0.5851
Country UK	-0.7609	0.2961	-2.5694	0.0102	-1.3413, -0.1805
Country USA	0.1681	0.2269	0.7406	0.4589	-0.2767, 0.6129
Rob-low	-0.1382	0.4201	-0.3290	0.7422	-0.9616, 0.6852
Rob-moderate	-0.2546	0.3305	-0.7702	0.4412	-0.9024, 0.3932

1.13 **Supplementary Table 14**

Supplementary Table 14 the results of meta-regression of infants born at 24 weeks of GA (NICU admissions)

Model	estimate	se	z	P	95%CI
Year	0.0133	0.0188	0.7100	0.4777	-0.0235, 0.0501
Sample size	0.0000	0.0001	0.2117	0.8324	-0.0002, 0.0002
Income Middle/low income group	-0.5937	0.2374	-2.5004	0.0124	-1.0591, -0.1283
Country Canada	0.1926	0.2946	0.6538	0.5133	-0.3849, 0.7701
Country China	-0.6694	0.2981	-2.2458	0.0247	-1.2536, -0.0852
Country China(taiwan)	-0.5947	0.3377	-1.7610	0.0782	-1.2566, 0.0672
Country France	-0.6304	0.4228	-1.4910	0.1360	-1.4590, 0.1983
Country Germany	0.3158	0.3005	1.0512	0.2932	-0.2730, 0.9047
Country Italy	-1.1604	0.4831	-2.4021	0.0163	-2.1072, -0.2136
Country Japan	0.5310	0.4005	1.3259	0.1849	-0.2539, 1.3159
Country Korea	-0.2940	0.4122	-0.7132	0.4757	-1.1020, 0.5140
Country New Zealand	-0.2354	0.4632	-0.5083	0.6113	-1.1433, 0.6725
Country Norway	-0.4136	0.3866	-1.0697	0.2848	-1.1714, 0.3442
Country Oman	-1.3766	0.6658	-2.0675	0.0387	-2.6816, -0.0716
Country Singapore	0.0967	0.4330	0.2233	0.8233	-0.7520, 0.9454
Country South Africa	-1.6643	0.7757	-2.1456	0.0319	-3.1846, -0.1440

Country South Korea	-0.6397	0.3927	-1.6289	0.1033	-1.4095, 0.1300
Country Spain	-0.9594	0.3132	-3.0634	0.0022	-1.5732, -0.3456
Country Sweden	0.4322	0.3423	1.2629	0.2066	-0.2386, 1.1031
Country Switzerland	-0.4712	0.3288	-1.4330	0.1519	-1.1156, 0.1733
Country Thailand	-0.5011	0.5715	-0.8768	0.3806	-1.6213, 0.6190
Country the Netherlands	-0.6521	0.2586	-2.5214	0.0117	-1.1590, -0.1452
Country Turkey	-1.4662	0.4841	-3.0285	0.0025	-2.4151, -0.5173
Country USA	0.2312	0.1999	1.1562	0.2476	-0.1607, 0.6230
Country UK	-0.7852	0.2328	-3.3725	0.0007	-1.2415, -0.3289
Rob-low	0.0609	0.4085	0.1491	0.8815	-0.7397, 0.8615
Rob-moderate	0.0686	0.3448	0.1988	0.8424	-0.6072, 0.7443

1.14 **Supplementary Table 15**

Supplementary Table 15 the results of meta-regression of infants born at 25 weeks of GA (NICU admissions)

Model	estimate	se	z	P	95%CI
Year	0.0049	0.0224	0.2171	0.8281	-0.0391, 0.0488
Sample size	0.0001	0.0002	0.3748	0.7078	-0.0003, 0.0004
Income Middle/low income group	-0.8955	0.2779	-3.2227	0.0013	-1.4401, -0.3509
Country Canada	0.6112	0.3357	1.8204	0.0687	-0.0469, 1.2692
Country China	-0.6317	0.3156	-2.0018	0.0453	-1.2503, -0.0132
Country China(Taiwan)	-0.3753	0.4483	-0.8371	0.4025	-1.2539, 0.5034
Country France	-0.4939	0.4495	-1.0988	0.2718	-1.3748, 0.3870
Country Germany	0.3914	0.3177	1.2319	0.2180	-0.2313, 1.0140
Country Italy	-0.0149	0.5557	-0.0268	0.9786	-1.1040, 1.0742
Country Japan	0.6664	0.4541	1.4677	0.1422	-0.2235, 1.5564
Country Korea	-0.2185	0.4559	-0.4794	0.6317	-1.1121, 0.6750
Country Nigeria	-4.3786	1.1062	-3.9583	<.0001	-6.5467, -2.2105
Country Norway	0.4322	0.4253	1.0163	0.3095	-0.4013, 1.2656
Country Oman	-0.1177	0.5994	-0.1963	0.8443	-1.2924, 1.0571
Country Romania	-2.1307	0.4865	-4.3801	<.0001	-3.0842, -1.1773
Country Singapore	0.4609	0.4917	0.9374	0.3486	-0.5028, 1.4247

Country South Africa	-1.4882	0.5705	-2.6085	0.0091	-2.6064, -0.3700
Country Spain	-0.4970	0.3459	-1.4367	0.1508	-1.1750, 0.1810
Country Sweden	0.7989	0.3849	2.0754	0.0380	0.0444, 1.5534
Country Switzerland	-0.1512	0.5421	-0.2789	0.7803	-1.2136, 0.9112
Country Thailand	1.0275	0.6833	1.5036	0.1327	-0.3118, 2.3667
Country the Netherlands	-0.2201	0.2897	-0.7597	0.4474	-0.7879, 0.3477
Country Turkey	-1.2527	0.5049	-2.4811	0.0131	-2.2422, -0.2631
Country UK	-0.4672	0.2690	-1.7367	0.0824	-0.9944, 0.0601
Country UK-England	0.3343	0.5847	0.5717	0.5675	-0.8118, 1.4804
Country USA	0.6047	0.2505	2.4134	0.0158	0.1136, 1.0957
Rob-low	0.3596	0.4359	0.8250	0.4094	-0.4948, 1.2141
Rob-moderate	0.2417	0.3334	0.7251	0.4684	-0.4116, 0.8951

1.15 **Supplementary Table 16**

Supplementary Table 16 Linear regression test of funnel plot asymmetry

Group	t	P
22 GA (Live births)	-2.63	0.0161
23 GA (Live births)	-1.48	0.1490
24 GA (Live births)	-1.39	0.1744
25 GA (Live births)	-0.5	0.6182
22 GA (NICU admissions)	-0.53	0.6023
23 GA (NICU admissions)	-1.39	0.1703
24 GA (NICU admissions)	-0.19	0.853
25 GA (NICU admissions)	0.4	0.6920

2 Supplementary Figures

1.16 Supplementary Figure 5

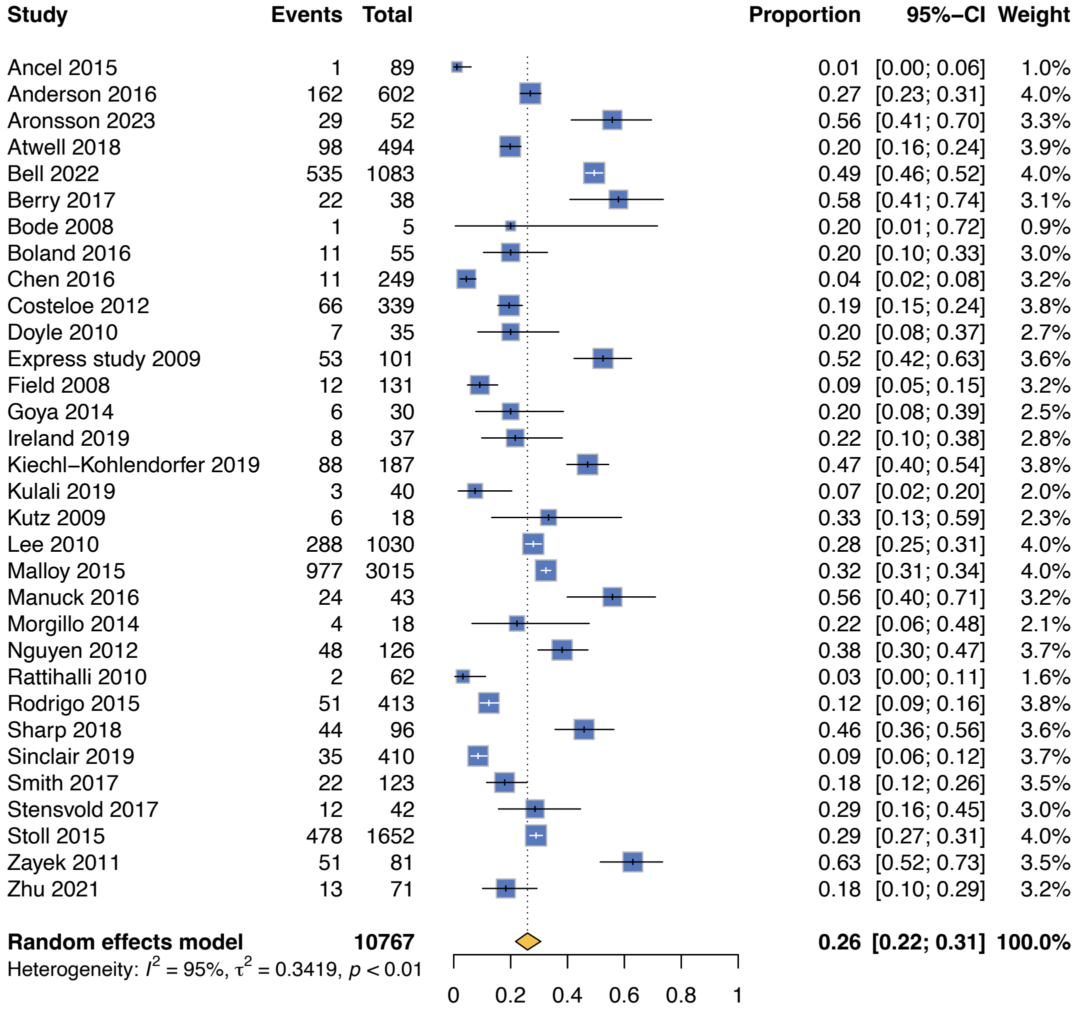

Supplementary Figure 5. Survival rates among periviable infants born at 23 weeks of GA (Live births)

1.17 Supplementary Figure 6

Study	Events	Total	Proportion	95%-CI	Weight
Ancel 2015	58	186	0.31	[0.25; 0.38]	2.9%
Anderson 2016	458	766	0.60	[0.56; 0.63]	3.0%
Aronsson 2023	41	53	0.77	[0.64; 0.88]	2.4%
Atwell 2018	361	696	0.52	[0.48; 0.56]	3.0%
Beek 2021	240	697	0.34	[0.31; 0.38]	3.0%
Beek 2022	114	271	0.42	[0.36; 0.48]	3.0%
Bell 2022	972	1391	0.70	[0.67; 0.72]	3.1%
Berry 2017	36	60	0.60	[0.47; 0.72]	2.6%
Bode 2008	11	12	0.92	[0.62; 1.00]	1.0%
Boland 2016	41	84	0.49	[0.38; 0.60]	2.7%
Chen 2016	150	442	0.34	[0.30; 0.39]	3.0%
Costeloe 2012	178	442	0.40	[0.36; 0.45]	3.0%
Doyle 2010	22	43	0.51	[0.35; 0.67]	2.5%
Express study 2009	96	144	0.67	[0.58; 0.74]	2.9%
Fajolu 2019	0	13	0.00	[0.00; 0.25]	0.5%
Field 2008	82	227	0.36	[0.30; 0.43]	2.9%
Goya 2014	30	65	0.46	[0.34; 0.59]	2.7%
Ireland 2019	48	76	0.63	[0.51; 0.74]	2.7%
Kiechl–Kohlendorfer 2019	218	297	0.73	[0.68; 0.78]	3.0%
Kulali 2019	16	55	0.29	[0.18; 0.43]	2.5%
Kutz 2009	17	27	0.63	[0.42; 0.81]	2.2%
Lee 2010	888	1480	0.60	[0.57; 0.63]	3.1%
Malloy 2015	2248	3846	0.58	[0.57; 0.60]	3.1%
Manuck 2016	78	114	0.68	[0.59; 0.77]	2.8%
Morgillo 2014	22	40	0.55	[0.38; 0.71]	2.4%
Rattihalli 2010	31	93	0.33	[0.24; 0.44]	2.7%
Rodrigo 2015	396	1176	0.34	[0.31; 0.36]	3.1%
Schlapbach 2012	62	210	0.30	[0.23; 0.36]	2.9%
Sharp 2018	112	146	0.77	[0.69; 0.83]	2.8%
Sinclair 2019	218	579	0.38	[0.34; 0.42]	3.0%
Smith 2017	134	287	0.47	[0.41; 0.53]	3.0%
Stensvold 2017	35	62	0.56	[0.43; 0.69]	2.6%
Stoll 2015	1526	2618	0.58	[0.56; 0.60]	3.1%
Zayek 2011	82	101	0.81	[0.72; 0.88]	2.6%
Zegers 2015	131	782	0.17	[0.14; 0.20]	3.0%
Zhang 2022	6	215	0.03	[0.01; 0.06]	2.2%
Zhu 2021	144	408	0.35	[0.31; 0.40]	3.0%
**Random effects model**		**18204**	**0.49**	**[0.43; 0.54]**	**100.0%**

Heterogeneity: $I^2 = 97\%$, $\tau^2 = 0.3709$, $p < 0.01$

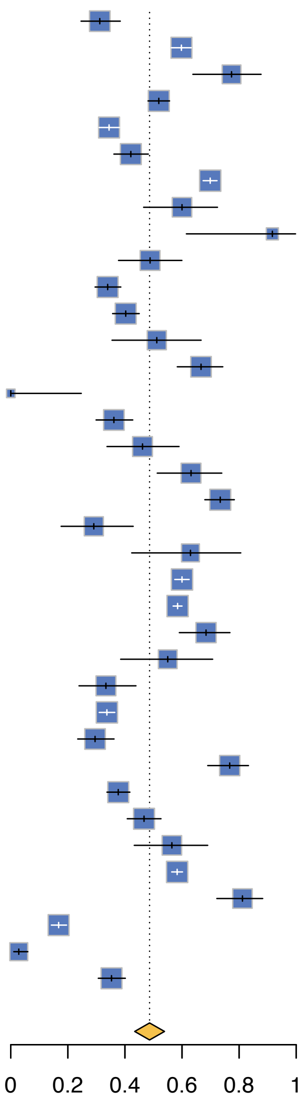

Supplementary Figure 6. Survival rates among periviable infants born at 24 weeks of GA (Live births)

1.18 Supplementary Figure 7

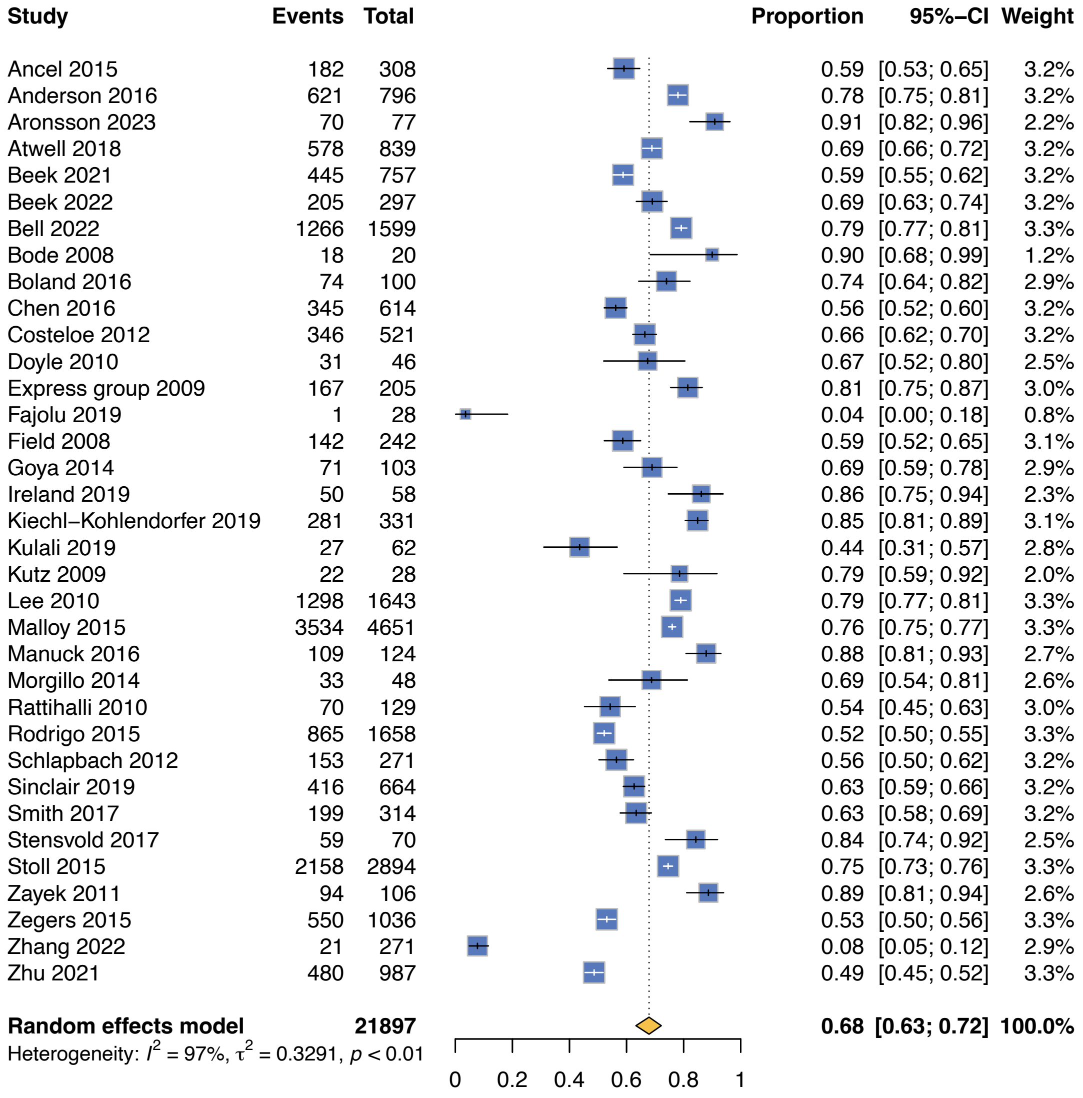

Supplementary Figure 7. Survival rates among periviable infants born at 25 weeks of GA (Live births)

1.19 Supplementary Figure 8

Study	Events	Total	Proportion	95%–CI	Weight
Agarwal 2014	12	23	0.52	[0.31; 0.73]	1.6%
Ancel 2015	1	7	0.14	[0.00; 0.58]	0.6%
Aronsson 2023	29	45	0.64	[0.49; 0.78]	2.0%
Atwell 2018	98	187	0.52	[0.45; 0.60]	2.7%
Bell 2022	535	958	0.56	[0.53; 0.59]	2.9%
Berry 2017	22	34	0.65	[0.46; 0.80]	1.8%
Bolisetty 2019	14	50	0.28	[0.16; 0.42]	2.0%
Chang 2018	27	108	0.25	[0.17; 0.34]	2.4%
Chen 2016	11	36	0.31	[0.16; 0.48]	1.8%
Costeloe 2012	66	217	0.30	[0.24; 0.37]	2.7%
Crane 2015	25	69	0.36	[0.25; 0.49]	2.3%
Czarny 2021	3990	7708	0.52	[0.51; 0.53]	2.9%
Express group 2009	53	81	0.65	[0.54; 0.76]	2.3%
Field 2008	12	65	0.18	[0.10; 0.30]	2.0%
Goya 2014	6	18	0.33	[0.13; 0.59]	1.4%
Gunn 2012	5	11	0.45	[0.17; 0.77]	1.1%
Humberg 2020	355	576	0.62	[0.58; 0.66]	2.8%
Ireland 2019	8	18	0.44	[0.22; 0.69]	1.4%
Ishii 2013	154	245	0.63	[0.56; 0.69]	2.7%
Kim 2018	62	96	0.65	[0.54; 0.74]	2.4%
Kulali 2019	3	33	0.09	[0.02; 0.24]	1.1%
Kutz 2009	6	12	0.50	[0.21; 0.79]	1.2%
Kyser 2012	22	38	0.58	[0.41; 0.74]	1.9%
Lavilla 2022	25	63	0.40	[0.28; 0.53]	2.2%
Lee 2010	286	664	0.43	[0.39; 0.47]	2.9%
Mehler 2016	41	58	0.71	[0.57; 0.82]	2.1%
Morgan 2021	18	22	0.82	[0.60; 0.95]	1.2%
Morgillo 2014	4	7	0.57	[0.18; 0.90]	0.8%
Musiime 2021	0	2	0.00	[0.00; 0.84]	0.2%
Park 2019	79	208	0.38	[0.31; 0.45]	2.7%
Piriyapokin 2020	5	8	0.62	[0.24; 0.91]	0.9%
Puia–Dumitrescu 2020	1116	2690	0.41	[0.40; 0.43]	2.9%
Rahman 2015	1	8	0.12	[0.00; 0.53]	0.6%
Rattihalli 2010	2	29	0.07	[0.01; 0.23]	0.9%
Rodrigo 2015	51	316	0.16	[0.12; 0.21]	2.6%
Seaton 2013	16	80	0.20	[0.12; 0.30]	2.1%
Shah 2020	339	679	0.50	[0.46; 0.54]	2.9%
Sharp 2018	44	78	0.56	[0.45; 0.68]	2.3%
Shim 2015	44	107	0.41	[0.32; 0.51]	2.5%
Sinclair 2019	35	110	0.32	[0.23; 0.41]	2.4%
Stensvold 2017	12	34	0.35	[0.20; 0.54]	1.8%
Stichtenoth 2012	38	76	0.50	[0.38; 0.62]	2.3%
Thomas 2020	48	97	0.49	[0.39; 0.60]	2.4%
Thompson 2016	66	121	0.55	[0.45; 0.64]	2.5%
Uccella 2015	5	32	0.16	[0.05; 0.33]	1.4%
Watkins 2020	41	50	0.82	[0.69; 0.91]	1.8%
Younge 2017	303	1025	0.30	[0.27; 0.32]	2.9%
Younge 2016	27	66	0.41	[0.29; 0.54]	2.3%
Zayek 2011	51	75	0.68	[0.56; 0.78]	2.3%
Zhu 2021	13	39	0.33	[0.19; 0.50]	1.9%
**Random effects model**		**17379**	**0.44**	**[0.41; 0.48]**	**100.0%**

Heterogeneity: $I^2 = 93\%$, $\tau^2 = 0.2026$, $p < 0.01$

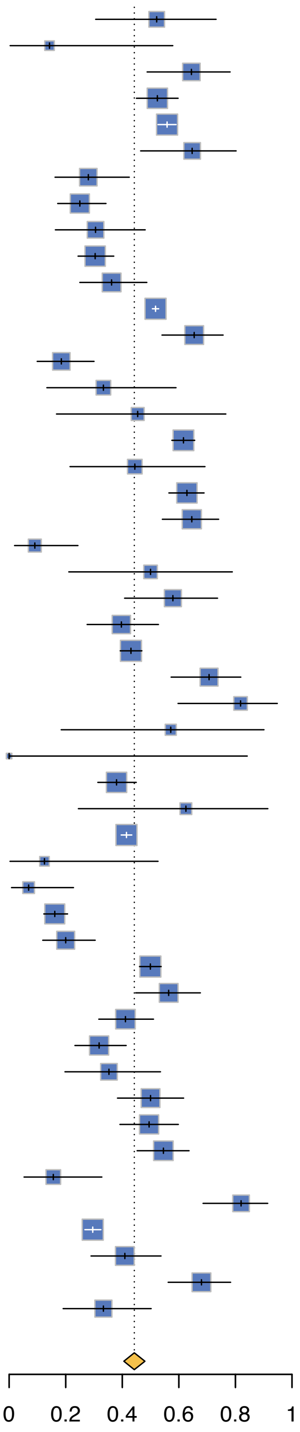

Supplementary Figure 8. Survival rates among periviable infants born at 23 weeks of GA (NICU admissions)

1.20 Supplementary Figure 9

Study	Events	Total	Proportion	95%–CI	Weight
Agarwal 2014	72	105	0.69	[0.59; 0.77]	2.0%
Ancel 2015	58	113	0.51	[0.42; 0.61]	2.1%
Aronsson 2023	41	52	0.79	[0.65; 0.89]	1.6%
Atwell 2018	361	560	0.64	[0.60; 0.68]	2.3%
Beek 2021	240	480	0.50	[0.45; 0.55]	2.3%
Beek 2022	114	212	0.54	[0.47; 0.61]	2.2%
Bell 2022	972	1362	0.71	[0.69; 0.74]	2.3%
Berry 2017	36	59	0.61	[0.47; 0.73]	1.8%
Bolisetty 2019	130	224	0.58	[0.51; 0.65]	2.2%
Chang 2018	117	250	0.47	[0.40; 0.53]	2.2%
Chen 2016	150	289	0.52	[0.46; 0.58]	2.2%
Costeloe 2012	178	381	0.47	[0.42; 0.52]	2.3%
Express group 2009	96	132	0.73	[0.64; 0.80]	2.0%
Field 2008	82	198	0.41	[0.34; 0.49]	2.2%
Goya 2014	30	55	0.55	[0.41; 0.68]	1.8%
Gunn 2012	51	90	0.57	[0.46; 0.67]	2.0%
Humberg 2020	868	1165	0.75	[0.72; 0.77]	2.3%
Ireland 2019	48	68	0.71	[0.58; 0.81]	1.8%
Ishii 2013	256	332	0.77	[0.72; 0.82]	2.2%
Kulali 2019	16	51	0.31	[0.19; 0.46]	1.7%
Kutz 2009	17	27	0.63	[0.42; 0.81]	1.4%
Kyser 2012	76	87	0.87	[0.79; 0.94]	1.6%
Lavilla 2022	57	84	0.68	[0.57; 0.78]	1.9%
Lee 2010	891	1392	0.64	[0.61; 0.67]	2.3%
Morgan 2021	29	38	0.76	[0.60; 0.89]	1.5%
Morgillo 2014	22	36	0.61	[0.43; 0.77]	1.6%
Musiime 2021	3	11	0.27	[0.06; 0.61]	0.8%
Park 2019	187	366	0.51	[0.46; 0.56]	2.3%
Piriyapokin 2020	12	22	0.55	[0.32; 0.76]	1.3%
Puia–Dumitrescu 2020	2914	4559	0.64	[0.63; 0.65]	2.4%
Rahman 2015	5	15	0.33	[0.12; 0.62]	1.0%
Rattihalli 2010	31	78	0.40	[0.29; 0.51]	1.9%
Rodrigo 2015	396	1097	0.36	[0.33; 0.39]	2.3%
Seaton 2013	66	151	0.44	[0.36; 0.52]	2.1%
Shah 2020	1040	1504	0.69	[0.67; 0.71]	2.3%
Sharp 2018	112	139	0.81	[0.73; 0.87]	2.0%
Shim 2015	93	156	0.60	[0.51; 0.67]	2.1%
Sinclair 2019	218	414	0.53	[0.48; 0.58]	2.3%
Stensvold 2017	35	60	0.58	[0.45; 0.71]	1.8%
Stichtenoth 2012	122	171	0.71	[0.64; 0.78]	2.1%
Thomas 2020	246	341	0.72	[0.67; 0.77]	2.2%
Thompson 2016	149	185	0.81	[0.74; 0.86]	2.1%
Uccella 2015	18	47	0.38	[0.25; 0.54]	1.7%
Wang 2011	8	15	0.53	[0.27; 0.79]	1.1%
Watkins 2020	70	79	0.89	[0.79; 0.95]	1.5%
Wu 2019	36	105	0.34	[0.25; 0.44]	2.0%
Younge 2016	80	118	0.68	[0.59; 0.76]	2.0%
Younge 2017	1040	1994	0.52	[0.50; 0.54]	2.4%
Zayek 2011	82	85	0.96	[0.90; 0.99]	1.0%
Zegers 2015	131	269	0.49	[0.43; 0.55]	2.2%
Zhang 2022	6	9	0.67	[0.30; 0.93]	0.8%
Zhu 2021	144	238	0.61	[0.54; 0.67]	2.2%
**Random effects model**		**20070**	**0.61**	**[0.57; 0.64]**	**100.0%**

Heterogeneity: $I^2 = 95\%$, $\tau^2 = 0.2328$, $p < 0.01$

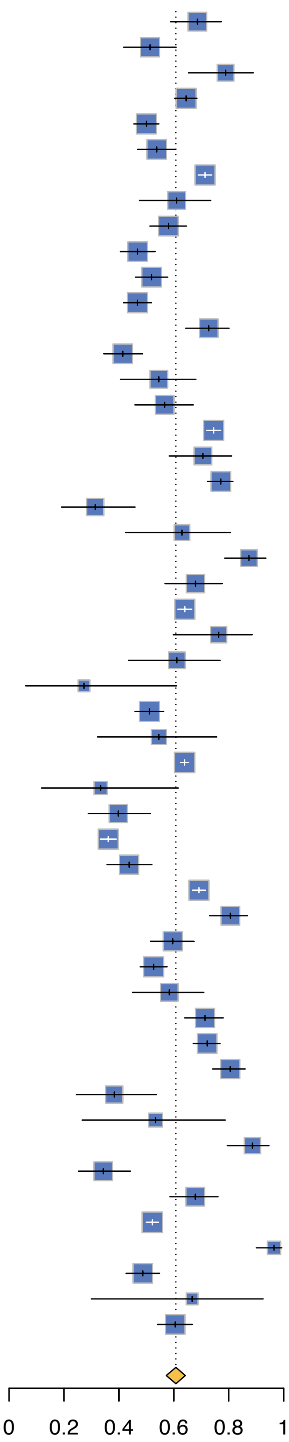

Supplementary Figure 9. Survival rates among periviable infants born at 24 weeks of GA (NICU admissions)

1.21 Supplementary Figure 10

Study	Events	Total	Proportion	95%-CI	Weight
Agarwal 2014	103	125	0.82	[0.75; 0.89]	2.1%
Ancel 2015	182	283	0.64	[0.58; 0.70]	2.4%
Aronsson 2023	70	77	0.91	[0.82; 0.96]	1.7%
Atwell 2018	578	754	0.77	[0.73; 0.80]	2.4%
Beek 2021	445	625	0.71	[0.67; 0.75]	2.4%
Beek 2022	205	273	0.75	[0.70; 0.80]	2.3%
Bell 2022	1266	1584	0.80	[0.78; 0.82]	2.4%
Bolisetty 2019	257	344	0.75	[0.70; 0.79]	2.4%
Chang 2018	211	315	0.67	[0.61; 0.72]	2.4%
Chen 2016	345	565	0.61	[0.57; 0.65]	2.4%
Costeloe 2012	346	498	0.69	[0.65; 0.73]	2.4%
Express group 2009	167	200	0.83	[0.78; 0.88]	2.2%
Fajolu 2019	1	28	0.04	[0.00; 0.18]	0.6%
Field 2008	142	225	0.63	[0.56; 0.69]	2.3%
Goya 2014	71	95	0.75	[0.65; 0.83]	2.1%
Gunn 2012	75	101	0.74	[0.65; 0.82]	2.1%
Humberg 2020	1012	1217	0.83	[0.81; 0.85]	2.4%
Ireland 2019	50	56	0.89	[0.78; 0.96]	1.6%
Ishii 2013	345	405	0.85	[0.81; 0.89]	2.3%
Kulali 2019	27	59	0.46	[0.33; 0.59]	2.1%
Kutz 2009	22	27	0.81	[0.62; 0.94]	1.4%
Kyser 2012	88	103	0.85	[0.77; 0.92]	2.0%
Lavilla 2022	70	84	0.83	[0.74; 0.91]	2.0%
Lee 2010	1303	1609	0.81	[0.79; 0.83]	2.4%
Morgan 2021	33	41	0.80	[0.65; 0.91]	1.7%
Morgillo 2014	33	46	0.72	[0.57; 0.84]	1.9%
Musiime 2021	12	30	0.40	[0.23; 0.59]	1.7%
Piriyapokin 2020	33	37	0.89	[0.75; 0.97]	1.3%
Rahman 2015	21	29	0.72	[0.53; 0.87]	1.6%
Rattihalli 2010	70	119	0.59	[0.49; 0.68]	2.2%
Rodrigo 2015	865	1566	0.55	[0.53; 0.58]	2.5%
Seaton 2013	137	204	0.67	[0.60; 0.74]	2.3%
Shah 2020	1681	2067	0.81	[0.80; 0.83]	2.5%
Shim 2015	159	226	0.70	[0.64; 0.76]	2.3%
Sinclair 2019	416	597	0.70	[0.66; 0.73]	2.4%
Stensvold 2017	59	69	0.86	[0.75; 0.93]	1.8%
Stichtenoth 2012	124	157	0.79	[0.72; 0.85]	2.2%
Suciu 2017	27	104	0.26	[0.18; 0.35]	2.2%
Thomas 2020	383	438	0.87	[0.84; 0.90]	2.3%
Thompson 2016	177	220	0.80	[0.75; 0.85]	2.3%
Uccella 2015	32	43	0.74	[0.59; 0.86]	1.8%
Wang 2011	23	30	0.77	[0.58; 0.90]	1.6%
Watkins 2020	89	99	0.90	[0.82; 0.95]	1.9%
Wu 2019	89	263	0.34	[0.28; 0.40]	2.4%
Zayek 2011	94	106	0.89	[0.81; 0.94]	1.9%
Zegers 2015	550	852	0.65	[0.61; 0.68]	2.4%
Zhang 2022	21	25	0.84	[0.64; 0.95]	1.3%
Zhu 2021	480	644	0.75	[0.71; 0.78]	2.4%
**Random effects model**		**17664**	**0.74**	**[0.70; 0.77]**	**100.0%**

Heterogeneity: $I^2 = 96\%$, $\tau^2 = 0.3327$, $p < 0.01$

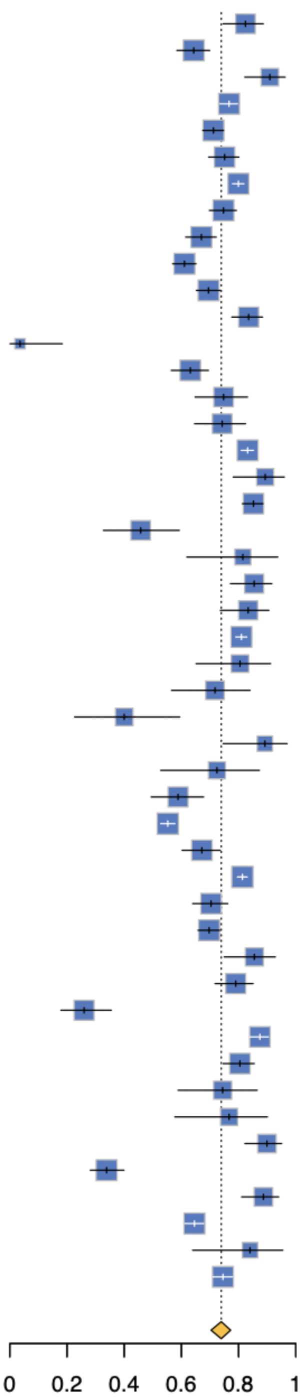

Supplementary Figure 10. Survival rates among periviable infants born at 25 weeks of GA (NICU admissions)

1.22 Supplementary Figure 11

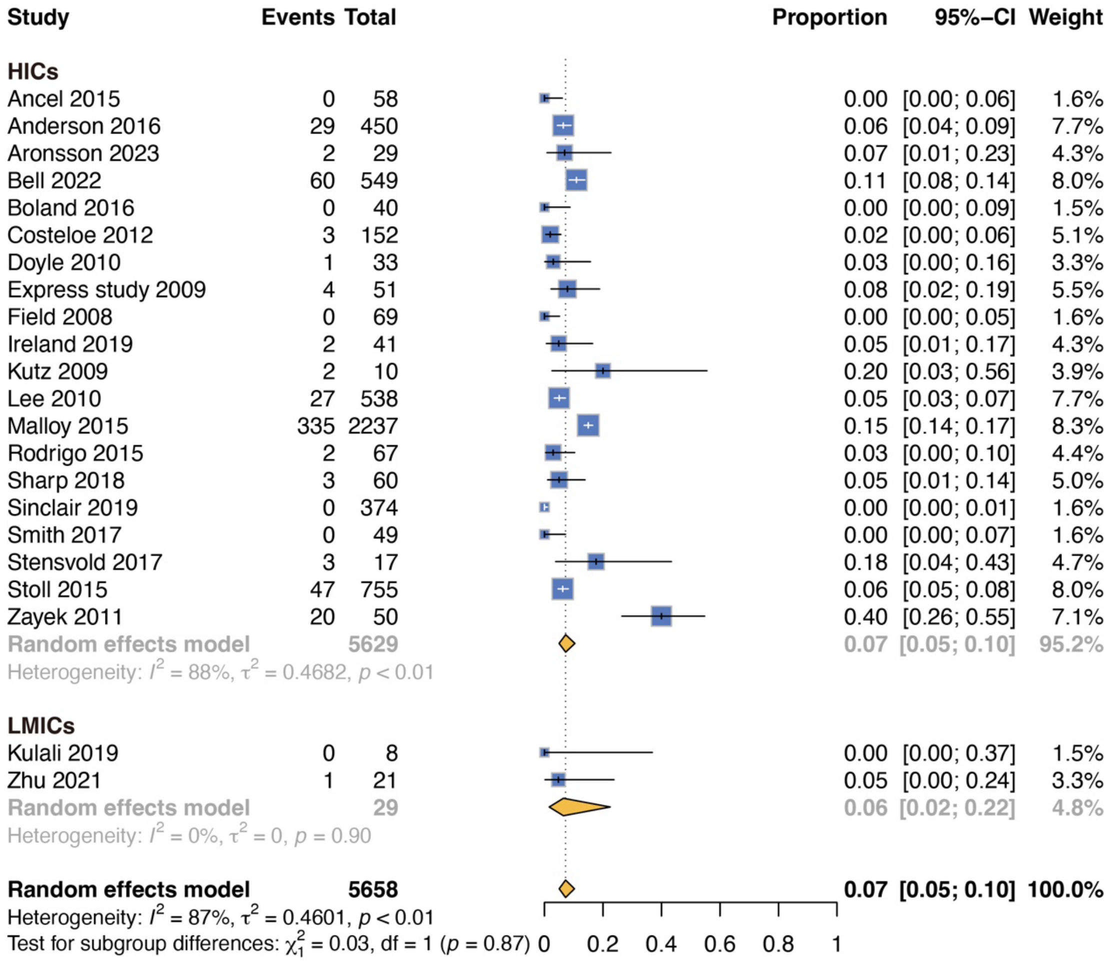

Supplementary Figure 11. Survival rates of periviable infants born at 22 weeks of GA (Live births) across countries with varied income levels

1.23 Supplementary Figure 12

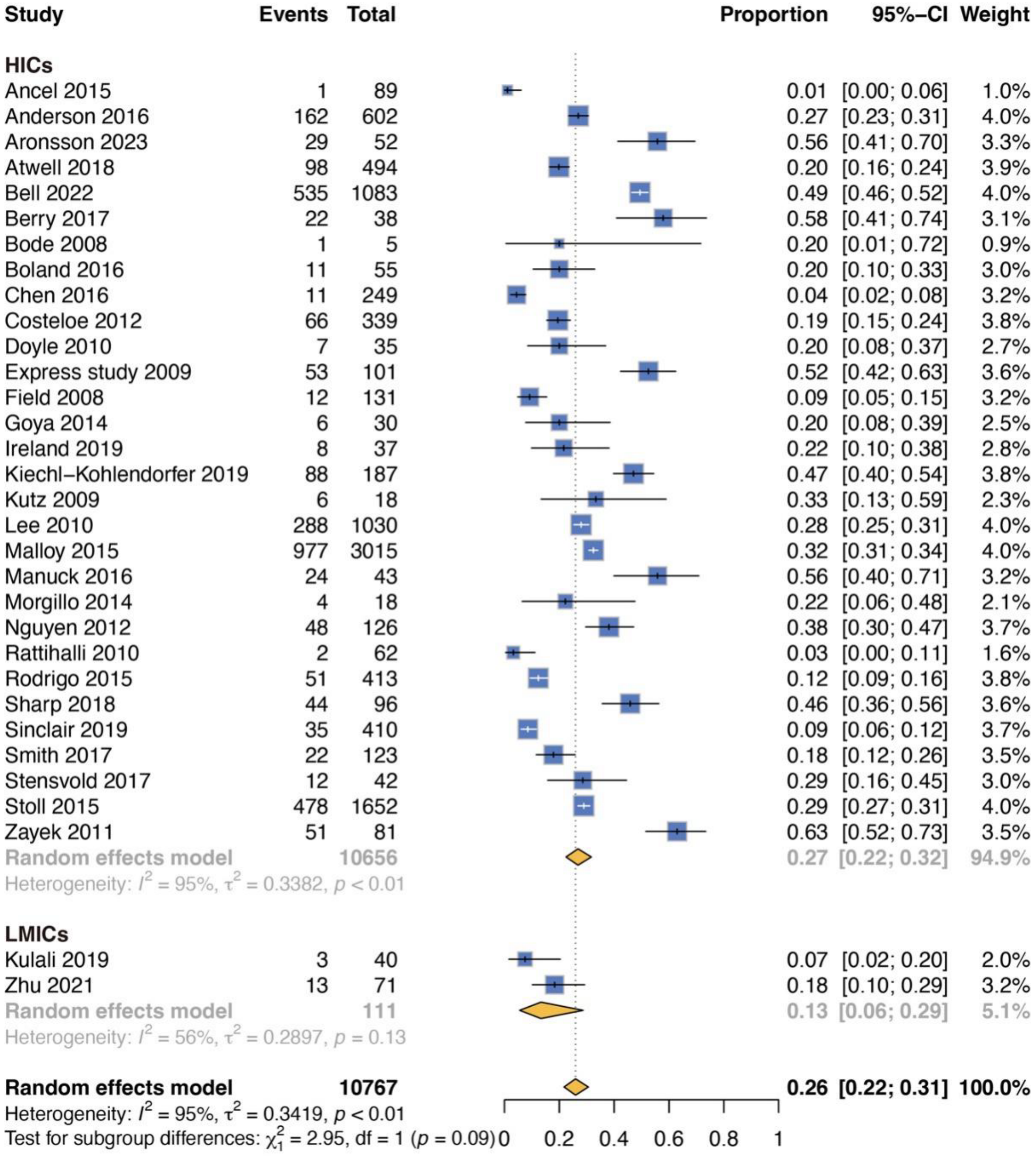

Supplementary Figure 12. Survival rates of periviable infants born at 23 weeks of GA (Live births) across countries with varied income levels

1.24 Supplementary Figure 13

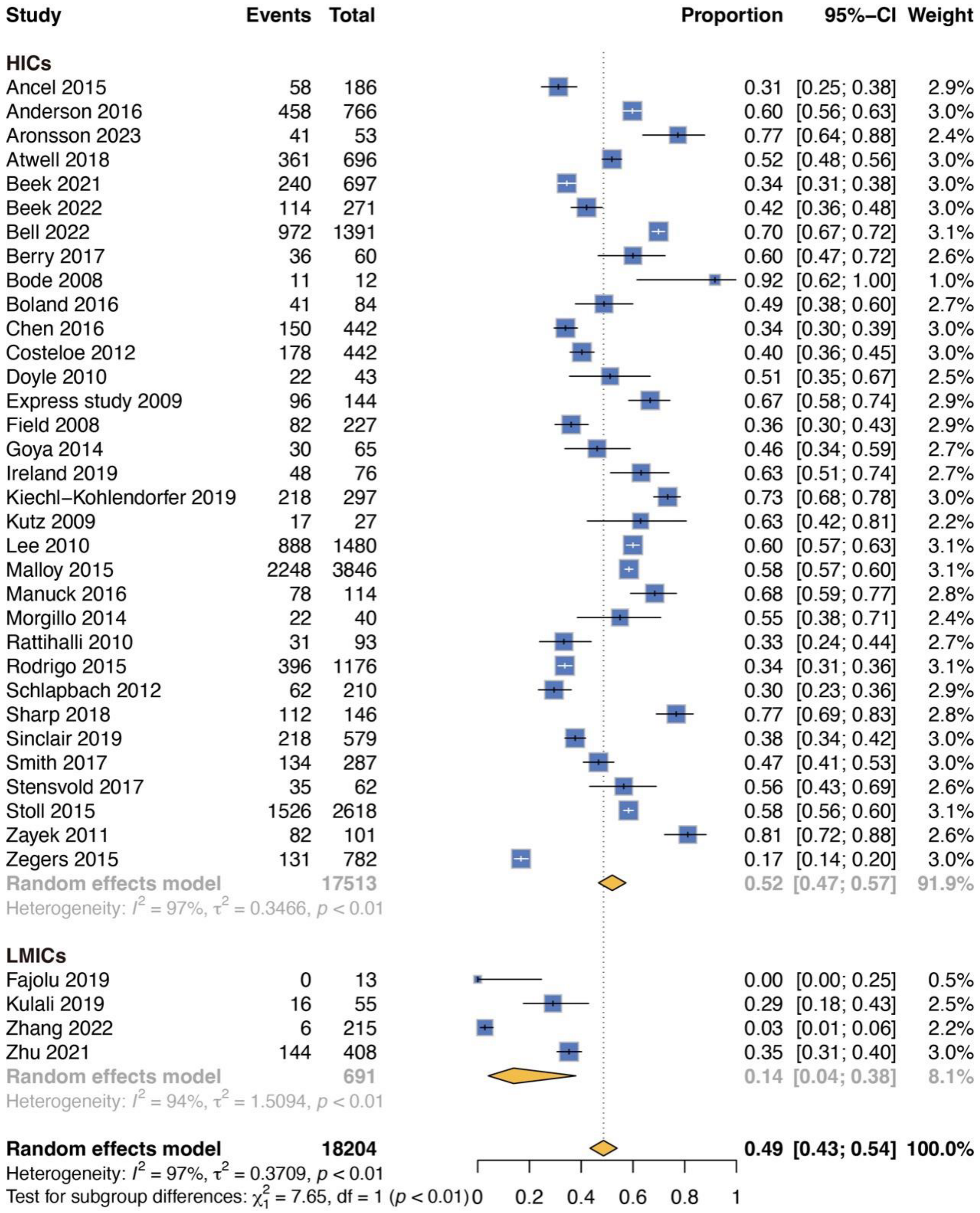

Supplementary Figure 13. Survival rates of periviable infants born at 24 weeks of GA (Live births) across countries with varied income levels

1.25 Supplementary Figure 14

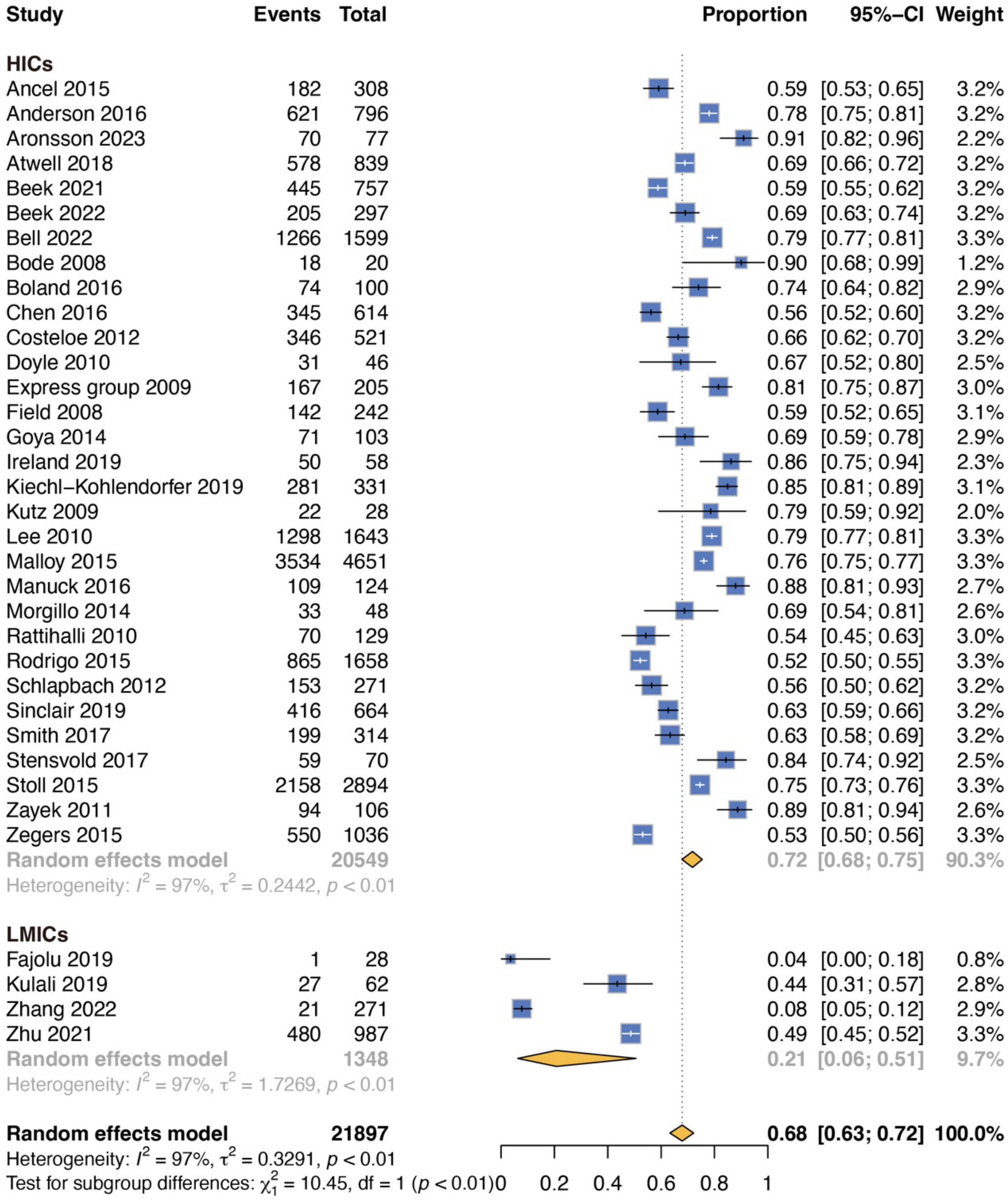

Supplementary Figure 14. Survival rates of periviable infants born at 25 weeks of GA (Live births) across countries with varied income levels

1.26 Supplementary Figure 15

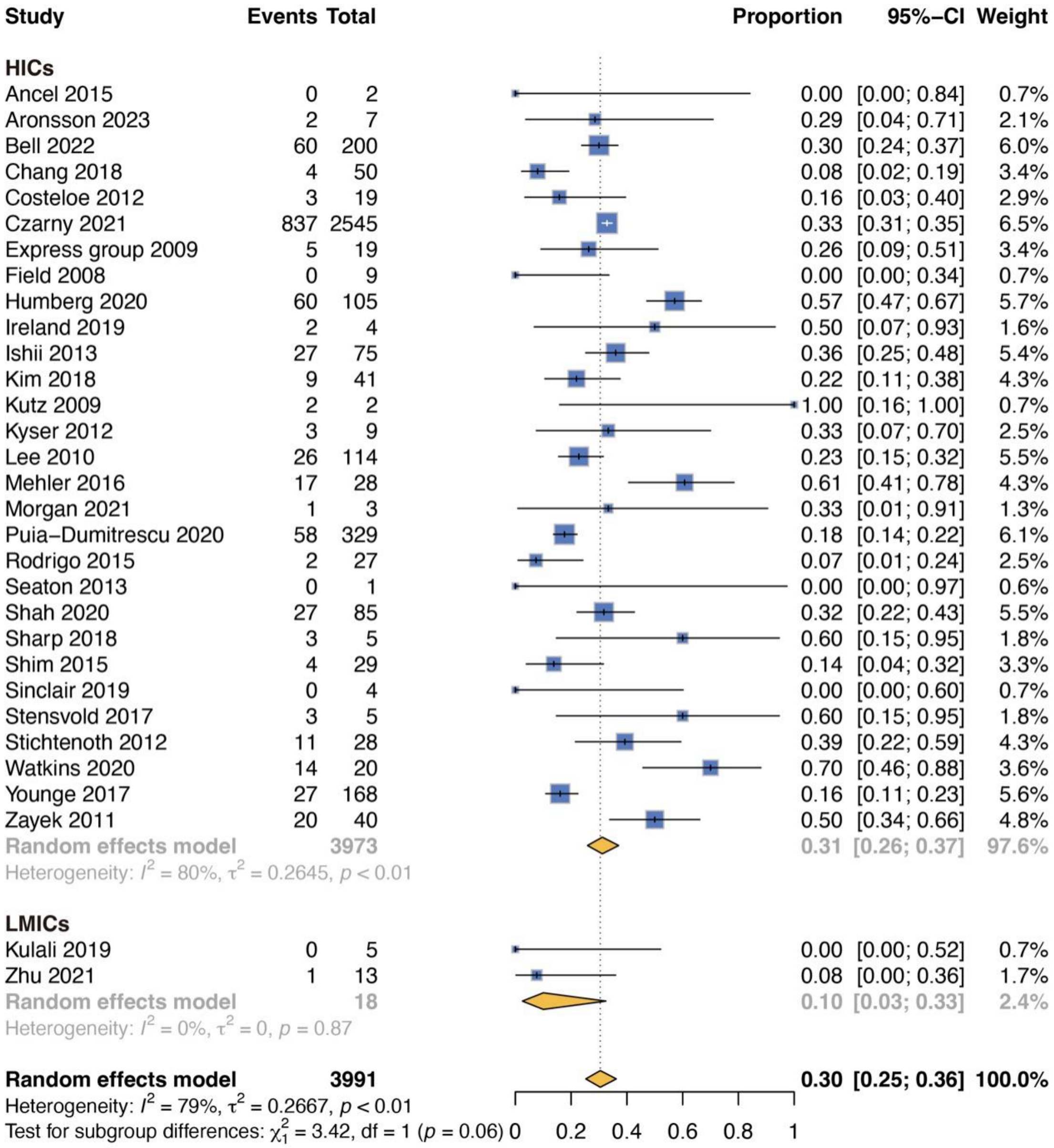

Supplementary Figure 15. Survival rates of periviable infants born at 22 weeks of GA (NICU admissions) across countries with varied income levels

1.27 Supplementary Figure 16

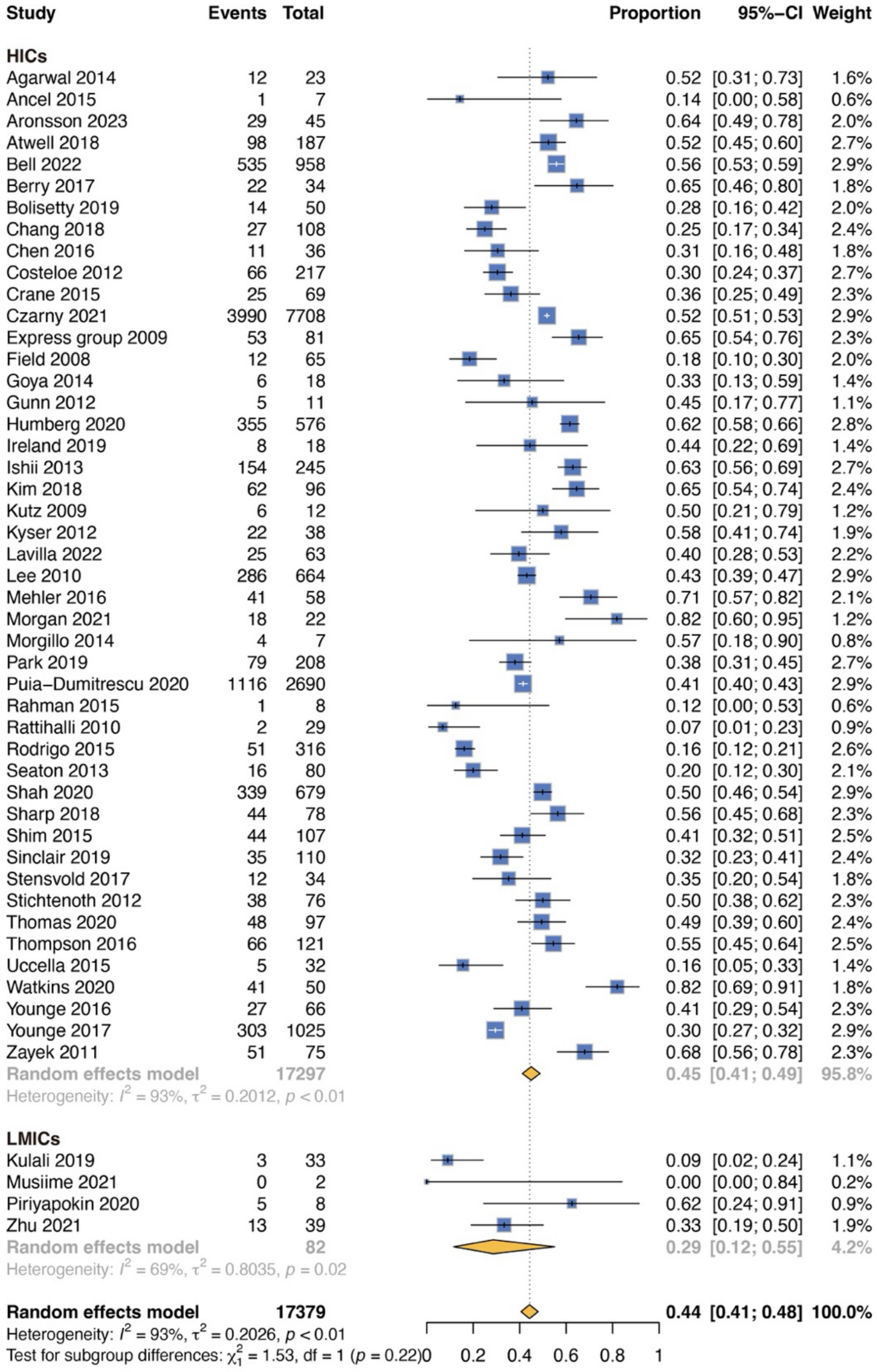

Supplementary Figure 16. Survival rates of periviable infants born at 23 weeks of GA (NICU admissions) across countries with varied income levels

1.28 Supplementary Figure 17

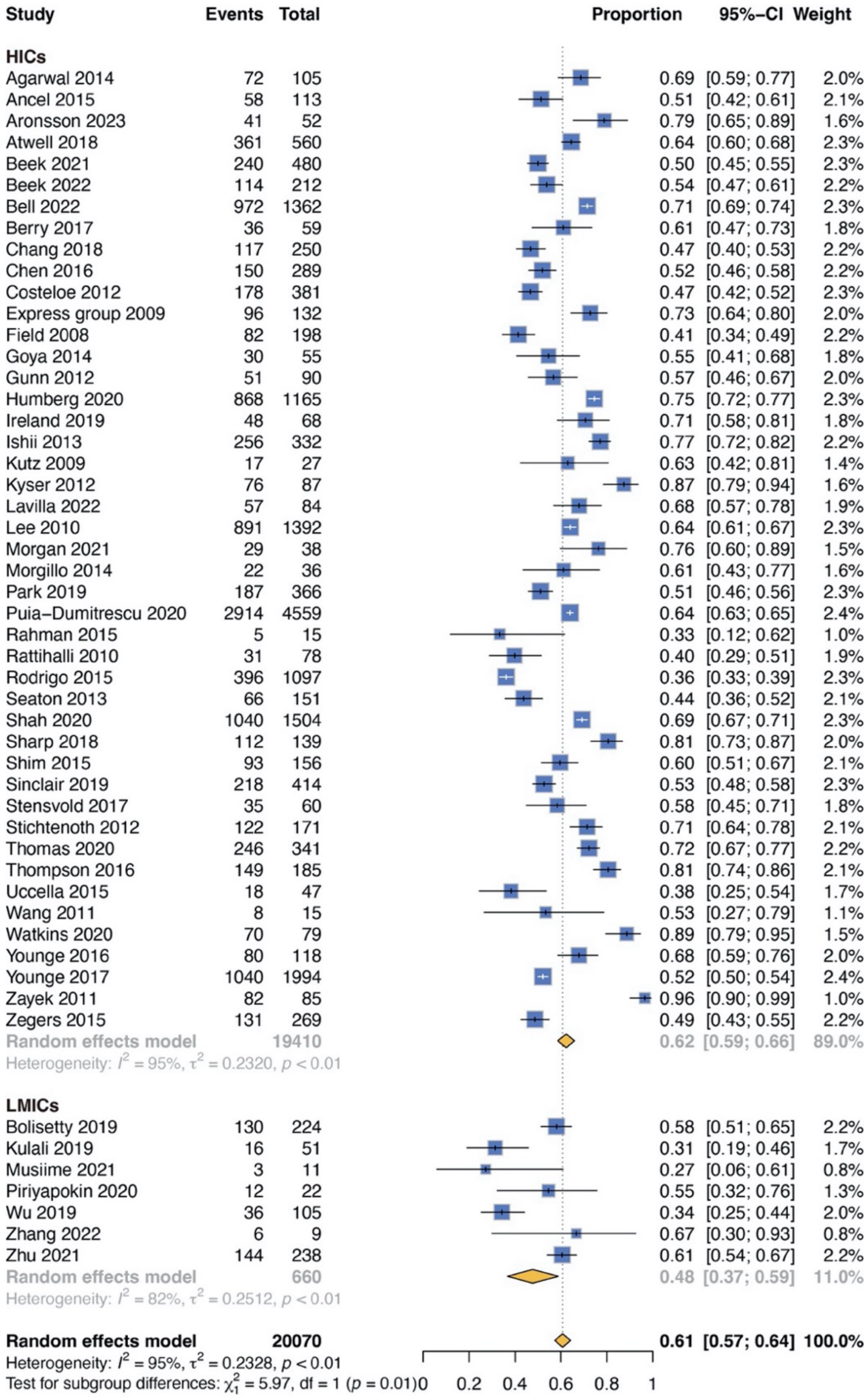

Supplementary Figure 17. Survival rates of periviable infants born at 24 weeks of GA (NICU admissions) across countries with varied income levels

1.29 Supplementary Figure 18

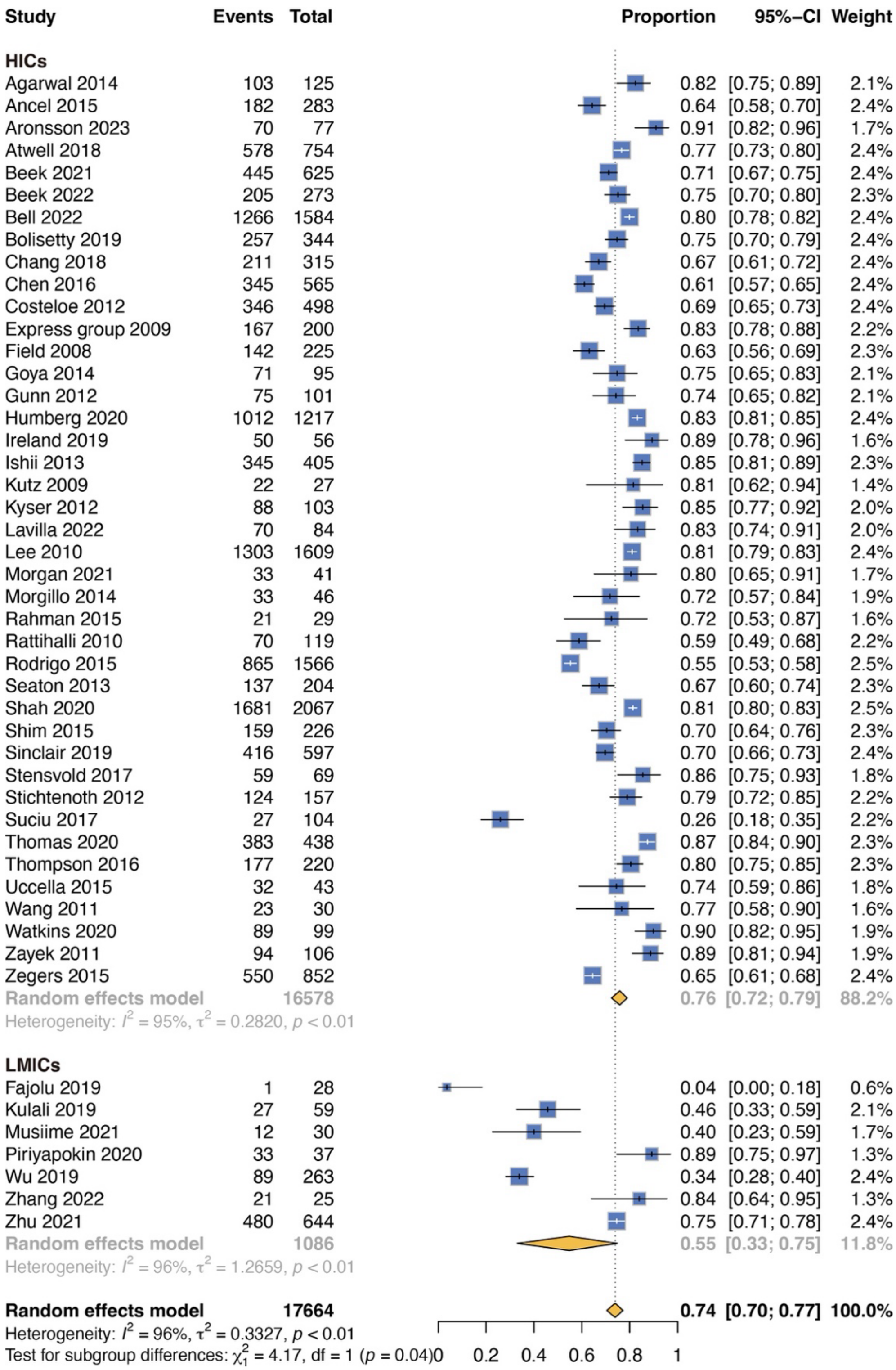

Supplementary Figure 18. Survival rates of periviable infants born at 25 weeks of GA (NICU admissions) across countries with varied income levels

1.30 Supplementary Figure 19

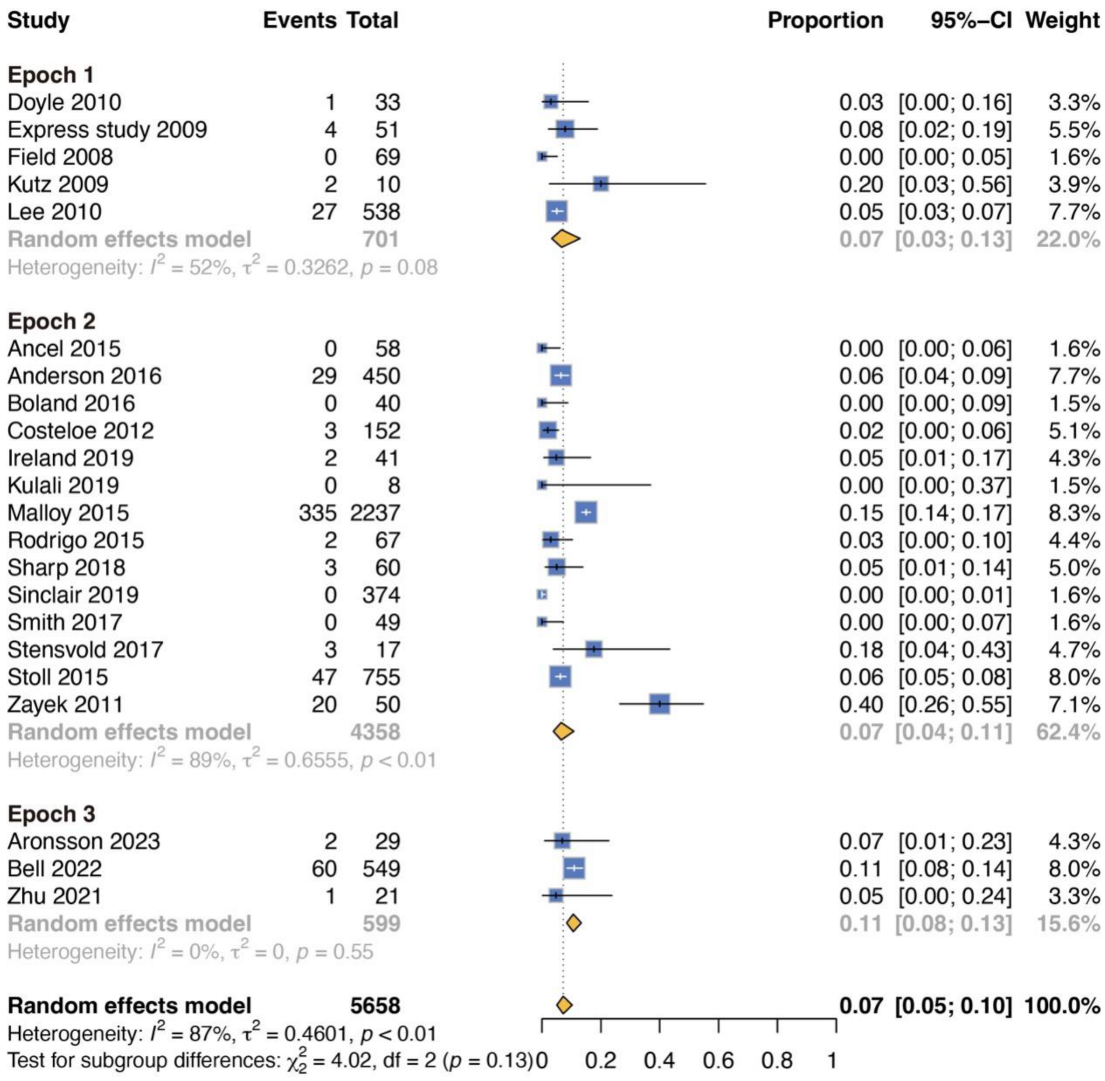

Supplementary Figure 19. Survival rates of periviable infants born at 22 weeks of GA (Live births) across different epochs

1.31 Supplementary Figure 20

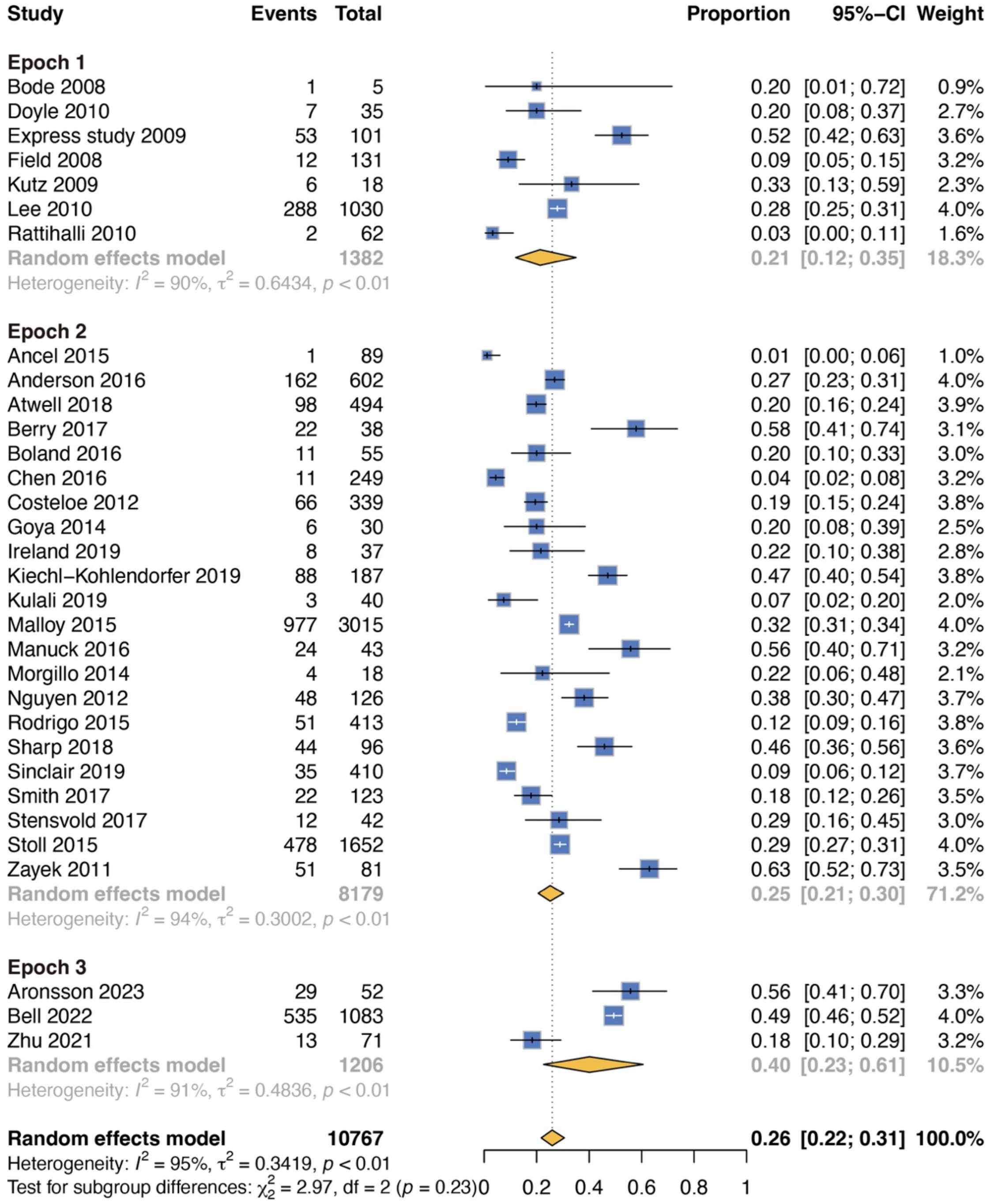

Supplementary Figure 20. Survival rates of periviable infants born at 23 weeks of GA (Live births) across different epochs

1.32 Supplementary Figure 21

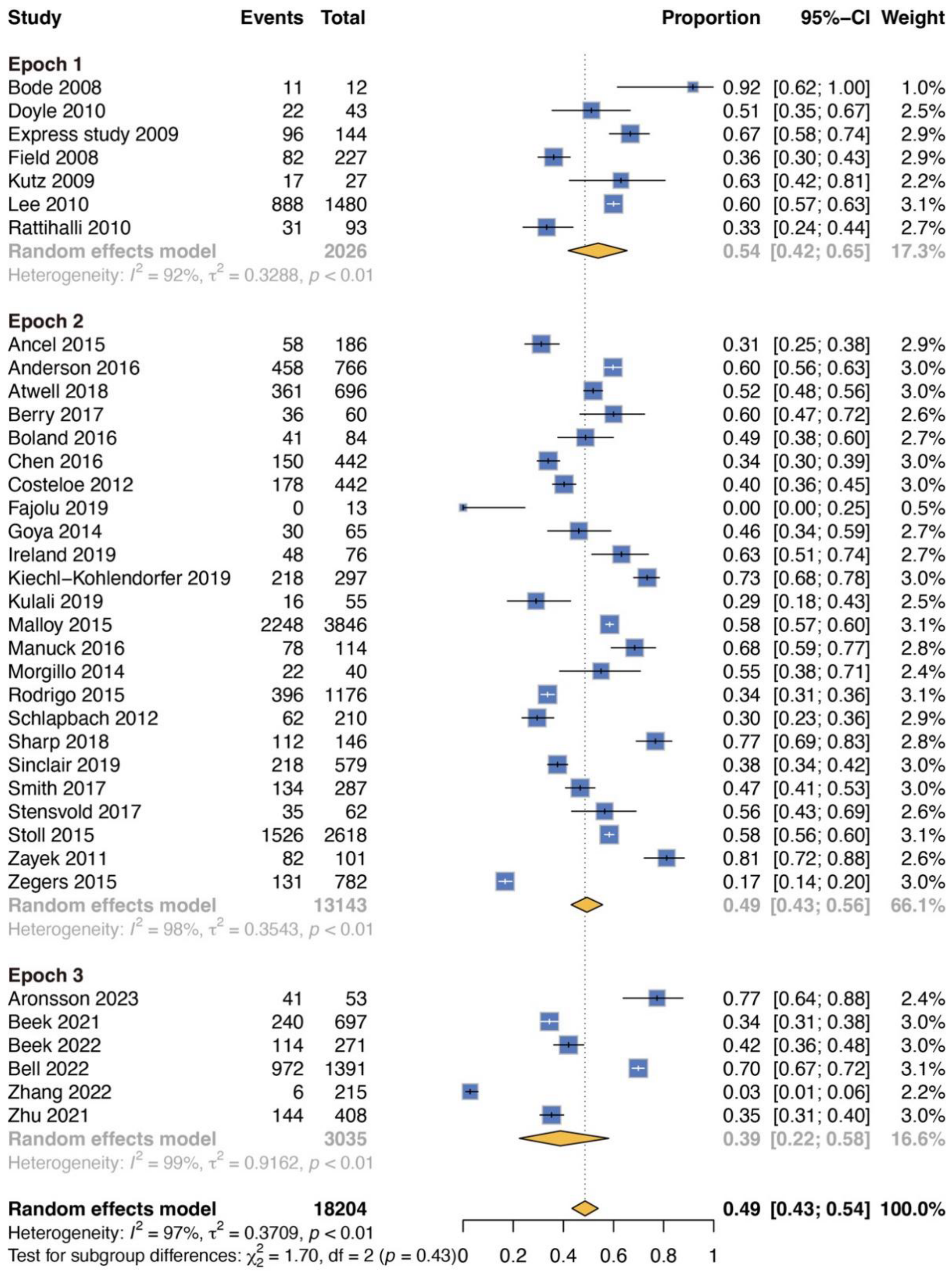

Supplementary Figure 21. Survival rates of periviable infants born at 24 weeks of GA (Live births) across different epochs

1.33 Supplementary Figure 22

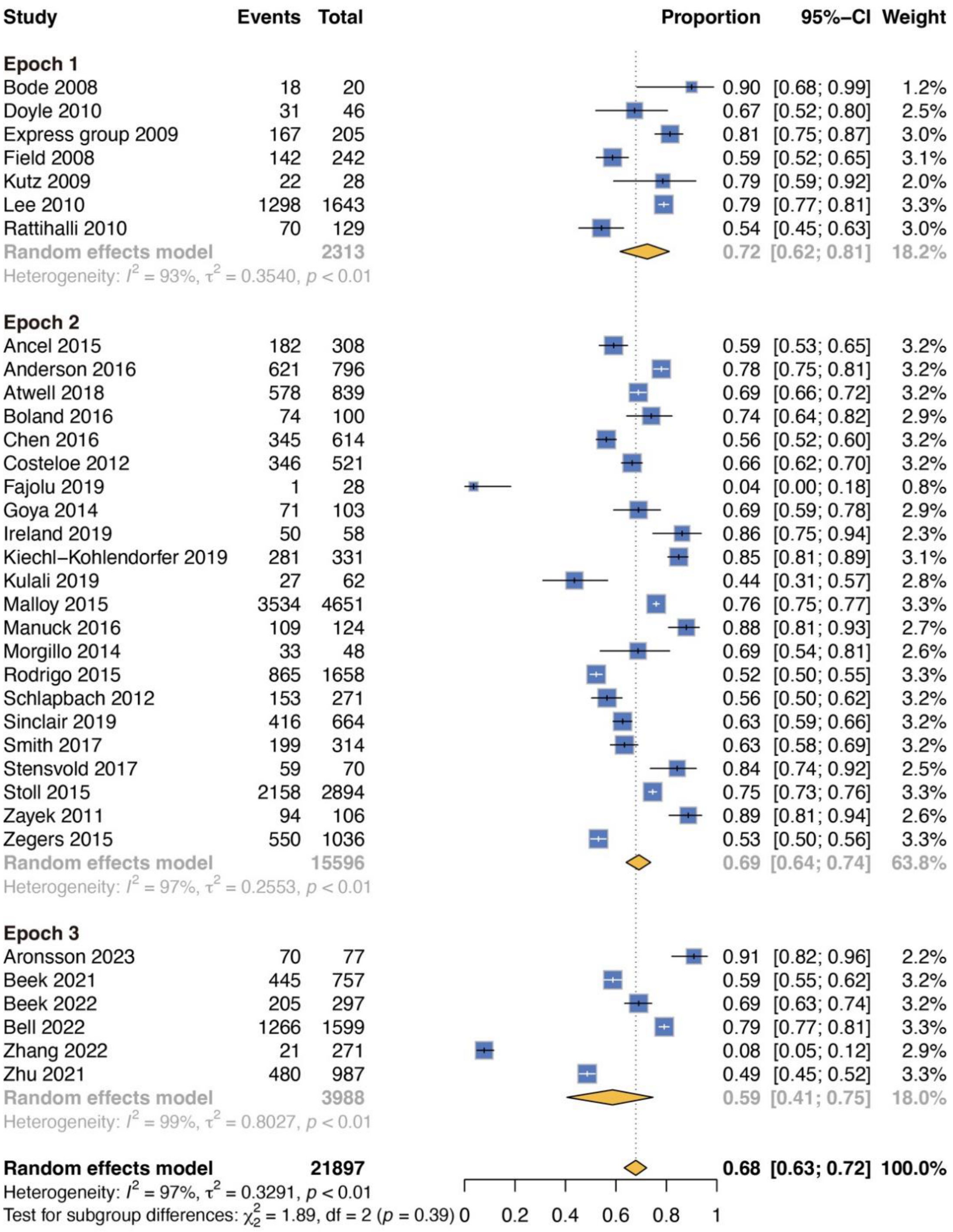

Supplementary Figure 22. Survival rates of periviable infants born at 25 weeks of GA (Live births) across different epochs

1.34 Supplementary Figure 23

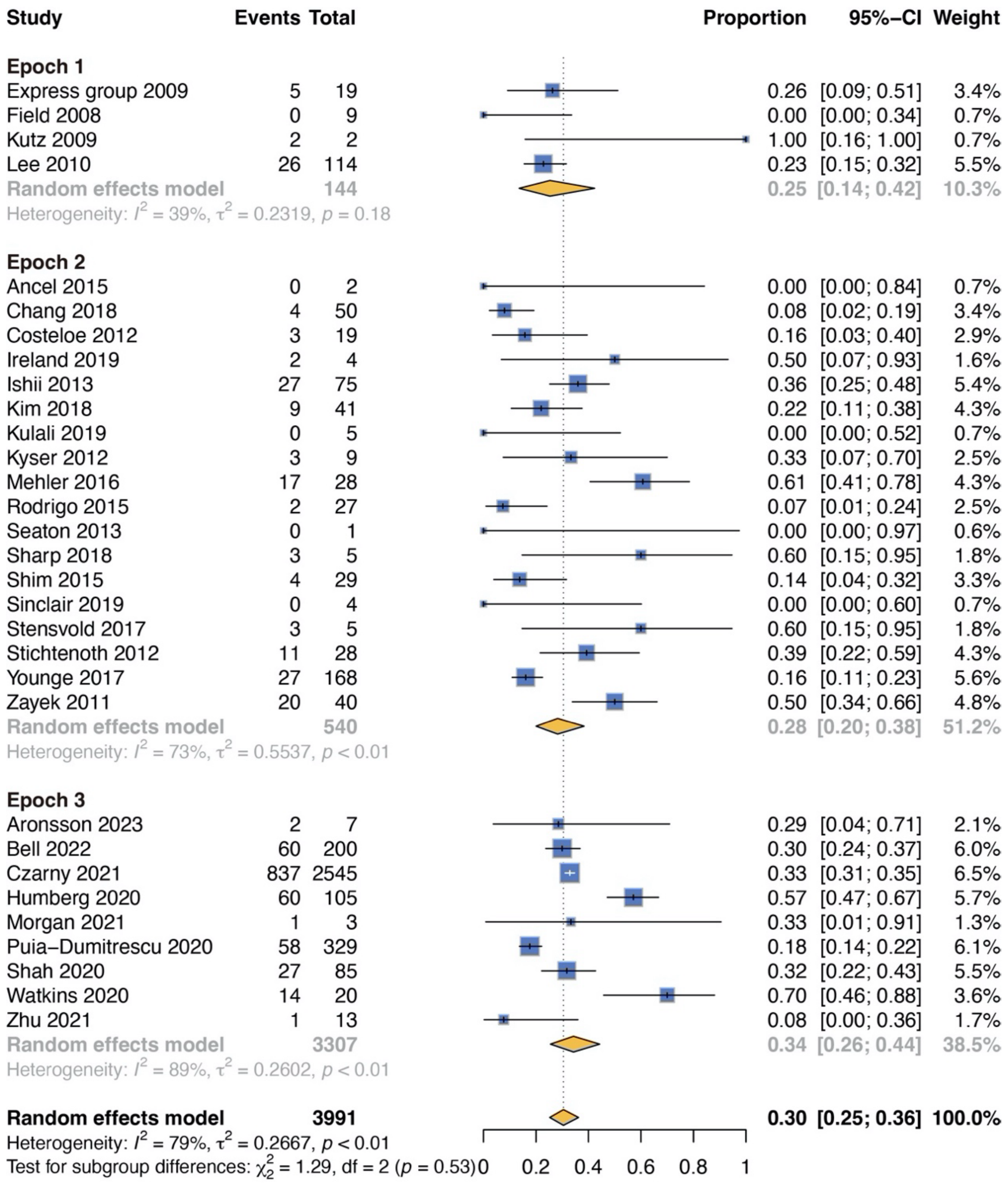

Supplementary Figure 23. Survival rates of periviable infants born at 22 weeks of GA (NICU admissions) across different epochs

1.35 Supplementary Figure 24

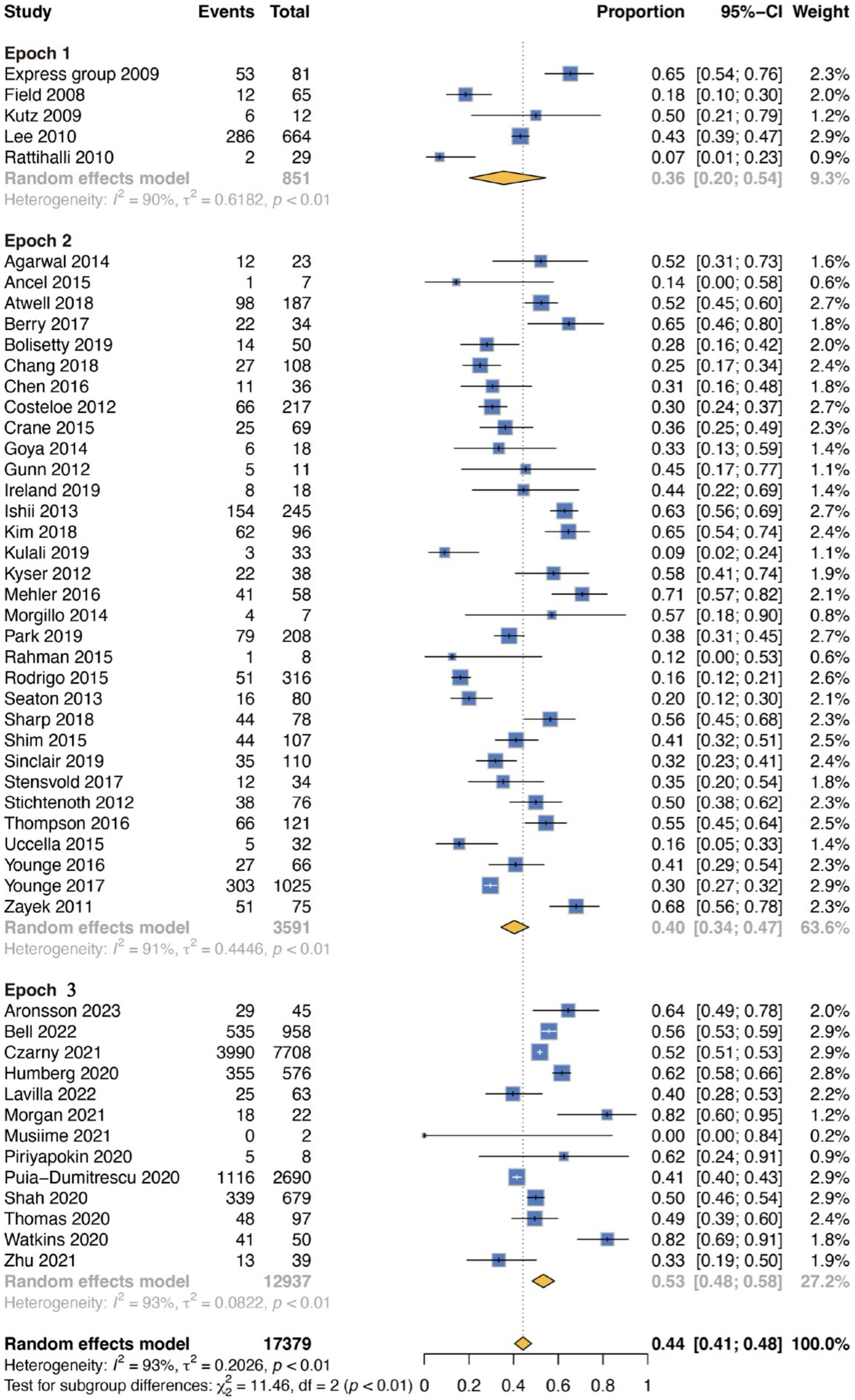

Supplementary Figure 24. Survival rates of periviable infants born at 23 weeks of GA (NICU admissions) across different epochs

1.36 Supplementary Figure 25

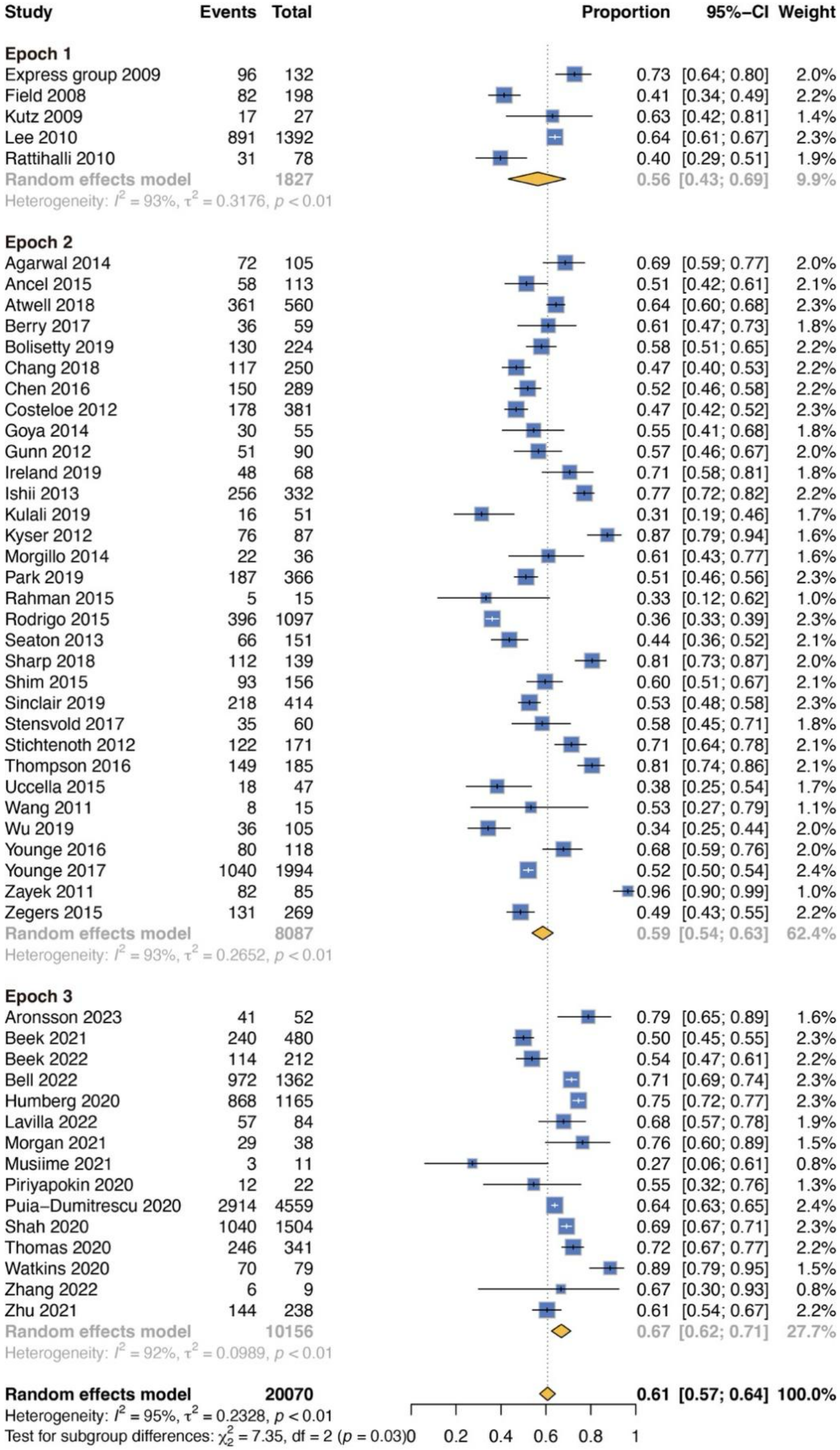

Supplementary Figure 25. Survival rates of periviable infants born at 24 weeks of GA (NICU admissions) across different epochs

1.37 Supplementary Figure 26

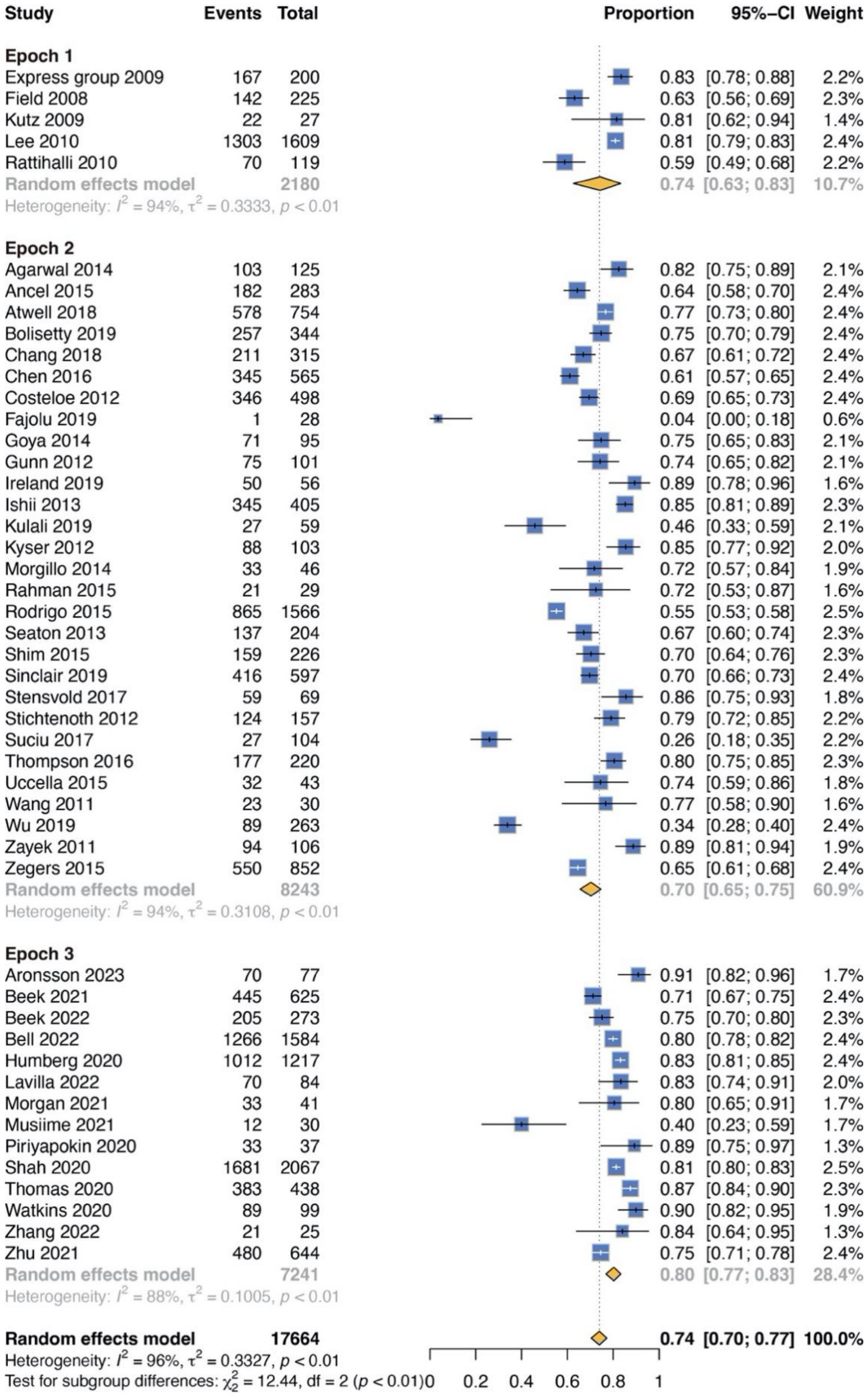

Supplementary Figure 26. Survival rates of periviable infants born at 25 weeks of GA (NICU admissions) across different epochs

1.38 Supplementary Figure 27

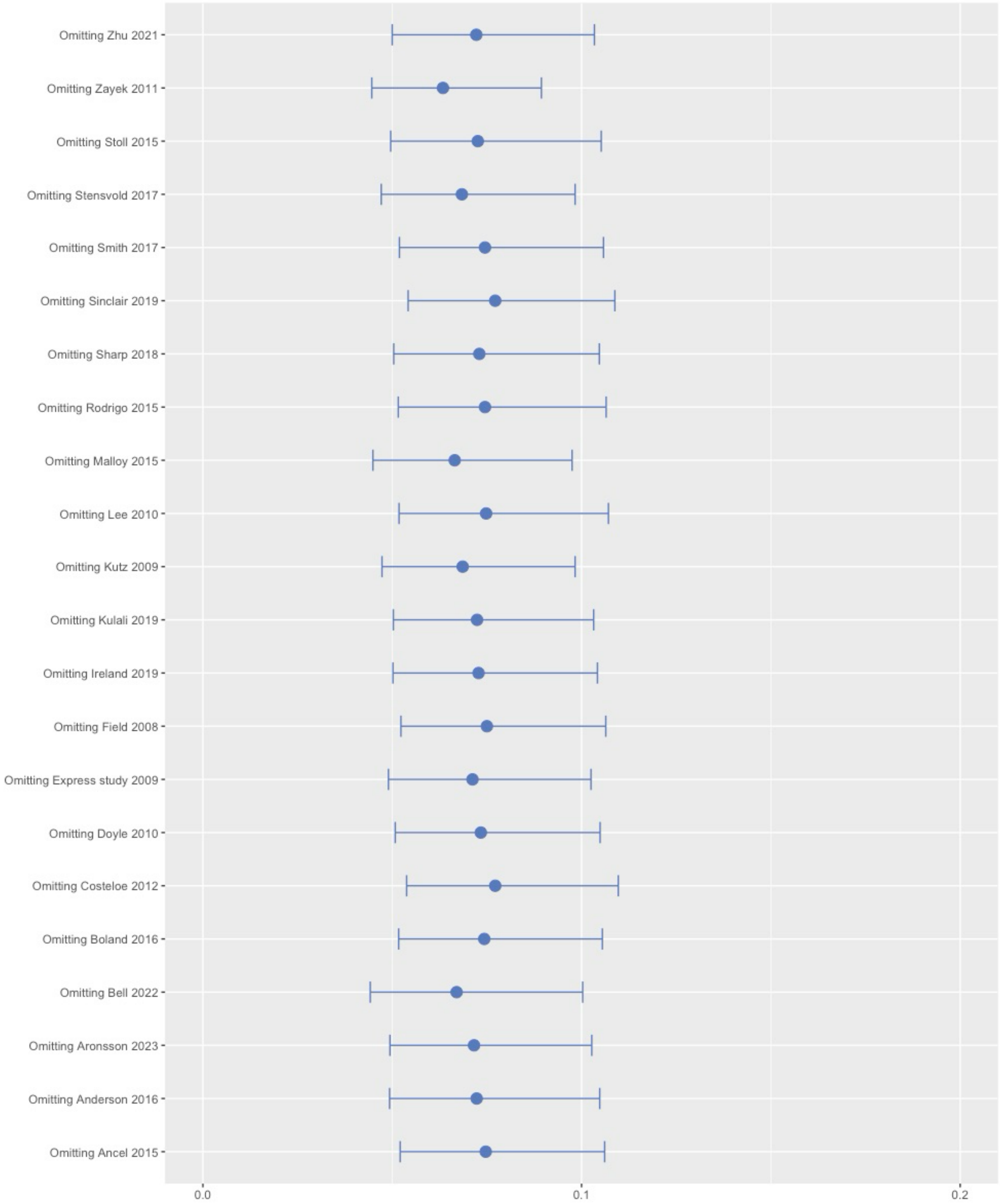

Supplementary Figure 27. Sensitive analysis of periviable infants born at 22 weeks of GA (Live births)

1.39 Supplementary Figure 28

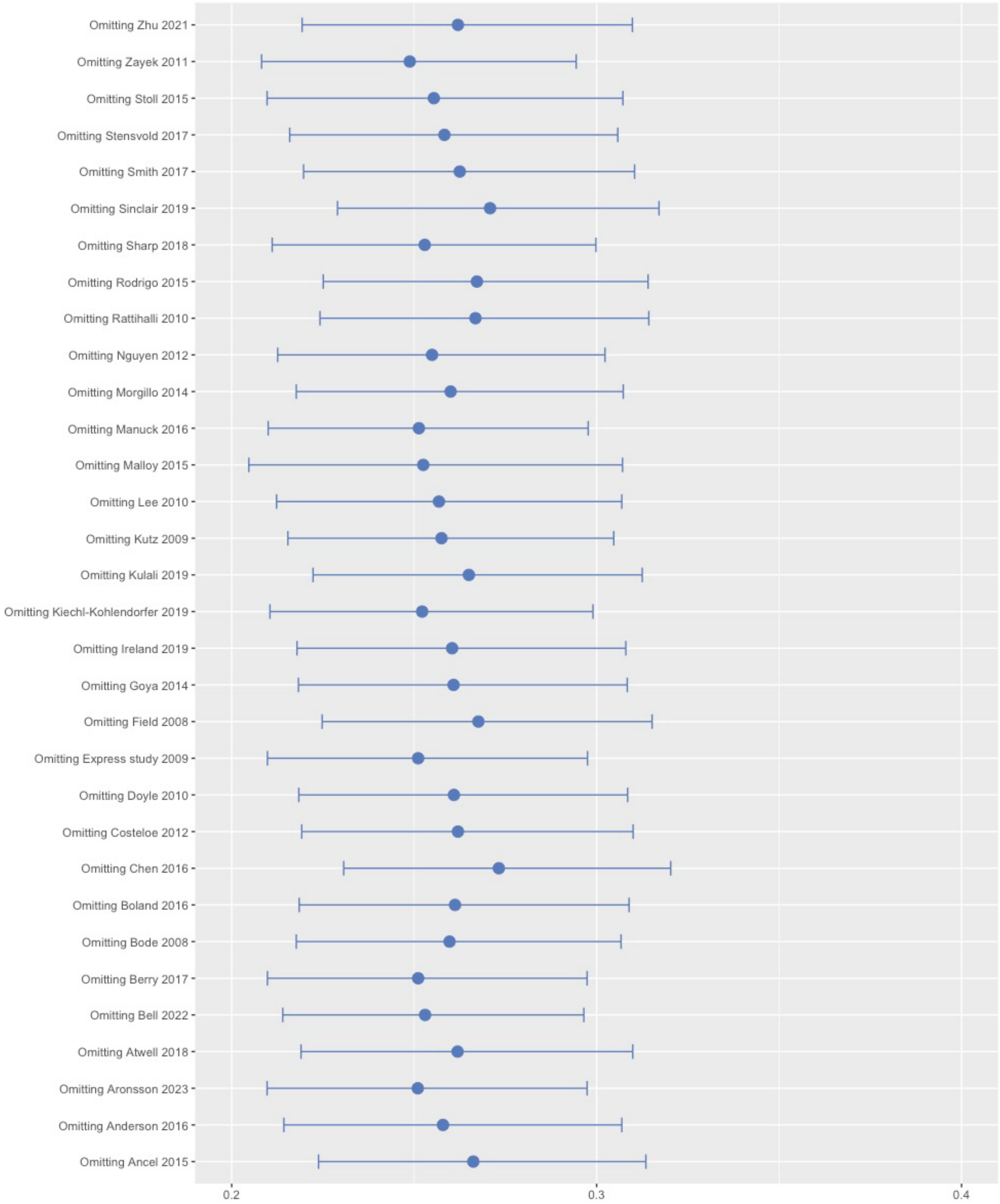

Supplementary Figure 28. Sensitive analysis of periviable infants born at 23 weeks of GA (Live births)

1.40 Supplementary Figure 29

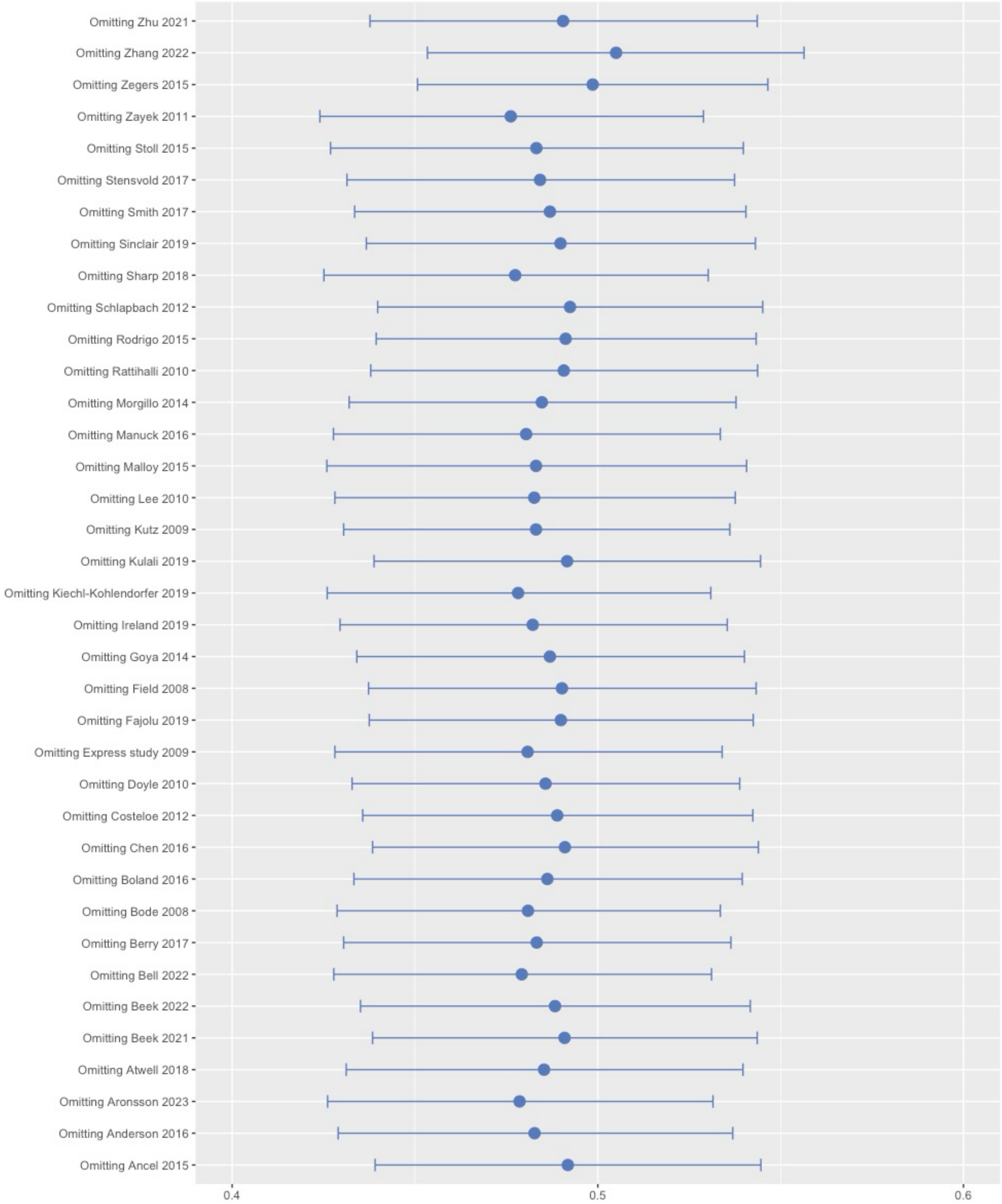

Supplementary Figure 29. Sensitive analysis of periviable infants born at 24 weeks of GA (Live births)

1.41 Supplementary Figure 30

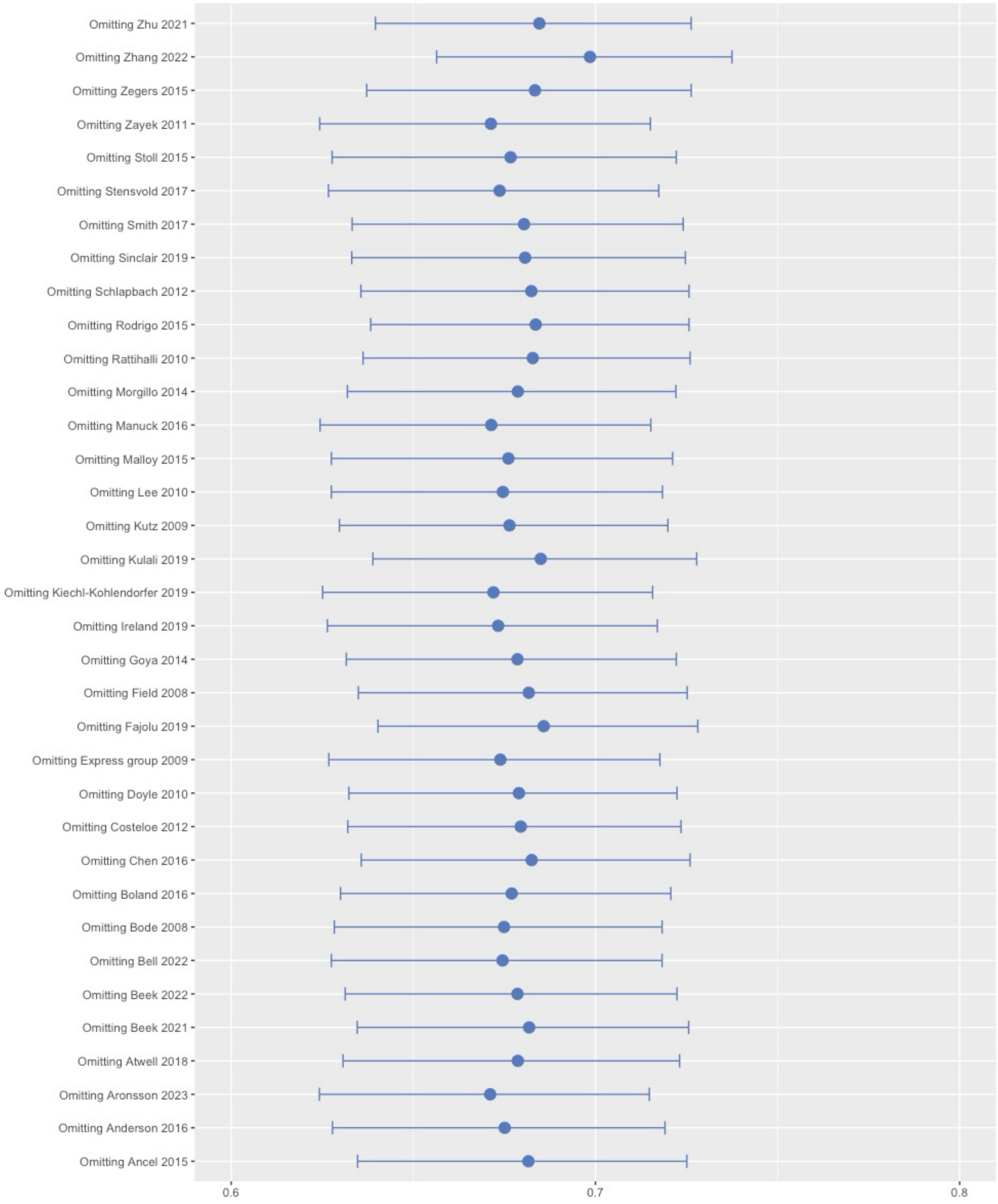

Supplementary Figure 30. Sensitive analysis of periviable infants born at 25 weeks of GA (Live births)

1.42 Supplementary Figure 31

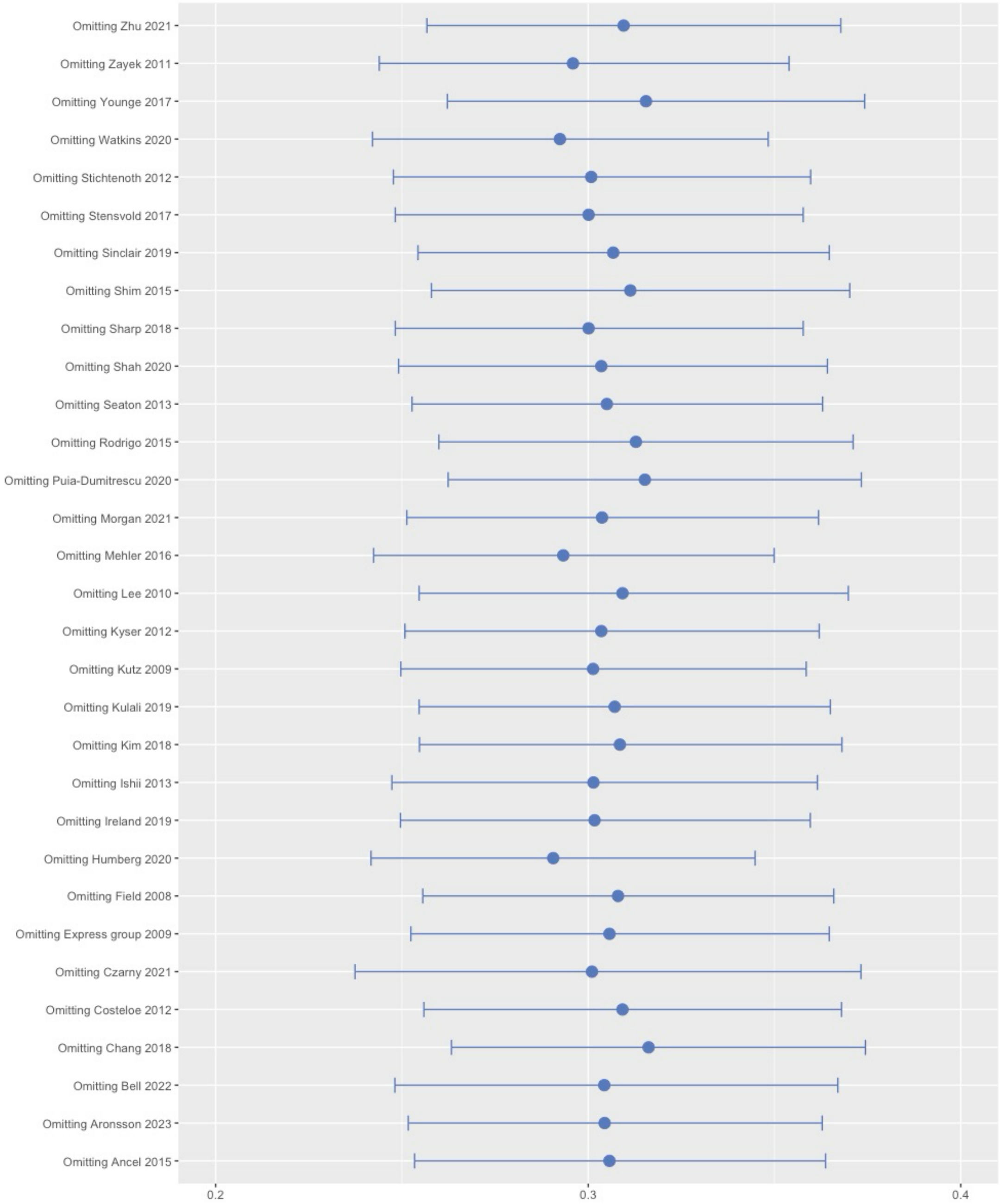

Supplementary Figure 31. Sensitive analysis of periviable infants born at 22 weeks of GA (NICU admissions)

1.43 Supplementary Figure 32

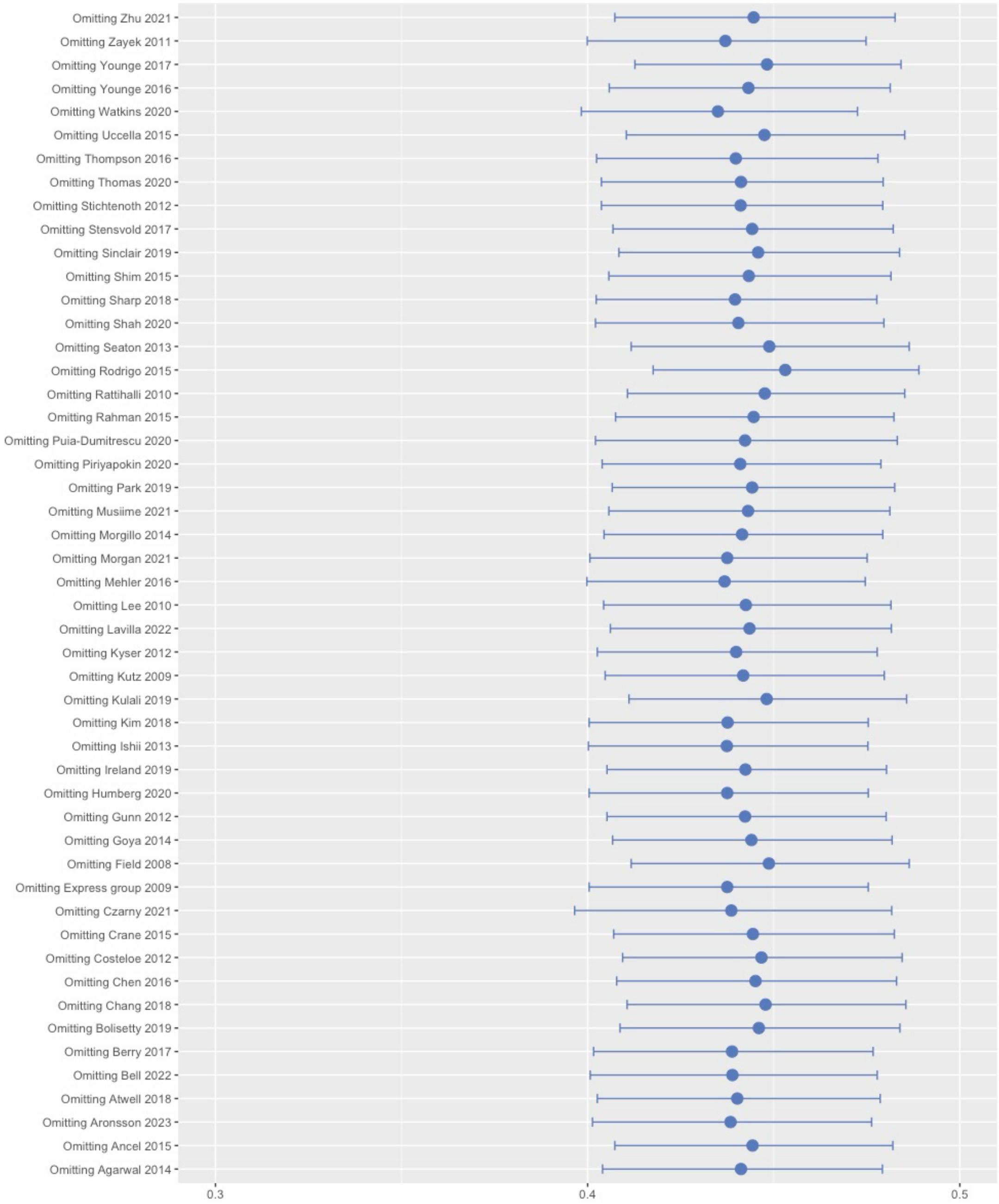

Supplementary Figure 32. Sensitive analysis of periviable infants born at 23 weeks of GA (NICU admissions)

1.44 Supplementary Figure 33

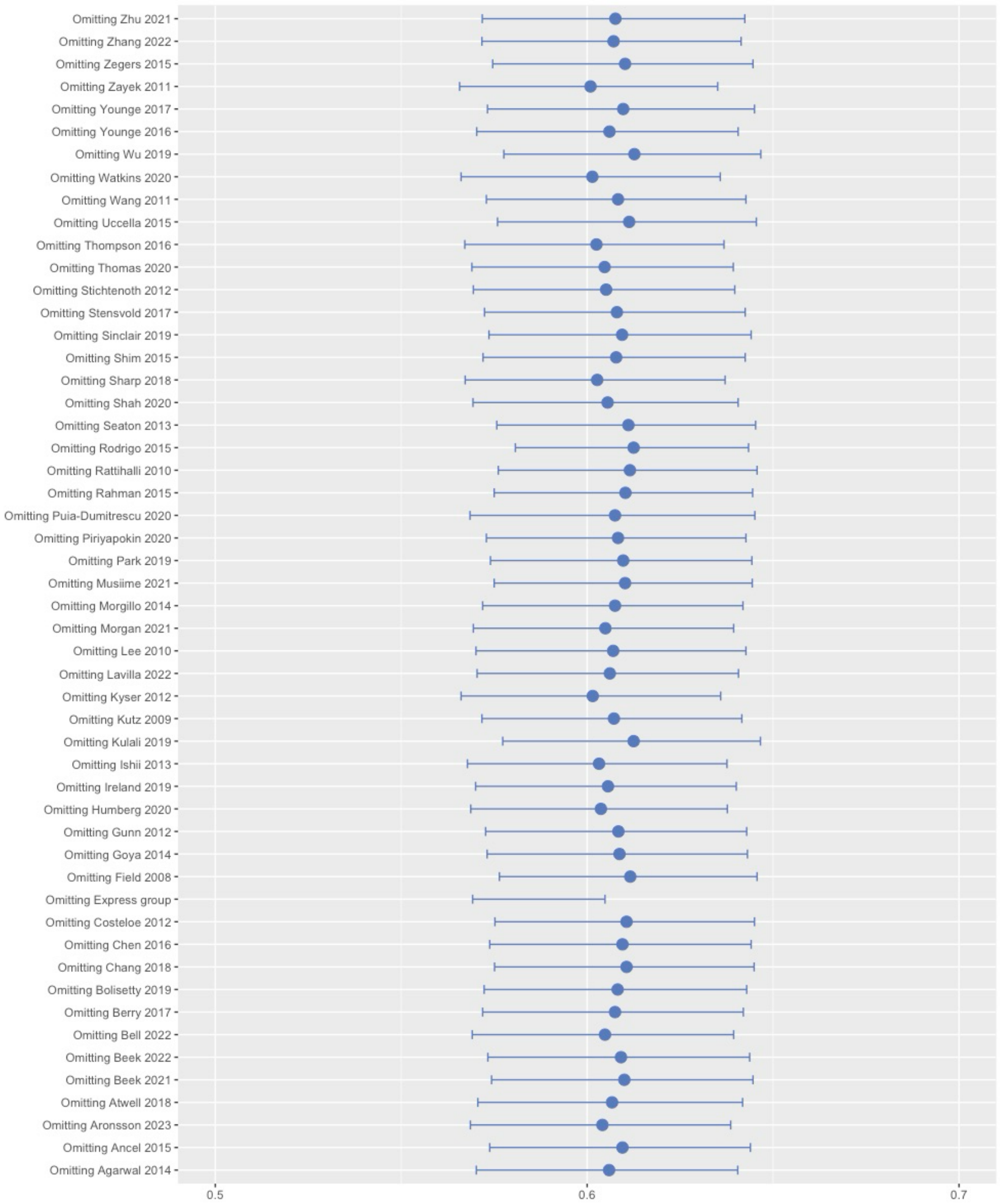

Supplementary Figure 33. Sensitive analysis of periviable infants born at 24 weeks of GA (NICU admissions)

1.45 Supplementary Figure 34

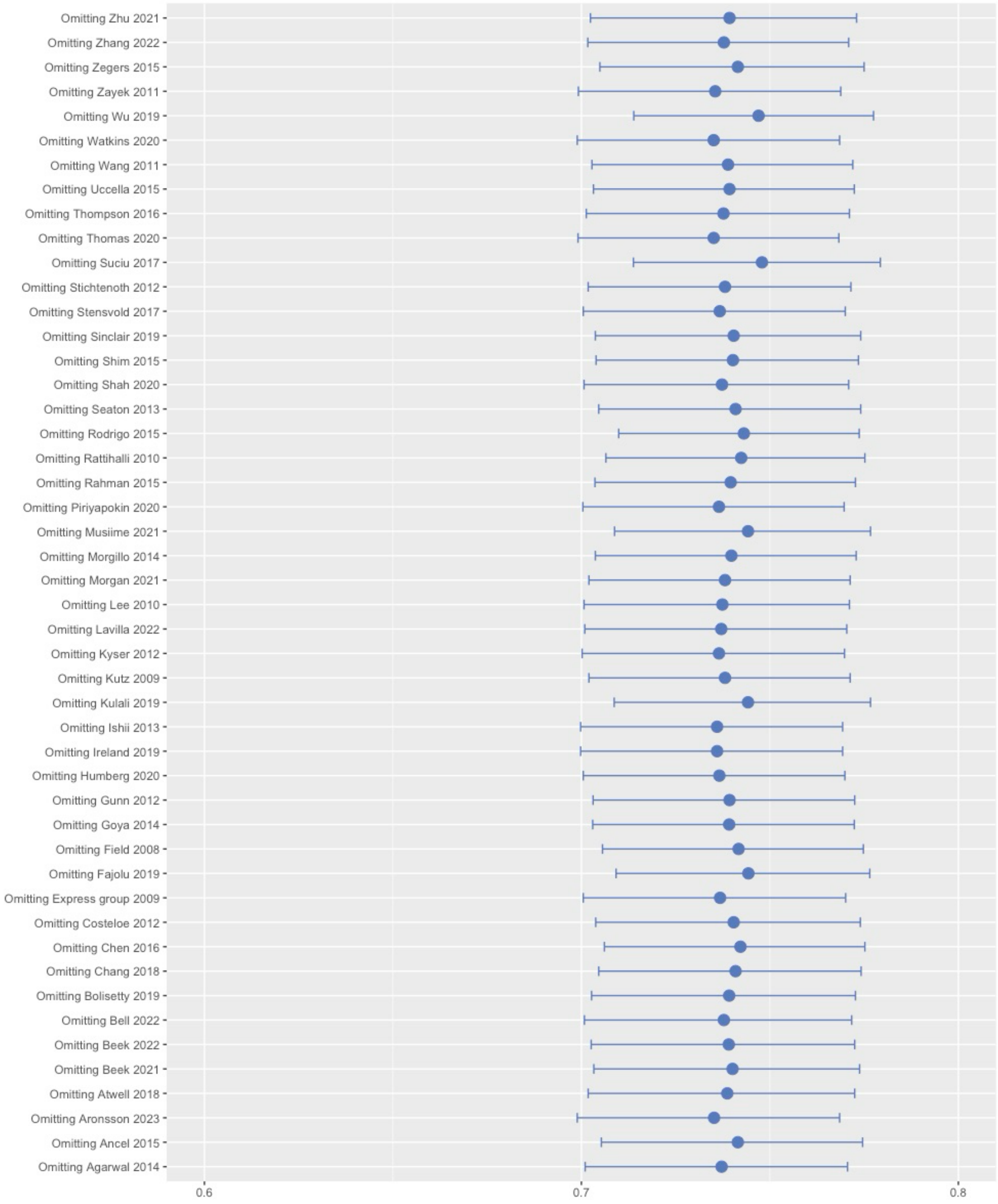

Supplementary Figure 34. Sensitive analysis of periviable infants born at 25 weeks of GA (NICU admissions)

1.46 Supplementary Figure 35

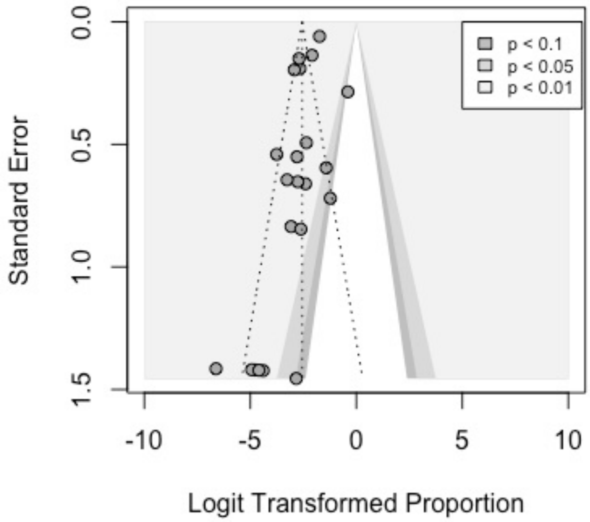

Supplementary Figure 35. Contour-enhanced funnel plot of infants born at 22 weeks of GA (Live births)

1.47 Supplementary Figure 36

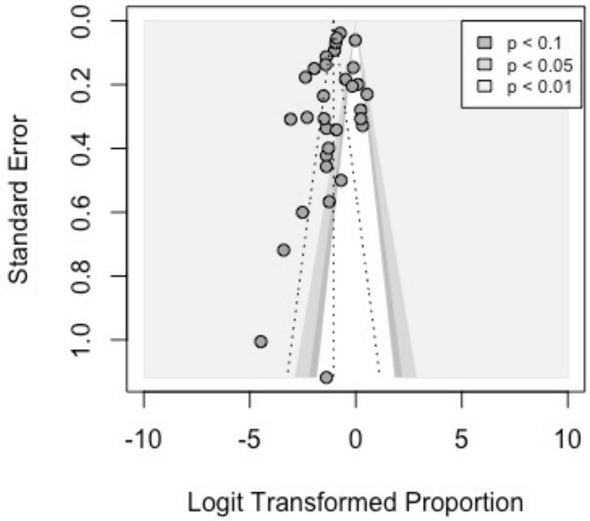

Supplementary Figure 36. Contour-enhanced funnel plot of periviable infants born at 23 weeks of GA (Live births)

1.48 Supplementary Figure 37

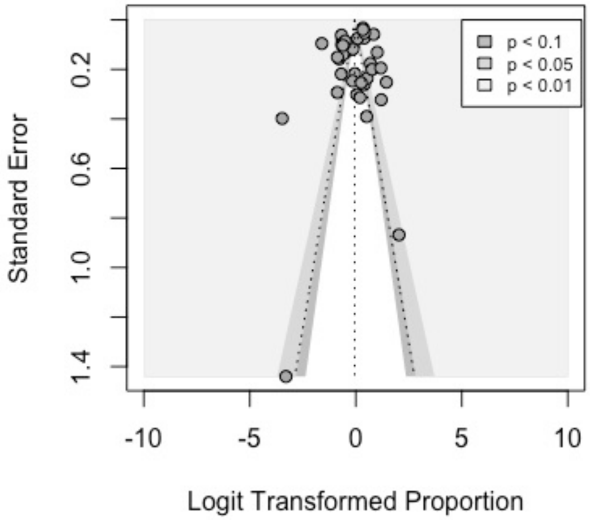

Supplementary Figure 37. Contour-enhanced funnel plot of periviable infants born at 24 weeks of GA (Live births)

1.49 Supplementary Figure 38

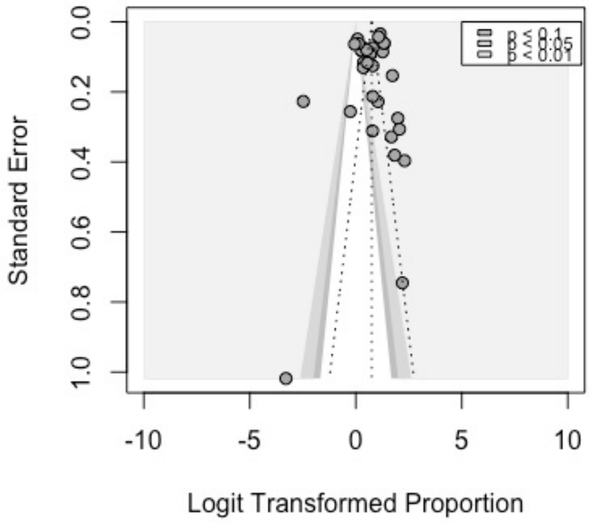

Supplementary Figure 38. Contour-enhanced funnel plot of periviable infants born at 25 weeks of GA (Live births)

1.50 Supplementary Figure 39

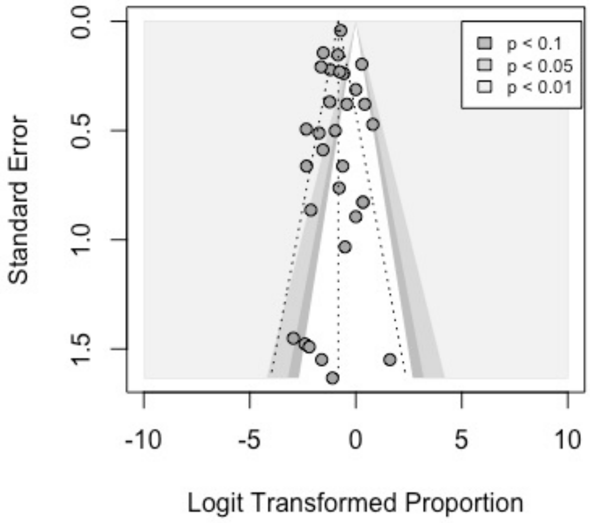

Supplementary Figure 39. Contour-enhanced funnel plot of periviable infants born at 22 weeks of GA (NICU admissions)

1.51 Supplementary Figure 40

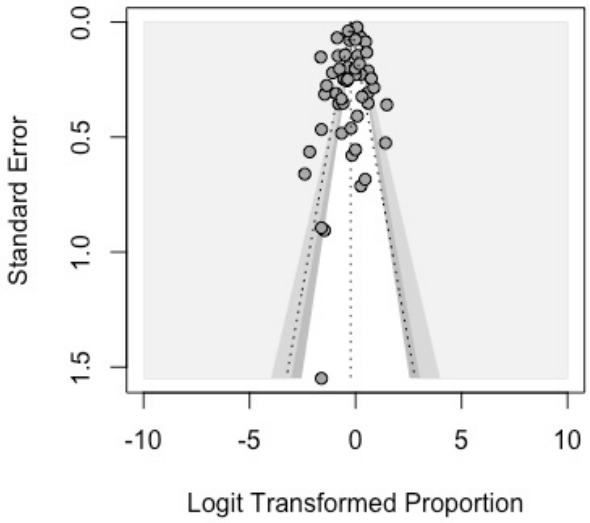

Supplementary Figure 40. Contour-enhanced funnel plot of periviable infants born at 23 weeks of GA (NICU admissions)

1.52 Supplementary Figure 41

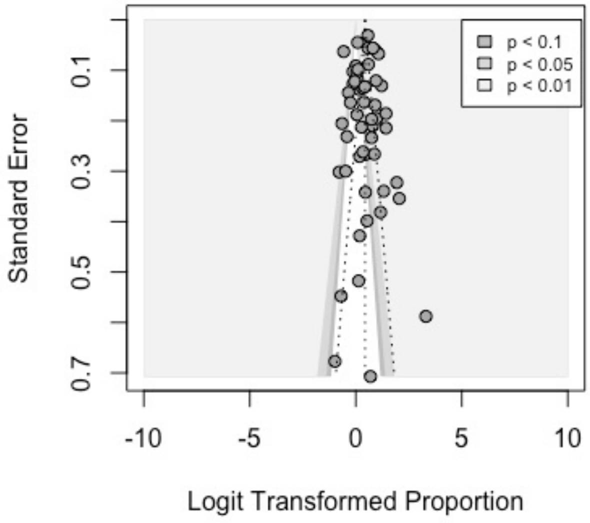

Supplementary Figure 41. Contour-enhanced funnel plot of periviable infants born at 24 weeks of GA (NICU admissions)

1.53 Supplementary Figure 42

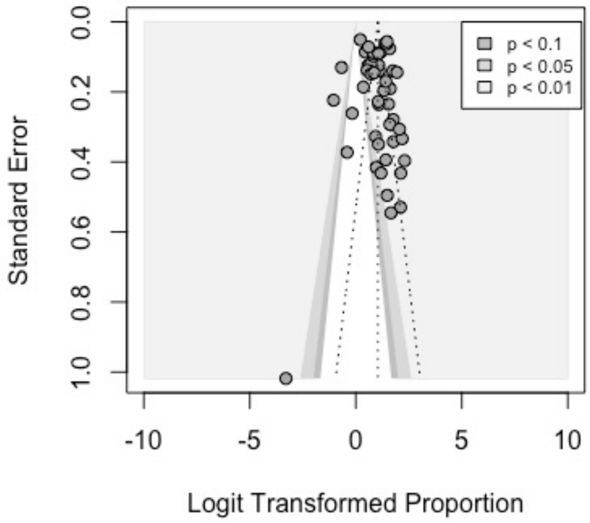

Supplementary Figure 42. Contour-enhanced funnel plot of periviable infants born at 25 weeks of GA (NICU admissions)
